# A Non-Inferiority, Individually Randomized Trial of Intermittent Screening and Treatment versus Intermittent Preventive Treatment in the Control of Malaria in Pregnancy

**DOI:** 10.1371/journal.pone.0132247

**Published:** 2015-08-10

**Authors:** Harry Tagbor, Matthew Cairns, Kalifa Bojang, Sheick Oumar Coulibaly, Kassoum Kayentao, John Williams, Ismaela Abubakar, Francis Akor, Khalifa Mohammed, Richard Bationo, Edgar Dabira, Alamissa Soulama, Moussa Djimdé, Etienne Guirou, Timothy Awine, Stephen Quaye, Fanta Njie, Jaume Ordi, Ogobara Doumbo, Abraham Hodgson, Abraham Oduro, Steven Meshnick, Steve Taylor, Pascal Magnussen, Feiko ter Kuile, Arouna Woukeu, Paul Milligan, Daniel Chandramohan, Brian Greenwood

**Affiliations:** 1 London School of Hygiene & Tropical Medicine, London, United Kingdom; 2 School of Public Health, Kwame Nkrumah University of Science and Technology, Kumasi, Ghana; 3 Medical Research Council Unit, Fajara, The Gambia; 4 Faculty of Health Sciences, University of Ouagadougou, Ouagadougou, Burkina Faso; 5 Malaria Research and Training Centre, Faculty of Medicine and Odonto-stomatology, University of Sciences, Technics and Technologies, Bamako, Mali; 6 Navrongo Health Research Centre, Navrongo, Ghana; 7 Barcelona Centre for International Health Research (CRESIB), Department of Pathology, Hospital Clinic-Universitat de Barcelona, Barcelona, Spain; 8 Gillings School of Global Public Health, University of North Carolina, Chapel Hill, NC, United States of America; 9 Duke University Medical Center, Durham, NC, United States of America; 10 (Institute of International Health, Immunology and Microbiology and Institute of Veterinary Disease Biology, University of Copenhagen, Copenhagen, Denmark; 11 Liverpool School of Tropical Medicine, Liverpool, United Kingdom; Centers for Disease Control and Prevention, UNITED STATES

## Abstract

**Background:**

The efficacy of intermittent preventive treatment for malaria with sulfadoxine-pyrimethamine (IPTp-SP) in pregnancy is threatened in parts of Africa by the emergence and spread of resistance to SP. Intermittent screening with a rapid diagnostic test (RDT) and treatment of positive women (ISTp) is an alternative approach.

**Methods and Findings:**

An open, individually randomized, non-inferiority trial of IPTp-SP versus ISTp was conducted in 5,354 primi- or secundigravidae in four West African countries with a low prevalence of resistance to SP (The Gambia, Mali, Burkina Faso and Ghana). Women in the IPTp-SP group received SP on two or three occasions whilst women in the ISTp group were screened two or three times with a RDT and treated if positive for malaria with artemether-lumefantrine (AL). ISTp-AL was non-inferior to IPTp-SP in preventing low birth weight (LBW), anemia and placental malaria, the primary trial endpoints. The prevalence of LBW was 15.1% and 15.6% in the IPTp-SP and ISTp-AL groups respectively (OR = 1.03 [95% CI: 0.88, 1.22]). The mean hemoglobin concentration at the last clinic attendance before delivery was 10.97g/dL and 10.94g/dL in the IPTp-SP and ISTp-AL groups respectively (mean difference: -0.03 g/dL [95% CI: -0.13, +0.06]). Active malaria infection of the placenta was found in 24.5% and in 24.2% of women in the IPTp-SP and ISTp-AL groups respectively (OR = 0.95 [95% CI 0.81, 1.12]). More women in the ISTp-AL than in the IPTp-SP group presented with malaria parasitemia between routine antenatal clinics (310 vs 182 episodes, rate difference: 49.4 per 1,000 pregnancies [95% CI 30.5, 68.3], but the number of hospital admissions for malaria was similar in the two groups.

**Conclusions:**

Despite low levels of resistance to SP in the study areas, ISTp-AL performed as well as IPTp-SP. In the absence of an effective alternative medication to SP for IPTp, ISTp-AL is a potential alternative to IPTp in areas where SP resistance is high. It may also have a role in areas where malaria transmission is low and for the prevention of malaria in HIV positive women receiving cotrimoxazole prophylaxis in whom SP is contraindicated.

**Trial Registration:**

ClinicalTrials.gov NCT01084213

Pan African Clinical trials Registry PACT201202000272122

## Introduction

Malaria infection during pregnancy (MIP) is a threat to both the pregnant woman and her fetus. Currently recommended approaches for the control of MIP are provision of effective treatment, insecticide treated bednets and intermittent preventive treatment with sulfadoxine-pyrimethamine (IPTp-SP) given at each antenatal clinic (ANC) attendance after the first trimester [1]. IPTp-SP is effective at preventing maternal anemia and low birthweight (LBW) in areas where *Plasmodium falciparum* is susceptible to SP [2–5] but uptake of the intervention is low in many communities [6] and its efficacy is threatened by the emergence of high level resistance of *P*. *falciparum* to SP in some parts of Africa. There is evidence from Malawi, Uganda, the Democratic Republic of The Congo and Tanzania that IPTp-SP is losing its efficacy in preventing the adverse effects of MIP [7–11] and it has been reported that in an area of north eastern Tanzania where *P*. *falciparum* strains frequently carry a 581G *dhps* mutation in addition to the quintuple *dhfr/dhps* SP resistance mutations, administration of IPTp-SP increases placental parasitisation, inflammation in the placenta, fetal anemia and severe malaria in the infant [12–14]. Mefloquine and the combination of azithromycin and chloroquine have been investigated as alternatives to SP for IPTp but both were too poorly tolerated to be recommended for this purpose [15,16]. There are currently no alternative drugs recommended for use in IPTp.

An alternative approach to IPTp-SP is screening women with a rapid diagnostic test (RDT) during routine antenatal clinic (ANC) attendances and treating those who are positive with an effective antimalarial combination, an approach termed intermittent screening and treatment in pregnancy (ISTp). An initial trial of ISTp in Ghana showed that ISTp with artemether lumefantrine (ISTp-AL) was not inferior to IPTp-SP in preventing LBW and maternal anemia but its impact on placental malaria was not investigated in this study [17]. Here, we describe the results of a larger, non-inferiority trial undertaken to investigate whether ISTp-AL is non-inferior to IPTp-SP in preventing malaria infection of the placenta as well as being non-inferior in the prevention of low birth weight (LBW) and anemia.

## Methods

### Study sites

The study was undertaken at sites in Burkina Faso, Ghana, Mali and The Gambia, where malaria transmission is moderately high or high and seasonal. Resistance of *P*. *falciparum* to SP is currently low in all four countries [18] (S1 Fig and S1 Table)

### Ethics and registration

The trial protocol and amendments were approved by the ethics committees of each of the participating African centers and by the ethics committee of the London School of Hygiene & Tropical Medicine (S2 Table). A Data and Safety Monitoring Board (DSMB) reviewed the overall conduct of the trial, which was monitored by independent clinical monitors, and approved the analytical plans.

#### Trial registration

ClinicalTrials.gov (NCT01084213); Pan African Clinical trials Registry (PACT201202000272122).

### Recruitment procedure and randomization

Community sensitization and training of ANC and project staff preceded the start of the trial. Primi- or secundigravidae who attended a study ANC between 31^st^ May 2010 and 31^st^ October 2011 were eligible to join the trial if they were between 16 and 30 weeks of gestation (assessed by measurement of the symphysis-fundal height), permanent residents of the study area and attending the ANC clinic for the first time. Exclusion criteria were a severe chronic infection, including clinical AIDS, known allergy to SP, prior receipt of SP during the pregnancy or an intention to leave the study area prior to delivery. HIV screening was conducted at first ANC attendance in accordance with local practice and women who were HIV positive were referred for further investigation and follow-up and were not recruited into the trial. Informed, written consent was sought from all women who met the eligibility criteria. Women who consented to join the trial were randomized using a pre-defined randomization procedure prepared in Stata version 11 (StataCorp, College Station, Texas) by an independent statistician, employing permuted blocks of ten. Clinic staff who recruited women to the study were blind to the results of the randomization process. The study was open-label but investigators, clinic staff recording birth weight, laboratory staff and the project statistician were blind to treatment allocation. Women were provided with a long-lasting insecticide treated bed net (Permanet 2, Westergaard Fransen, Copenhagen) at their first ANC attendance and prescribed daily doses of ferrous sulfate (200mg) and folic acid (0.4mg) for the duration of their pregnancy.

### Interventions and follow-up procedures

At the initial ANC visit, a blood sample was obtained for preparation of a blood film and filter paper blood spot and for determination of the hemoglobin (Hb) concentration. Women in the IPTp-SP arm then received their first treatment with SP (1500mg sulfadoxine/75mg pyrimethamine)(UNICEF, Copenhagen) under direct observation whilst women in the ISTp-AL arm were screened with a RDT and treated with artemether-lumefantrine (AL)(UNICEF, Copenhagen) for three days if positive. The first dose of AL was given under observation but subsequent doses were taken at home. A similar procedure was followed at the second and third routine ANC visits, scheduled to occur at intervals of 4 to 6 weeks depending on the gestational age of the woman at ANC booking. In three countries, two doses of SP were given whilst in Ghana three doses were given in line with national guidelines. Women in the IPTp-SP group who had observed fever and any symptoms suggestive of malaria were screened with an RDT and treated with AL but not given SP on that occasion. At the fourth ANC visit, scheduled to occur between 36–40 weeks of gestation, Hb concentration was measured and blood films were taken. Women in either group who attended an ANC outside a scheduled visit with symptoms judged by a routine member of the clinic staff to be suggestive of malaria were screened with a RDT and treated with AL if positive. Adverse events were monitored passively throughout the study period. Women were encouraged to deliver at a health facility where a peripheral blood sample was collected. Following delivery, a placental smear and biopsy were obtained and the birth weight of the baby was measured. Miscarriages, still births, neonatal deaths and congenital abnormalities were recorded. Women who delivered at home were visited as soon as possible after delivery and the baby weighed. If birth weight was not recorded within seven days of delivery, this was not included in the according to protocol (ATP) analysis. Study women and their babies were visited at home six weeks post partum for clinical assessment.

### Laboratory methods

Blood films were read initially by two microscopists; discrepant films were read by a third microscopist and a consensus result obtained using a standardized algorithm [19] as described in S1 Text. Supplementary Methods. The First Response P. *falciparum* HRP2/pLDH RDT (Premier Medical Corporation Ltd., Mumbai, India) was used at each site throughout the study. Hemoglobin concentrations were measured using 301 Hemocue analysers (HemoCue, Anglom, Sweden). The methods used to obtain and process placental biopsies and to record histological findings are described in the supplement. Blood spots obtained from study women who were positive for *P*. *falciparum* at presentation were tested for mutations associated with resistance to SP using a pooled sequencing approach described in the supplement [20].

### Data management and statistical analysis

TeleForm (Version 10.4.1, Cardiff Software Inc., Vista CA), was used for electronic data capture. Following verification, scanned information was uploaded to a local database and also to an anonymized database held at the London School of Hygiene & Tropical Medicine. Further checks and cleaning were carried out by a central data management team who did not take part in implementing the study. Three primary outcomes were defined: risk of LBW, maternal Hb concentration prior to delivery and the prevalence of placental malaria. Sample size was calculated to give 90% power to exclude a clinically important difference in birth weight between study groups, as described in the supplement. This resulted in a target sample size of 5000 women (1250 per center). Defining a non-inferiority margin *a priori* for LBW and placental malaria (PM) was complicated by the fact the exact risk of these outcomes by center was not known prior to the start of the trial; the odds ratio (OR) was used as a practical solution to this problem [21]. For LBW, we specified an OR based on a consensus among the investigators that a 4% risk difference in the prevalence of LBW would be clinically acceptable if the prevalence was 20% in the IPTp-SP group i.e. the OR should be less than 1.263 (equating to a risk difference of 3.25% if the prevalence was 15%, and a 2.3% difference if the prevalence was 10%). For PM, the margin was specified as a 5% excess of active malaria infection in the ISTp-AL group, assuming a 25% prevalence of PM in the IPTp group (specified in terms of an OR less than 1.286). The non-inferiority margin for hemoglobin concentration at the final follow-up visit before delivery was specified to exclude a reduction of 0.2 g/dL in Hb concentration in the ISTp-AL group relative to the IPTp group.

Two analysis plans were prepared covering clinical and laboratory findings respectively (S4 and S5 Texts) and both were approved by the DSMB which allowed the clinical findings to be reviewed before all blood films and histological slides had been read. Stata version 13 (StataCorp, College Station, Texas) was used for all analyses. Both according to protocol (ATP) and modified intention to treat (ITT) analyses were performed in concordance with the CONSORT guidelines for non-inferiority trials [22]. All women who had been randomized and contributed information on the outcome under consideration were included in the ITT analysis, except that multiple births, still births and miscarriages were excluded from the analyses of birth weight. Inclusion in the ATP analysis required that a woman had either received SP or been tested with an RDT on at least two occasions and contributed information on the endpoint under consideration. For the primary outcomes, non-inferiority was formally investigated by calculating the OR (for risk of LBW and placental malaria), or the difference in means (for Hb concentration and birth weight), with adjustment for study centre as a covariate. Two-sided 90, 95 and 99 per cent confidence intervals were calculated and compared to the pre-specified non-inferiority margins (details are provided in the supplement). One-sided p-values were used to assess evidence against the null hypothesis that ISTp-AL is inferior by the pre-specified margin [23]. For secondary outcomes, 95% confidence intervals for differences between groups were calculated but non-inferiority margins for these outcomes were not specified *a priori*. Rate differences for incidence of illness episodes were calculated using the method of Xu et al [24].

The protocol, amendments and analyses plans are provided in the supplementary materials (S2, S3, S4 and S5 Texts).

## Results

### Study population

**Fig 1:**
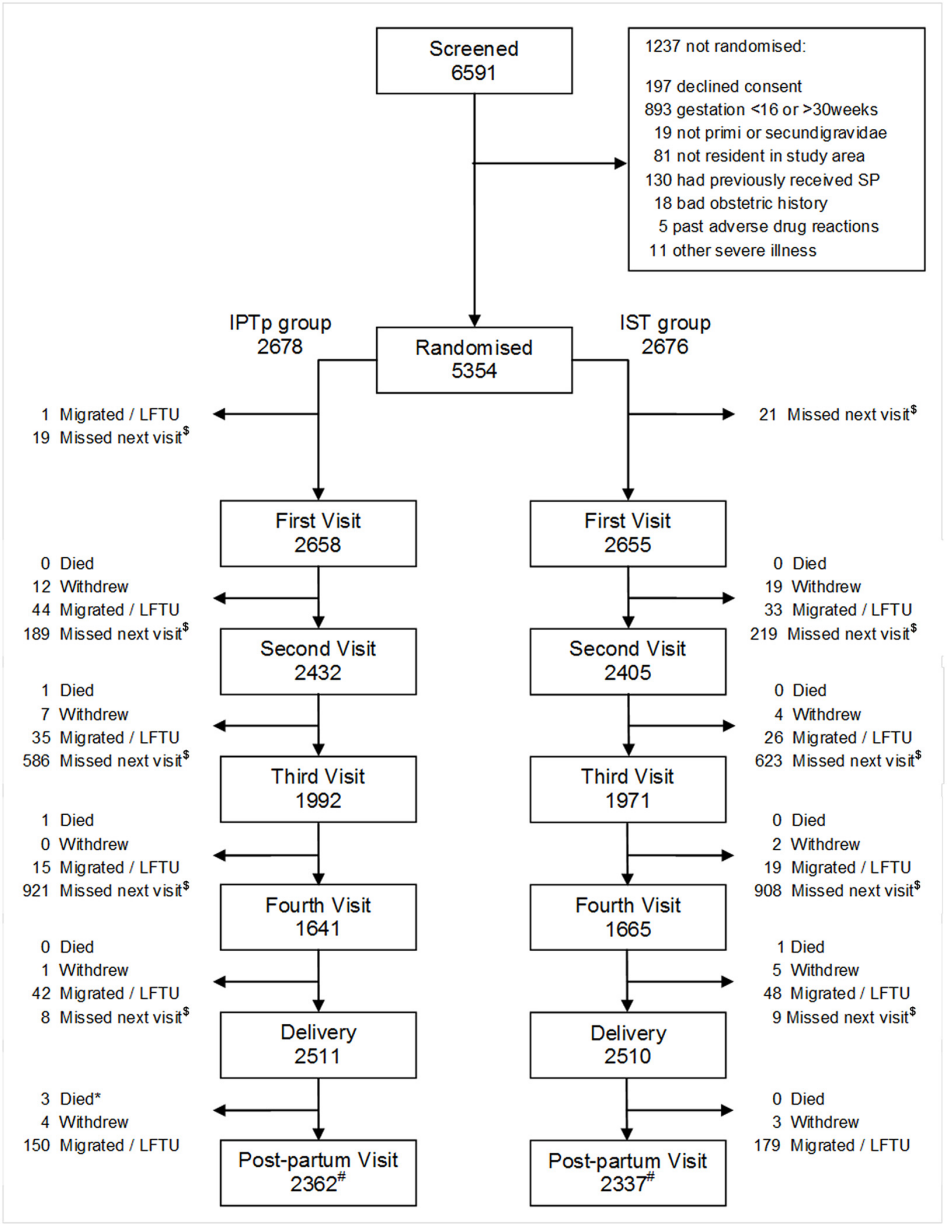
CONSORT Flow Diagram. Footnote: LTFU—Lost to follow-up. * two of these 3 deaths occurred on the day of delivery. ^$^ missed subsequent visit but remained in follow up. ^#^ numbers shown at post-partum include 8 and 9 women in IPTp-SP and ISTp-AL groups respectively who were not seen at delivery but who remained in follow-up.

The overall trial profile is shown in Fig 1 and profiles by center in S2, S3, S4 and S5 Figs; 6591 primi- or secundigravidae were screened for eligibility, 5354 of whom (81%) were enrolled and randomly assigned to one of the two intervention groups. Baseline characteristics were well matched overall (Table 1) and by center (S3 Table). Based on symphysis-fundal height, women were enrolled at a mean of 20.6 weeks of gestation (median 20 weeks) of gestation; 97.5% of women were enrolled before 28 weeks of gestation. Fifty-two percent of women in the ISTp-AL group tested positive with an RDT and were treated with AL at some stage of their pregnancy, 35.6% once, 12.7% twice and 3.4% more than twice. The number of women who delivered at home was similar in the two groups (7.43% and 8.82% in the IPTp-SP and ISTp-AL groups respectively). Placental samples were obtained from 91.7% of the 4591 women who delivered in a health centre. Women included in the ATP population for estimation of birth weight were generally similar to those not included (S4 Table).

**Table 1:** Baseline characteristics of the study participants.

		IPTp-SP		ISTp-AL		Total	
**Age group**	Mean (SD)	20.4	(3.21)	20.4	(3.43)	20.4	(3.32)
	Median (IQR)	20	(18, 22)	20	(18, 22)	20	(18, 22)
		**No**.	**%**	**No**.	**%**	**No**.	**%**
**Gravidity**	primi-	1450	54.4	1476	55.4	2926	54.9
	secundi-	1214	45.6	1189	44.6	2403	45.1
**Education**	None	1213	45.6	1210	45.7	2423	45.6
	Basic	1104	41.5	1065	40.2	2169	40.8
	Secondary	304	11.4	337	12.7	641	12.1
	Tertiary	42	1.58	36	1.36	78	1.47
**Religion**	Christian	752	28.2	777	29.3	1529	28.8
	Islam	1849	69.3	1823	68.7	3672	69.0
	Traditional	41	1.54	31	1.17	72	1.35
	none/other	25	0.94	21	0.79	46	0.86
**Marital Status**	Married	2433	91.4	2408	90.9	4841	91.2
	Not married	228	8.57	242	9.13	470	8.85
**Slept under treated net last night**	yes	1555	58.8	1544	58.8	3099	58.8
	no	1090	41.2	1084	41.3	2174	41.2
**IRS in sleeping room**	yes	135	5.38	123	4.95	258	5.17
**in last 6 months**	no	2373	94.6	2364	95.1	4737	94.8
**Malaria parasitemia**							
Positive (by microscopy)		808/2609	30.97	807/2628	30.71	1615/5237	30.84
Geometric mean density		1345.9		1297.8		1321.6	
(95% CI)		(1222.9, 1481.2)		(1179.0, 1428.6)		(1235.0, 1414.2)	
**Hemoglobin at first visit**	<5	5	0.19	4	0.15	9	0.17
	5–7.99	167	6.24	174	6.51	341	6.38
	8–10.99	1566	58.5	1546	57.9	3112	58.2
	11+	937	35.0	947	35.5	1884	35.2
	Mean (SD)	10.31	(1.51)	10.34	(1.55)	10.32	(1.53)

CI; Confidence interval; IRS, indoor residual spraying of insecticide; IQR, inter-quartile range; SD, standard deviation.

### Birth weight

**Fig 2:**
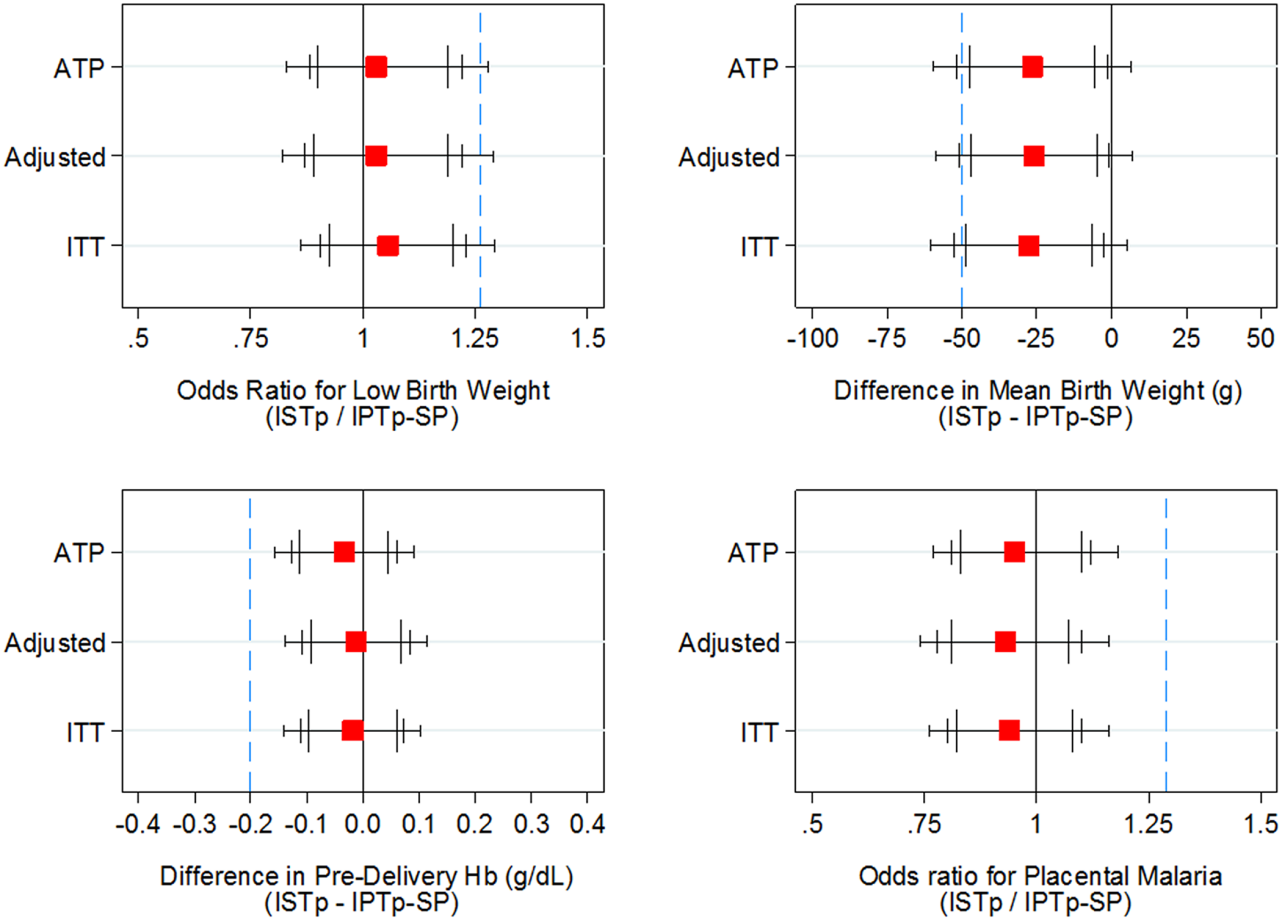
Non-inferiority plots for primary outcomes and mean birth weight. The figures show two-sided 90%, 95% and 99% confidence intervals (largest to smallest vertical bars, respectively), equivalent to one-sided 95%, 97.5% and 99.5% confidence intervals. The dashed blue vertical line indicates the non-inferiority margin. ATP, according to protocol population (adjusted for site); adjusted, ATP population adjusted for site, gravidity, age group, gestational age, ITN use and socio-economic status; ITT, intention to treat, (adjusted for site). Numbers included in the analyses and numeric values of the estimates are provided in the supplement (S4 Table).

Birth weight was measured within 7 days of delivery for 4,659 of the 5,354 women enrolled (87%), 4,391 of whom were included in the ATP analysis. Risk of LBW was 15.1% and 15.6% in the IPTp-SP and ISTp-AL groups respectively (Table 2). Unadjusted and adjusted ORs for LBW in the ATP population were 1.03 (95% CI: 0.88, 1.22), and 1.03 (0.87, 1.22) respectively (Fig 2) (S5 Table). The ORs for the 4738 women included in the ITT analysis were very similar: 1.05 (95%CI: 0.90, 1.23) (S5 Table). The 95% confidence intervals for these ORs all exclude the pre-specified non-inferiority margin of 1.263, with one-sided p-values assessing the null hypothesis of inferiority of ≤0.01. Analysis by country showed a similar pattern of non-inferiority in prevention of LBW (S6 Table).

**Table 2:** Risk of low birth weight and anemia by intervention group.

	Burkina		Gambia		Ghana		Mali		Overall	
	IPTp-SP	ISTp-AL	IPTp-SP	ISTp-AL	IPTp-SP	ISTp-AL	IPTp-SP	ISTp-AL	IPTp-SP	ISTp-AL
**Number of women**	599	613	484	461	524	552	564	573	2183	2208
**Low birth weight**										
No. Low birth weight	108	108	72	60	86	97	64	79	330	344
% low birth weight	17.9	17.5	14.9	13.0	16.4	17.4	11.4	13.8	15.1	15.6
Odds Ratio (95% CI)	0.98 (0.73, 1.32)		0.86 (0.59, 1.24)		1.08 (0.79, 1.49)		1.25 (0.88, 1.78)		1.03 (0.88, 1.22)	
**Anemia**										
***Number assessed at fourth visit***	506	546	324	318	256	272	448	464	1534	1600
No. with Hb < 11 g / dL	246	270	202	179	135	149	176	205	759	803
% Hb < 11 g / dL	48.6	49.5	62.4	56.3	52.7	54.8	39.3	44.2	49.5	50.2
Odds Ratio (95% CI)	1.03 (0.81, 1.32)		0.78 (0.57, 1.07)		1.09 (0.77, 1.53)		1.22 (0.94, 1.59)		1.03 (0.90, 1.19)	
No. with Hb < 8 g / dL	10	13	13	14	2	5	1	5	26	37
% Hb < 8 g / dL	1.98	2.38	4.01	4.40	0.78	1.84	0.22	1.08	1.69	2.31
Odds Ratio (95% CI)	1.21 (0.53, 2.78)		1.10 (0.51, 2.38)		2.38 (0.46, 12.4)		4.87 (0.57, 41.8)		1.39 (0.84, 2.32)	
***Number assessed at delivery***	490	523	280	272	217	239	437	450	2170	2220
No. with Hb < 11 g / dL	177	203	116	120	91	100	95	112	801	863
% Hb < 11 g / dL	36.1	38.8	41.4	44.1	41.9	41.8	21.7	24.9	36.9	38.9
Odds Ratio (95% CI)	1.12 (0.87, 1.45)		1.12 (0.80, 1.56)		1.00 (0.69, 1.45)		1.19 (0.87, 1.63)		1.11 (0.95, 1.30)	
No. with Hb < 8 g / dL	7	11	4	8	0	1	2	1	33	41
% Hb < 8 g / dL	1.43	2.10	1.43	2.94	0	0.42	0.46	0.22	1.52	1.85
Odds Ratio (95% CI)	1.48 (0.57, 3.86)		2.09 (0.62, 7.03)		-		0.48 (0.044, 5.36)		1.58 (0.79, 3.18)	

CI; Confidence interval. Numbers shown are for the according to protocol (ATP) population. The odds ratio is given as the measure of effect because the non-inferiority margin for the OR (1.263 for low birth weight) can be defined independently of the underlying prevalence. The pooled estimate of the odds ratio is also not affected by changes in prevalence in the different sites as the risk difference or risk ratio would be. Outcomes for low birth weight split by gravidity are given in S5 Table.

**Fig 3:**
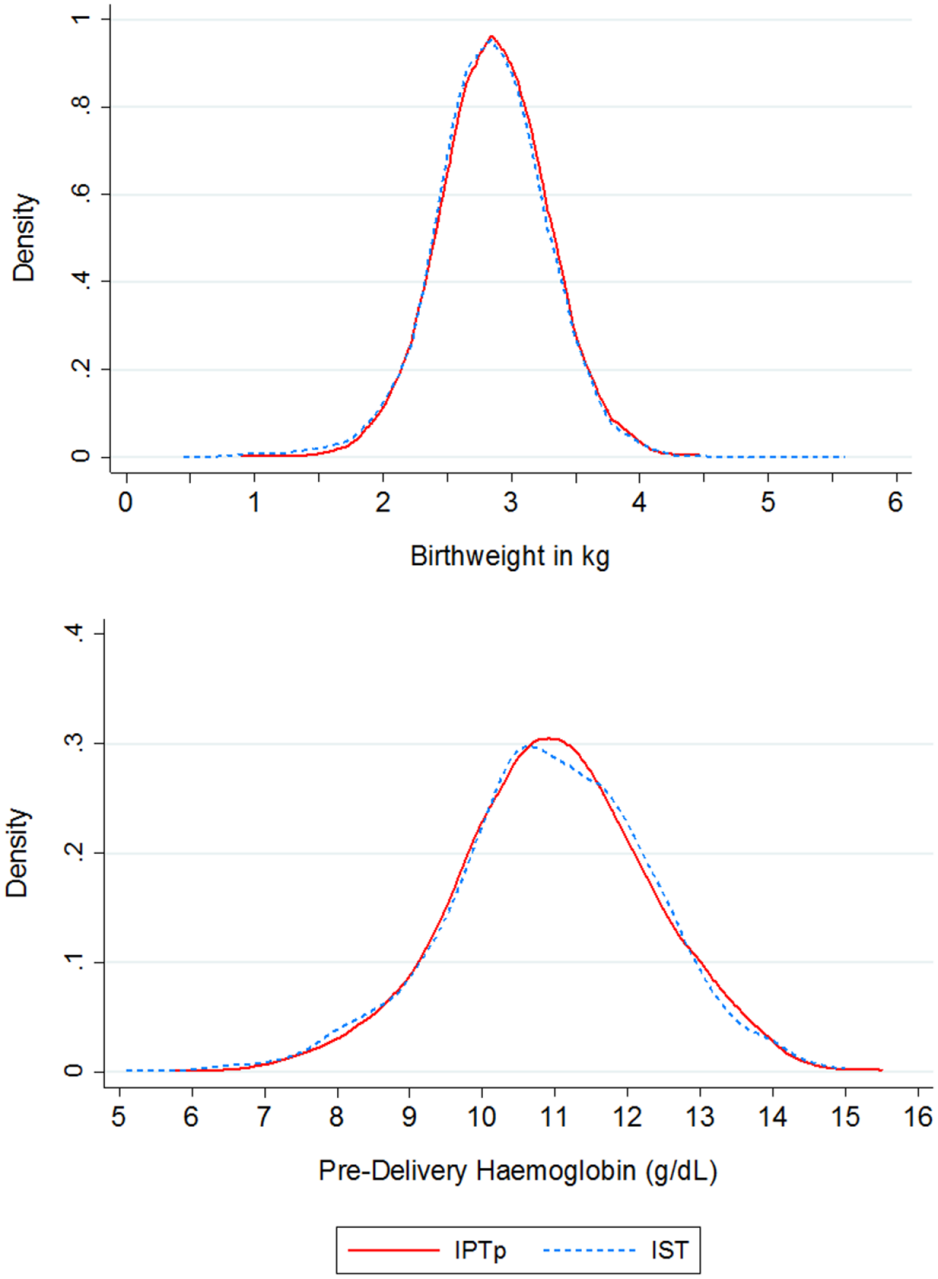
Distribution of birth weight (A) and hemoglobin concentration (B) at fourth ANC visit by intervention group. Birth weight distributions were estimated using data from 2183 women in the IPTp-SP group and from 2208 women in the ISTp-AL group. Hemoglobin distributions utilise data from 1534 women in the IPTp-SP group and from 1600 women in the ISTp-AL group.

Mean birth weight was 2,866 g (SD 418 g) and 2,838 g (SD 438 g) in the IPTp-SP and ISTp-AL groups respectively. The distribution of birth weights was very similar between the intervention groups both overall (Fig 3) and by country (S6 Fig). The mean differences in birth weight between study groups (ISTp-AL—IPTp-SP) were: unadjusted ATP, -26.6 g (95% CI: -51.8, -1.5); adjusted ATP, -26.0 g (95% CI: -51.1, -0.9); ITT, -27.7 g (95% CI: -52.8, -2.6). The two sided 95% confidence interval (equivalent to a one-sided 97.5% CI) overlaps the noninferiority margin for both the ATP and adjusted ATP analyses. However, the two-sided 90% CI (equivalent to a one-sided 95% CI) excluded the non-inferiority margin of 50g in all three sets of analyses (Fig 2). One-sided p-values, indicating moderate evidence against the null hypothesis of inferiority were 0.034, 0.031 and 0.041 for ATP, adjusted ATP and ITT analyses respectively. The difference in mean birth weight between IPTp-SP and ISTp-AL groups in Ghana, where IPTp-SP and ISTp-AL were both delivered on three occasions, was very small and similar to that seen in other countries (S7 Fig).

### Hemoglobin concentration

Mean Hb concentration at the last clinic attendance before delivery was 10.97 g/dL (SD 1.35 g/dL) and 10.94 g/L (SD 1.38 g/dL) in women in the IPTp-SP or ISTp-AL groups respectively. The distribution of Hb concentrations was very similar in the two groups overall (Fig 3) and at each site (S8 Fig). The 95% confidence intervals for the mean difference in Hb concentrations for both unadjusted and adjusted ATP, and ITT analyses excluded the non-inferiority margin of -0.2g/dL: unadjusted ATP, -0.03g/dL (95% CI: -0.13, 0.06); adjusted ATP, -0.01g/dL (95% CI: -0.11, 0.08); ITT, -0.02g/dL (95% CI: -0.11, 0.07). The 99% confidence intervals also excluded this non-inferiority margin (Fig 2). The prevalence of anemia (Hb < 11.0 g/dL) and severe anemia (Hb < 5 g/dL) at the final follow-up visit before delivery and at delivery were similar in each study group (Table 2), with the confidence interval for the OR overlapping one in all cases.

### Placental malaria

Interpretable placental biopsy specimens were obtained from 71.6% and 70.6% of women in the IPTp-SP or ISTp-AL groups respectively. The characteristics of women from whom a placental sample was obtained and of those from whom it was not because they had delivered at home were very similar (S7 Table). The prevalence of active malaria infection of the placenta was very similar in the IPTp-SP and ISTp-AL groups overall: 24.5% and 24.2% respectively (OR 0.95 [95% CI 0.81, 1.12]) (Table 3). ISTp-AL was thus non-inferior to IPTp-SP in preventing active placental malaria in all analyses and at all levels of confidence (Fig 2). Acute infections were slightly more frequent in the IPTp-SP group than in the ISTp-AL group whilst the opposite trend was observed for chronic infections but neither of these differences was statistically significant (Table 3). Moderate or abundant pigment in fibrin was observed more frequently in samples from women in the ISTp-AL than in the IPTp-SP group (Table 3) but differences between groups were small (P = 0.076). Intervillous inflammation of varying degrees of severity was seen in a similar proportion of samples from women in each group. Placental blood smears were positive in 128/1,815 (7.05%) of women in the IPTp-SP group and in 157/1,858 (8.45%) of women in the ISTp-AL group (OR 1.22 [95% CI: 0.95, 1.55], p = 0.11). Although the prevalence of malaria infection of the placenta varied by country, results obtained in the IPTp-SP and ISTp-AL groups were similar in each country (S8 Table) and by gravidity (S9 Table).

**Table 3:** Prevalence of placental malaria by study group.

	IPTp		ISTp-AL		
	N	%	N	%	Odds ratio (95% CI)
**Placental histology**	N = 1672		N = 1690		
Active infection (acute or chronic)^1^	409	24.5	409	24.2	0.95 (0.81, 1.12)
**Infection type**	N = 1672		N = 1689		
Acute infections	207	12.4	177	10.5	0.81 (0.65, 1.00)
Chronic infections	202	12.1	231	13.7	1.13 (0.91, 1.39)
**Placental blood smear**	N = 1815		N = 1858		
Negative	1687	93.0	1701	91.6	
Positive	128	7.05	157	8.45	1.22 (0.95, 1.55)
**Intervillous inflammation**	N = 1685		N = 1704		
<5 per high powered field	1449	86.0	1432	84.0	0.87 (0.72, 1.05)
5–10	186	11.0	214	12.6	1.15 (0.93, 1.42)
10–25	38	2.26	38	2.23	0.96 (0.61, 1.52)
>25	12	0.71	20	1.17	1.62 (0.79, 3.32)
**Among those without active infection**					
**Pigment in fibrin** ^2^	N = 1262		N = 1276		
None	441	34.9	387	30.3	0.82 (0.68, 0.99)
Mild	365	28.9	379	29.7	1.02 (0.86, 1.22)
Moderate	447	35.4	498	39.0	1.15 (0.96, 1.37)
Abundant	9	0.71	12	0.94	1.29 (0.54, 3.09)
**Pigment in macrophages** ^2^	N = 1259		N = 1277		
None	1096	87.1	1045	81.8	0.68 (0.54, 0.85)
Mild	42	3.34	59	4.62	1.37 (0.91, 2.06)
Moderate	114	9.05	163	12.8	1.44 (1.11, 1.87)
Abundant	7	0.56	10	0.78	1.38 (0.52, 3.64)
**Pigment in fibrin and macrophages** ^2^	N = 1260		N = 1277		
None	1098	87.1	1046	81.9	0.68 (0.54, 0.85)
Mild	41	3.25	58	4.54	1.38 (0.92, 2.08)
Moderate	114	9.05	163	12.8	1.44 (1.11, 1.87)
Abundant	7	0.56	10	0.78	1.38 (0.52, 3.64)

Numbers shown for the according to protocol (ATP) population.

^1^ Co-primary outcome for study. Odds ratios are adjusted for site, Acute infection: Infected maternal erythrocytes and no or minimal pigment. Chronic infection: Infected maternal erythrocytes and moderate or abundant pigment.

^2^ Data for presence of pigment are shown only for children without active malaria infection. Numbers available for each analysis are shown, as complete information on malaria infection, inflammation and pigment in different locations was not available for all samples: seven women were missing data on pigment in fibrin, 28 on pigment in macrophages, and six on inflammation.

### Secondary outcomes

Unscheduled clinic visits between routine ANC contacts were more frequent among women in the ISTp-AL group (1204 visits) than in those in the IPTp-SP group (988 visits) (p = 0.001). Common complaints were headache (50.3% of visits), fever (32.1%), abdominal pain (27.1%) and waist pain (14.6%); headache and chills were reported more frequently by women in the ISTp-AL than in those in the IPTp-SP group. A RDT was done due to suspicion that a woman might have malaria more frequently in the ISTp-AL group (977 occasions) than in the IPTp-SP group (680 occasions) and the test was positive on 439 (44.9%) and 235 (34.6%) occasions respectively. The incidence at unscheduled visits of malaria parasitemia, confirmed by microscopy, in the two groups was 124.9 (95% CI 111.4, 139.7) and 75.2 (95% CI: 64.7, 87.0) per 1000 pregnancies, a rate difference 49.4 per 1000 pregnancies (95% CI 30.5, 68.3)(p<0.001). Geometric mean parasite density was similar in the IPTp-SP and ISTp-AL groups (9741.6 and 11302.0/μl per μl respectively) (p = 0.459). Nearly all malaria infections were mild (95.2% and 93.4% and in the ISTp-AL and IPTp-SP groups respectively). Only 22 women with a positive blood slide were admitted to hospital, 13 in the ISTp-AL and 9 in the IPTp-SP groups respectively.

The prevalence of malaria parasitemia in the IPTp-SP and ISTp-AL groups was very similar at the final ANC visit (89/1487 [5.99%] vs 106/1569 [6.76%]), at delivery (171/2030 [8.42%] vs 206/2104 [9.79%]) and post-partum (135/2065 [6.54%] vs 137/2110 [6.49%]) giving ORs of 1.13 (95% CI: 0.84, 1.52), 1.18 (95% CI 0.95, 1.47) and 0.98 (95% CI 0.77, 1.26), respectively. Geometric mean parasite densities/μl in the IPTp-SP and ISTp-AL groups were also similar at each of these contacts: 1303.4 vs. 1872.6, p = 0.161; 2750.5 vs. 3398.5, p = 0.359 and 380.4 vs. 524.5, p = 0.091 at final ANC, delivery and post-partum, respectively.

Parasites obtained at enrolment were genotyped for markers of SP resistance. The *dhps* K540**E** mutation, which predicts the *dhfr-dhps* quintuple mutant haplotype that confers a high risk of SP failure, [25] was absent in Burkina Faso, Ghana, Gambia, and San, Mali, and present at < 1% in Kita, Mali (S10 Table). Each site harbored moderate frequencies of *dhfr* mutations, but neither the *dhfr* I164**L** nor *dhps* A581**G** mutations were detected. These findings are supported by a parallel study in two of the study sites (Burkina Faso and Mali) which showed a high level of clinical efficacy of SP in asymptomatic, pregnant women [26].

Six women died during the study, five in the IPTp-SP group and one in the ISTp-AL group. Deaths in the IPTp-SP group were attributed to postpartum haemorrhage (2), severe malarial anemia (1), septicemia (1) and eclampsia (1). The only death in the ISTp-AL group was attributed to placenta praevia. The number of miscarriages, pre-term births, still births, babies small for gestational age and perinatal deaths were similar in each intervention group (Table 4). Thirty-three congenital abnormalities were detected, 15 in the IPTp-SP group and 18 in the ISTp-AL group (S11 Table).

**Table 4:** Adverse birth outcomes and deaths.

	IPTp		IST		Odds Ratio (95% CI)
**Adverse birth outcomes**	No.	%	No.	%	
Congenital abnormality^1^	15	0.60	18	0.72	1.20 (0.60, 2.39)
Small for gestational age	392	23.2	432	25.3	1.12 (0.95, 1.31)
Miscarriage	15	0.60	18	0.72	1.19 (0.60, 2.37)
Preterm birth	173	7.05	177	7.20	1.04 (0.83, 1.29)
Stillbirths	76	3.06	84	3.39	1.12 (0.82, 1.53)
**Deaths**					
Perinatal death	115	4.62	122	4.92	1.07 (0.82, 1.39)
Maternal death	5	0.19	1	0.04	0.20 (0.02, 1.71)

CI; Confidence interval.

^1^ Details of the congenital abnormalities are given in the supplement, S10 Table. Small for gestational age was defined as birth weight less than the 10th centile of the nomogram defined by Landis et al.[28]

No drug related serious adverse event was recorded. Women in the IPTp-SP complained of dizziness, sleeplessness, weakness, nausea and vomiting since their last ANC visit more frequently than women in the ISTp-AL group and associated this with taking SP.

## Discussion

Intermittent screening and treatment was non-inferior to IPTp-SP in preventing LBW, maternal anemia and placental malaria, the primary trial end-points, in four countries where *P*. *falciparum* is still sensitive to SP and IPTp-SP is still likely to be highly effective. However, the incidence of clinic visits between routine ANC attendances with symptoms accompanied by malaria parasitemia was higher in women in the ISTp-AL than in the IPTp-SP group. Nearly all these infections were mild, with very few hospital admissions in either group, but the significance of this finding needs further evaluation.

Strengths of this trial are that it was large and powered to exclude a modest difference in LBW, anemia or placental malaria and that similar findings were recorded at each site, which differed in their intensity of malaria transmission, strengthening the general application of the study’s findings. Limitations of the study include lack of a complete set of observations for all women, although completeness was similar between intervention groups. At the time at which this study was conducted, the WHO recommended that pregnant women should receive at least two treatments with SP during pregnancy and, in three of the study countries (Burkina Faso, Mali and The Gambia), national practice was to give only two doses. In these countries some women in the ISTp-AL group were screened for malaria during a third routine ANC visit whilst, following national policy, those in the IPTp-SP group did not receive a third dose of IPTp-SP. However, in Ghana, where SP administration and RDT screening were each conducted on three occasions, similar results were obtained to those at the other sites. This suggests that this limitation has not affected the overall results of the trial.

It is possible that RDTs were used more readily in women from the ISTp-AL than in those from the IPTp-SP group who presented between routine clinics, although we have no evidence that this was the case. If this was so, this could have contributed to the higher frequency with which parasitaemia was detected at unscheduled visits among women in the ISTp-AL group. Testing more women between routine visits might also benefit the outcomes of pregnancy if it resulted in additional infections being detected. The potential impact of introducing ISTp on overall practice in the antenatal clinic is an important issue and one which is currently being investigated.

Intermittent screening and treatment was well accepted by pregnant women and clinic staff, as noted previously [27]. However, ISTp, whether with AL or other regimens, is likely to be more costly than IPTp-SP because the cost of an RDT is higher than the cost of a treatment with SP, and more complex to administer. These drawbacks need to be balanced against the non-financial benefits achieved from sparing a large number of women unnecessary administration of a drug during pregnancy and the mild side effects associated with taking SP. Detailed information on the costs, cost-effectiveness and acceptability of ISTp-AL during this trial will be reported subsequently.

Under what conditions might ISTp prove to be a valuable approach to the control of malaria in pregnancy? Firstly, there are currently no grounds to suggest that it should replace IPTp-SP in areas where *P*. *falciparum* remains sensitive to SP and every effort needs to be made to increase coverage with IPTp-SP in such areas. However, in areas of eastern and southern Africa where *P*. *falciparum* has become highly resistant to SP, ISTp-AL may be superior to IPTp-SP; this is being investigated in on-going trials in Kenya and Malawi. This study has shown that ISTp-AL is a potential future option for control of malaria in pregnancy in West Africa if resistance to SP continues to increase in this part of the continent, as seems likely. Secondly, the level of malaria transmission below which IPTp-SP is no longer useful is not known and, consequently, there is a reluctance to stop IPTp-SP in low transmission settings without an alternative. In such situations, both within and outside sub-Saharan Africa, ISTp-AL could be an effective alternative until malaria is no longer a significant threat. In addition, routine screening at ANC clinics would provide valuable, local information on changes in the prevalence of malaria associated with environmental changes or control activities. A third potential use of ISTp-AL is in HIV infected pregnant women receiving cotrimoxazole prophylaxis, in whom IPTp-SP is contraindicated. Finally, ISTp-AL could be used in the first trimester of pregnancy when IPTp-SP is not recommended. The results of this trial show that ISTp-AL is a potentially valuable approach to the control of malaria in pregnancy in some circumstances but more research is needed to determine its place among the limited number of options available to control malaria in pregnancy.

## Supporting Information

S1 Fig Map of West Africa showing location of the study centres. (DOCX)

S2 Fig Consort charts by centre—Burkina Faso. (DOCX)

S3 Fig Consort charts by centre—The Gambia. (DOCX)

S4 Fig Consort charts by centre—Ghana. (DOCX)

S5 Fig Consort charts by centre—Mali. (DOCX)

S6 Fig Distribution curves for birth weight by centre. (DOCX)

S7 Fig Non-inferiority plots for Ghana and the other 3 sites combined. (DOCX)

S8 Fig Distribution of haemoglobin concentration at fourth ANC visit by centre. (DOCX)

S1 Table Characteristics of the study sites. (DOCX)

S2 Table Ethical approval. (DOCX)

S3 Table Baseline characteristics of study participants by study centre. (DOCX)

S4 Table Comparison of baseline characteristics for women. (DOCX)

S5 Table Point estimates and confidence intervals for the primary outcomes. (DOCX)

S6 Table Risk of low birth weight by intervention group, gravidity and country. (DOCX)

S7 Table Characteristics of women with or without a placental histology sample. (DOCX)

S8 Table Placental malaria findings by intervention group and country. (DOCX)

S9 Table Placental malaria findings by intervention group and gravidity. (DOCX)

S10 Table Relative frequencies of molecular markers of SP resistance. (DOCX)

S11 Table Details of congenital abnormalities by study group. (DOCX)

S1 Text Supplementary Methods. (DOCX)

S2 Text Trial Protocol. (DOCX)

S3 Text Amendments to Trial Protocol. (DOCX)

S4 Text Analysis Plan-Clinical Findings. (DOCX)

S5 Text Analysis Plan- Laboratory Findings. (DOCX)

S6 Text CONSORT Extension for Non-inferiority and Equivalence Trials Checklist. (DOCX)
