# Supplementary figures and images for: A Non-Inferiority, Individually Randomized Trial of Intermittent Screening and Treatment versus Intermittent Preventive Treatment in the Control of Malaria in Pregnancy
Source: PLoS One. 2015 Aug 10;10(8):e0132247. doi: 10.1371/journal.pone.0132247 (PMC4530893; doi:10.1371/journal.pone.0132247)

**S1 Fig.**

Map of West Africa showing location of the study centres.


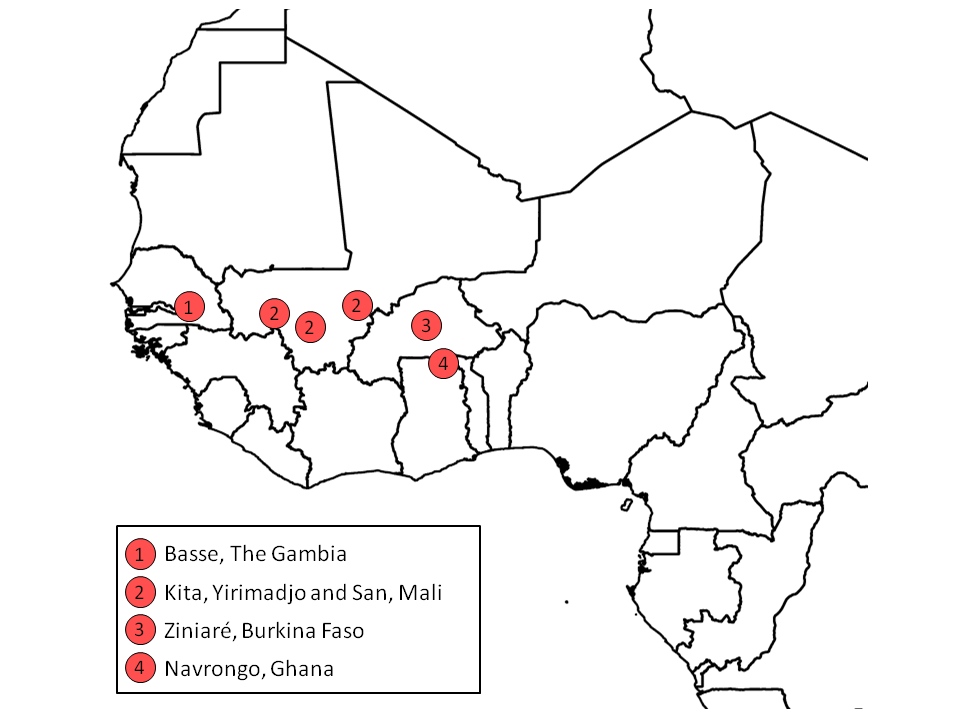

Supplement: S1 Fig — (DOCX) [file pone.0132247.s001.docx]
