# Supplementary material for: A Non-Inferiority, Individually Randomized Trial of Intermittent Screening and Treatment versus Intermittent Preventive Treatment in the Control of Malaria in Pregnancy
Source: PLoS One. 2015 Aug 10;10(8):e0132247. doi: 10.1371/journal.pone.0132247 (PMC4530893; doi:10.1371/journal.pone.0132247)
Supplement: S2 Fig — (DOCX) [file pone.0132247.s002.docx]

**S2 Fig.**

Consort charts by centre - Burkina Faso.

First Visit

720

Second Visit

674

Third Visit

587

Fourth Visit

536

Delivery

686

Post-partum Visit

659

First Visit

718

Second Visit

664

Third Visit

597

Fourth Visit

562

Delivery

694

Post-partum Visit

658

Screened

1610

Randomised

1438

IPTp group

720

IST group

718

0 Died

2 Withdrew

11 Migrated / LFTU

33 Missed next visit^$^

1 Died

0 Withdrew

8 Migrated / LFTU

111 Missed next visit^$^

1 Died

0 Withdrew

3 Migrated / LFTU

158 Missed next visit^$^

0 Died

0 Withdrew

8 Migrated / LFTU

0 Missed next visit^$^

0 Died

1 Withdrew

26 Migrated / LFTU

0 Died

2 Withdrew

5 Migrated / LFTU

47 Missed next visit^$^

0 Died

0 Withdrew

4 Migrated / LFTU

110 Missed next visit^$^

0 Died

0 Withdrew

6 Migrated / LFTU

139 Missed next visit^$^

0 Died

1 Withdrew

5 Migrated / LFTU

1 Missed next visit^$^

0 Died

0 Withdrew

37 Migrated / LFTU

0 Migrated / LFTU

0 Missed next visit^$^

0 Missed next visit^$^

Not randomised: 172

20 declined consent

142 gestation <16 or >30 weeks

1 not primi or secundigravidae

2 not resident in study area

7 had previously received SP

1 bad obstetric history

1 past adverse drug reactions

3 other severe illness

LTFU, lost to follow-up. ^$^ missed subsequent visit but remained in follow up.
