# Supplementary material for: A Non-Inferiority, Individually Randomized Trial of Intermittent Screening and Treatment versus Intermittent Preventive Treatment in the Control of Malaria in Pregnancy
Source: PLoS One. 2015 Aug 10;10(8):e0132247. doi: 10.1371/journal.pone.0132247 (PMC4530893; doi:10.1371/journal.pone.0132247)
Supplement: S3 Fig — (DOCX) [file pone.0132247.s003.docx]

**S3 Fig.**

Consort charts by centre - The Gambia.

First Visit

624

Second Visit

561

Third Visit

399

Fourth Visit

353

Delivery

561

Post-partum Visit

489

First Visit

624

Second Visit

545

Third Visit

371

Fourth Visit

343

Delivery

550

Post-partum Visit

472

Screened

1796

Randomised

1250

IPTp group

625

IST group

625

0 Died

2 Withdrew

12 Migrated / LFTU

50 Missed next visit^$^

0 Died

1 Withdrew

3 Migrated / LFTU

198 Missed next visit^$^

0 Died

0 Withdrew

5 Migrated / LFTU

239 Missed next visit^$^

0 Died

0 Withdrew

24 Migrated / LFTU

7 Missed next visit^$^

2 Died*

0 Withdrew

77 Migrated / LFTU

0 Died

9 Withdrew

9 Migrated / LFTU

62 Missed next visit^$^

0 Died

0 Withdrew

9 Migrated / LFTU

227 Missed next visit^$^

0 Died

0 Withdrew

11 Migrated / LFTU

244 Missed next visit^$^

0 Died

1 Withdrew

30 Migrated / LFTU

6 Missed next visit^$^

0 Died

1 Withdrew

83 Migrated / LFTU

1 Missed next visit^$^

1 Missed next visit^$^

Not randomised: 546

49 declined consent

482 gestation <16 or >30 weeks

7 not primi or secundigravidae

13 not resident in study area

5 had previously received SP

6 bad obstetric history

1 past adverse drug reactions

1 other severe illness

LTFU, lost to follow-up. * 1 of these 2 deaths occurred on the day of delivery. ^$^ missed subsequent visit but remained in follow up.
