# Supplementary material for: A Non-Inferiority, Individually Randomized Trial of Intermittent Screening and Treatment versus Intermittent Preventive Treatment in the Control of Malaria in Pregnancy
Source: PLoS One. 2015 Aug 10;10(8):e0132247. doi: 10.1371/journal.pone.0132247 (PMC4530893; doi:10.1371/journal.pone.0132247)
Supplement: S4 Fig — (DOCX) [file pone.0132247.s004.docx]

**S4 Fig.**

Consort charts by centre – Ghana.

First Visit

653

Second Visit

554

Third Visit

430

Fourth Visit

271

Delivery

624

Post-partum Visit

599

First Visit

652

Second Visit

558

Third Visit

443

Fourth Visit

283

Delivery

638

Post-partum Visit

605

Screened

1380

Randomised

1306

IPTp group

653

IST group

653

0 Died

1 Withdrew

12 Migrated / LFTU

86 Missed next visit^$^

0 Died

3 Withdrew

7 Migrated / LFTU

200 Missed next visit^$^

0 Died

0 Withdrew

2 Migrated / LFTU

357 Missed next visit^$^

0 Died

0 Withdrew

4 Migrated / LFTU

0 Missed next visit^$^

0 Died

0 Withdrew

25 Migrated / LFTU

0 Died

1 Withdrew

6 Migrated / LFTU

88 Missed next visit^$^

0 Died

0 Withdrew

4 Migrated / LFTU

199 Missed next visit^$^

0 Died

0 Withdrew

1 Migrated / LFTU

358 Missed next visit^$^

0 Died

0 Withdrew

3 Migrated / LFTU

0 Missed next visit^$^

0 Died

1 Withdrew

32 Migrated / LFTU

0 Missed next visit^$^

1 Missed next visit^$^

Not randomised: 74

13 declined consent

20 gestation <16 or >30 weeks

5 not primi or secundigravidae

16 not resident in study area

30 had previously received SP

1 bad obstetric history

0 past adverse drug reactions

5 other severe illness

LTFU, lost to follow-up. ^$^ missed subsequent visit but remained in follow up.
