# Supplementary material for: A Non-Inferiority, Individually Randomized Trial of Intermittent Screening and Treatment versus Intermittent Preventive Treatment in the Control of Malaria in Pregnancy
Source: PLoS One. 2015 Aug 10;10(8):e0132247. doi: 10.1371/journal.pone.0132247 (PMC4530893; doi:10.1371/journal.pone.0132247)
Supplement: S5 Fig — (DOCX) [file pone.0132247.s005.docx]

**S5 Fig.**

Consort charts by centre – Mali.

First Visit

661

Second Visit

643

Third Visit

576

Fourth Visit

481

Delivery

640

Post-partum Visit

615

First Visit

661

Second Visit

638

Third Visit

560

Fourth Visit

477

Delivery

628

Post-partum Visit

602

Screened

1805

Randomised

1360

IPTp group

680

IST group

680

0 Died

7 Withdrew

9 Migrated / LFTU

20 Missed next visit^$^

0 Died

3 Withdrew

7 Migrated / LFTU

77 Missed next visit^$^

0 Died

0 Withdrew

5 Migrated / LFTU

167 Missed next visit^$^

0 Died

1 Withdrew

6 Migrated / LFTU

1 Missed next visit^$^

1 Died*

3 Withdrew

22 Migrated / LFTU

0 Died

7 Withdrew

13 Migrated / LFTU

22 Missed next visit^$^

0 Died

4 Withdrew

9 Migrated / LFTU

87 Missed next visit^$^

0 Died

2 Withdrew

1 Migrated / LFTU

167 Missed next visit^$^

1 Died

3 Withdrew

10 Migrated / LFTU

2 Missed next visit^$^

0 Died

1 Withdrew

27 Migrated / LFTU

1 Migrated / LFTU

18 Missed next visit^$^

19 Missed next visit^$^

Not randomised: 445

115 declined consent

249 gestation <16 or >30 weeks

6 not primi or secundigravidae

50 not resident in study area

88 had previously received SP

6 bad obstetric history

3 past adverse drug reactions

2 other severe illness

LTFU, lost to follow-up. There were two deaths, both occurred at delivery: 1 in the IPTp arm from haemorrhage at delivery and one from placenta Previa in the IST arm. * This death occurred on the day of delivery. ^$^ missed subsequent visit but remained in follow up.
