# Supplementary material for: A Non-Inferiority, Individually Randomized Trial of Intermittent Screening and Treatment versus Intermittent Preventive Treatment in the Control of Malaria in Pregnancy
Source: PLoS One. 2015 Aug 10;10(8):e0132247. doi: 10.1371/journal.pone.0132247 (PMC4530893; doi:10.1371/journal.pone.0132247)
Supplement: S6 Fig — (DOCX) [file pone.0132247.s006.docx]

## S6 Fig.

Distribution curves for birth weight by centre.

Numbers used to estimate the birth weight distributions are as follows. Burkina Faso 611 IPTp-SP, and 620 ISTp-AL, The Gambia 484 IPTp-SP, 461 ISTp-AL; Ghana 524 IPTp-SP, 554 ISTp-AL; Mali 564 IPTp-SP, 573 ISTp-AL.
