# Supplementary material for: A Non-Inferiority, Individually Randomized Trial of Intermittent Screening and Treatment versus Intermittent Preventive Treatment in the Control of Malaria in Pregnancy
Source: PLoS One. 2015 Aug 10;10(8):e0132247. doi: 10.1371/journal.pone.0132247 (PMC4530893; doi:10.1371/journal.pone.0132247)
Supplement: S7 Fig — (DOCX) [file pone.0132247.s007.docx]

**S7 Fig.**

Non-inferiority plots for Ghana and the other 3 sites combined.

Because IPTp-SP was given on three occasions in Ghana but only on two in the other study countries a comparative analysis of findings in Ghana and the other three study countries has been undertaken.

Figures show two-sided 90%, 95% and 99% confidence intervals (largest to smallest vertical bars, respectively), equivalent to one-sided 95%, 97.5% and 99.5% confidence intervals. The dashed blue vertical line indicates the non-inferiority margin. ^*^ Numbers included are as follows. LBW and birth weight: Ghana 524 IPTp-SP, 554 ISTp-AL; other sites 1659 IPTp-SP, 1654 ISTp-AL. Haemoglobin: Ghana 256 IPTp-SP, 272 ISTp-AL; other sites 1278 IPTp-SP, 1328 ISTp-AL. Placental malaria: Ghana 387 IPTp-SP, 399 ISTp-AL; other sites 1285 IPTp-SP, 1291 ISTp-AL.
