# Supplementary material for: A Non-Inferiority, Individually Randomized Trial of Intermittent Screening and Treatment versus Intermittent Preventive Treatment in the Control of Malaria in Pregnancy
Source: PLoS One. 2015 Aug 10;10(8):e0132247. doi: 10.1371/journal.pone.0132247 (PMC4530893; doi:10.1371/journal.pone.0132247)
Supplement: S8 Fig — (DOCX) [file pone.0132247.s008.docx]

**S8 Fig.**

Distribution of haemoglobin concentration at fourth ANC visit by centre.

Numbers used to estimate the haemoglobin distributions are as follows. Burkina Faso 506 IPTp-SP, and 546 ISTp-AL, The Gambia 324 IPTp-SP, 318 ISTp-AL; Ghana 256 IPTp-SP, 272 ISTp-AL; Mali 448 IPTp-SP, 464 ISTp-AL.
