# Supplementary material for: A Non-Inferiority, Individually Randomized Trial of Intermittent Screening and Treatment versus Intermittent Preventive Treatment in the Control of Malaria in Pregnancy
Source: PLoS One. 2015 Aug 10;10(8):e0132247. doi: 10.1371/journal.pone.0132247 (PMC4530893; doi:10.1371/journal.pone.0132247)
Supplement: S1 Table — (DOCX) [file pone.0132247.s009.docx]

## S1 Table

Characteristics of the study sites.

|  | **Burkina Faso** | **Ghana** | **Mali** | **The Gambia** |
| --- | --- | --- | --- | --- |
| Centre | Faculty of Health Sciences, University of Ouagadougou | Navrongo Health Research Centre, Navrongo | Malaria Research and Training Centre, Bamako | MRC Laboratories, Basse |
| Total population | 261,892 | 153,000 | 118,000 | 120,000 |
| Number of ANC clinics | 7 | 15 | 6 | 2 |
| Routine HIV screening | VCT | VCT | VCT | VCT |
| HIV prevalence* | 1.6% | 2.8% | 1.3% | 2.8% |
| Predominant malaria parasite | *Plasmodium falciparum* | *Plasmodium falciparum* | *Plasmodium falciparum* | *Plasmodium falciparum* |
| Estimated entomological inoculation rate (infectious bites/person/year)(EIR) | 100 – 300 | 200 | 19 – 21 during the rainy season | 10 – 50 |
| Seasonality of malaria | Marked seasonality | Marked seasonality | Marked seasonality | Marked seasonality |
| Routine first line treatment | First trimester: Quinine.  Second / Third trimester: AQ/AS; AL. | First trimester: Quinine.  Second / Third trimester: AQ/AS; AL. | First trimester: Quinine.  Second / Third trimester: AQ/AS; AL. | First trimester: Quinine.  Second / Third trimester: AQ/AS; AL. |

* UNAIDS. HIV and AIDS estimates. 2012. <http://www.unaids.org/en/regionscountries/countries/> (accessed 13th May 2014).

(VCT = Voluintary Counselling and Testing; AQ/AS = amodiaquine/artesunate; AL = artemether/lumefantine).
