# Supplementary material for: A Non-Inferiority, Individually Randomized Trial of Intermittent Screening and Treatment versus Intermittent Preventive Treatment in the Control of Malaria in Pregnancy
Source: PLoS One. 2015 Aug 10;10(8):e0132247. doi: 10.1371/journal.pone.0132247 (PMC4530893; doi:10.1371/journal.pone.0132247)
Supplement: S2 Table — (DOCX) [file pone.0132247.s010.docx]

## S2 Table

Ethical approval.

The initial trial protocol and subsequent amendments were approved by the ethics committees listed below.

Burkina Faso: Comité national d’ethique pour la recherché en santé (CNERS) Ministère de la Santé, BP

7009, Ouagadougou 03, Burkina Faso.

Ghana : Ethics committee of the Ghana Health Service Committee Research & Development

Division, Ghana Health Science, P O Box 190,Accra, Ghana.

Navrongo Health Research Centre Institutional Review Board, Navrongo Health Research

Centre, P. O. Box 114, Navrongo, Ghana.

Mali: Ethics committee of the Ministere de l’Enseignement Superieur et de la Recherche

Scientifique Universite de Bamako-BP 1805- Bamako, Mali.

The Gambia: The Gambia Government /MRC laboratories Joint Ethics Committee, C/O MRC

Laboratories, Fajara, PO Box 273 Banjul, The Gambia.

UK: The ethics committee of the London School of Hygiene & Tropical Medicine, Keppel St.

London WC1 7HT, UK.
