# Supplementary material for: A Non-Inferiority, Individually Randomized Trial of Intermittent Screening and Treatment versus Intermittent Preventive Treatment in the Control of Malaria in Pregnancy
Source: PLoS One. 2015 Aug 10;10(8):e0132247. doi: 10.1371/journal.pone.0132247 (PMC4530893; doi:10.1371/journal.pone.0132247)
Supplement: S3 Table — (DOCX) [file pone.0132247.s011.docx]

## S3 Table

Baseline characteristics of study participants – by study centre.

|  |  | Burkina | | Gambia | | Ghana | | Mali | |
| --- | --- | --- | --- | --- | --- | --- | --- | --- | --- |
|  |  |  |  |  |  |  |  |  |  |
| **Age** | Mean (SD) | 20.6 (2.80) | | 20.1 (3.16) | | 21.6 (3.67) | | 19.4 (3.27) | |
| Median (IQR) | | 20 (18, 22) | | 20 (18, 22) | | 21 (19,24) | | 19 (17, 21) | |
|  |  |  |  |  |  |  |  |  |  |
|  |  | **No.** | **%** | **No.** | **%** | **No.** | **%** | **No.** | **%** |
| **Gravidity** | primi | 665 | 47.0 | 709 | 56.7 | 727 | 55.8 | 825 | 60.7 |
|  | secundi | 751 | 53.0 | 541 | 43.3 | 576 | 44.2 | 535 | 39.3 |
|  |  |  |  |  |  |  |  |  |  |
| **Socioeconomic** | least poor | 39 | 2.9 | 250 | 21.5 | 162 | 12.4 | 580 | 43.9 |
| **status** | less poor | 61 | 4.5 | 295 | 25.3 | 167 | 12.8 | 507 | 38.4 |
|  | middle | 326 | 23.9 | 271 | 23.3 | 278 | 21.4 | 158 | 12.0 |
|  | more poor | 386 | 28.3 | 138 | 11.9 | 453 | 34.8 | 56 | 4.2 |
|  | most poor | 552 | 40.5 | 210 | 18.0 | 242 | 18.6 | 20 | 1.5 |
|  |  |  |  |  |  |  |  |  |  |
| **Education** | None | 1076 | 75.3 | 645 | 53.0 | 266 | 20.4 | 436 | 32.1 |
|  | Basic | 212 | 14.8 | 447 | 36.7 | 849 | 65.0 | 661 | 48.7 |
|  | Secondary | 136 | 9.5 | 124 | 10.2 | 144 | 11.0 | 237 | 17.5 |
|  | Tertiary | 5 | 0.3 | 2 | 0.2 | 47 | 3.6 | 24 | 1.8 |
|  |  |  |  |  |  |  |  |  |  |
| **Religion** | Christian | 317 | 22.1 | 6 | 0.5 | 1128 | 86.4 | 78 | 5.7 |
|  | Islam | 1115 | 77.7 | 1213 | 99.5 | 94 | 7.2 | 1250 | 92.0 |
|  | Traditional | 1 | 0.1 | 0 | 0 | 68 | 5.2 | 3 | 0.2 |
|  | none/ other | 2 | 0.1 | 0 | 0 | 16 | 1.2 | 28 | 2.1 |
|  |  |  |  |  |  |  |  |  |  |
| **Marital Status** | Married | 1356 | 94.8 | 1198 | 98.5 | 1136 | 87.0 | 1151 | 84.8 |
|  | Not married | 75 | 5.2 | 18 | 1.5 | 171 | 13.0 | 207 | 15.2 |
|  |  |  |  |  |  |  |  |  |  |
| **Used treated** | yes | 771 | 54.2 | 1021 | 84.3 | 489 | 37.5 | 818 | 61.3 |
| **net last night** | no | 652 | 45.8 | 190 | 15.7 | 816 | 62.5 | 516 | 38.7 |
|  |  |  |  |  |  |  |  |  |  |
| **IRS in sleeping** | yes | 0 | 0 | 135 | 13.3 | 115 | 9.0 | 8 | 0.6 |
| **room in last 6 months** | no | 1365 | 100 | 879 | 86.7 | 1156 | 91.0 | 1337 | 99.4 |
|  |  |  |  |  |  |  |  |  |  |
| **Home delivery** | | 10 | 0.73 | 239 | 21.6 | 153 | 13.2 | 4 | 0.32 |
| **Malaria parasitemia** | |  |  |  |  |  |  |  |  |
| Positive (by microscopy) | | 602 | 42.0 | 105 | 8.79 | 624 | 48.0 | 284 | 21.7 |
| Geometric mean density | |  | 1054.3 |  | 398.4 |  | 1950.0 |  | 1343.5 |
| (95% CI) | | 951.5, 1168.7 | | 307.0, 517.2 | | 1760.8, 2159.6 | | 1131.4, 1595.3 | |
|  |  |  |  |  |  |  |  |  |  |
| **Hemoglobin at fist visit** | <5 | 1 | 0.07 | 3 | 0.24 | 2 | 0.15 | 3 | 0.22 |
|  | 5-7.99 | 100 | 6.96 | 68 | 5.44 | 115 | 8.81 | 58 | 4.28 |
|  | 8-10.99 | 827 | 57.6 | 700 | 56.0 | 844 | 64.6 | 741 | 54.7 |
|  | 11+ | 508 | 35.4 | 478 | 38.3 | 345 | 26.4 | 553 | 40.8 |
|  |  |  | |  | |  | |  | |
|  | Mean (SD) | 10.4 (2.92) | | 10.4 (1.47) | | 9.98 (1.52) | | 10.6 (2.61) | |
| Median (IQR) | | 10.4 (9.4, 11.3) | | 10.5 (9.5, 11.4) | | 10 (9,11) | | 10.7 (9.7, 11.5) | |

CI, confidence interval; IRS, indoor residual spraying of insecticide; IQR, inter-quartile range; SD, standard deviation.
