# Supplementary material for: A Non-Inferiority, Individually Randomized Trial of Intermittent Screening and Treatment versus Intermittent Preventive Treatment in the Control of Malaria in Pregnancy
Source: PLoS One. 2015 Aug 10;10(8):e0132247. doi: 10.1371/journal.pone.0132247 (PMC4530893; doi:10.1371/journal.pone.0132247)
Supplement: S4 Table — (DOCX) [file pone.0132247.s012.docx]

## S4 Table

Comparison of baseline characteristics for women included in the ATP analysis for birth weight, and those excluded.

|  |  | **Included in ATP (N=4391)** | | **Excluded from ATP (N=963)** | |
| --- | --- | --- | --- | --- | --- |
| **Age** | mean (SD) | 20.4 | (3.30) | 20.3 | (3.43) |
|  | Median (IQR) | 20 | (18, 22) | 20 | (18, 22) |
|  |  |  |  |  |  |
|  |  | **No** | **%** | **No** | **%** |
| **Gravidity** | Primi- | 2395 | 54.8 | 531 | 55.4 |
|  | Secundi- | 1975 | 45.2 | 428 | 44.6 |
|  |  |  |  |  |  |
| **Socio-economic status** | least poor | 850 | 20.1 | 181 | 19.6 |
|  | less poor | 850 | 20.1 | 180 | 19.5 |
|  | middle | 845 | 20.0 | 188 | 20.4 |
|  | more poor | 855 | 20.2 | 178 | 19.3 |
|  | most poor | 828 | 19.6 | 196 | 21.2 |
|  |  |  |  |  |  |
| **Education** | None | 1978 | 45.3 | 445 | 47.0 |
|  | Basic | 1768 | 40.5 | 401 | 42.4 |
|  | Secondary | 551 | 12.6 | 90 | 9.5 |
|  | Tertiary | 68 | 1.6 | 10 | 1.1 |
|  |  |  |  |  |  |
| **Religion** | Christian | 1276 | 29.2 | 253 | 26.7 |
|  | Islam | 3001 | 68.7 | 671 | 70.8 |
|  | Traditional | 58 | 1.3 | 14 | 1.5 |
|  | none/other | 36 | 0.8 | 10 | 1.1 |
|  |  |  |  |  |  |
| **Marital status** | married | 3976 | 91.1 | 865 | 91.2 |
|  | not married | 387 | 8.9 | 83 | 8.7 |
|  |  |  |  |  |  |
| **Slept under treated** | yes | 2535 | 58.4 | 564 | 60.3 |
| **net last night** | no | 1803 | 41.6 | 371 | 39.7 |
|  |  |  |  |  |  |
| **IRS in sleeping room** | yes | 210 | 5.1 | 48 | 5.4 |
| **in last 6 months** | no | 3901 | 94.9 | 836 | 94.6 |
|  |  |  |  |  |  |
| **Malaria parasitemia** | |  |  |  |  |
| Positive by microscopy | | 1328 | 30.9 | 287 | 30.7 |
| Geometric mean density | | 1357.7 |  | 1166.0 |  |
|  |  |  | |  | |
| **Hemoglobin** | <5 | 7 | 0.16 | 2 | 0.21 |
| **at first visit** | 5-7.99 | 268 | 6.11 | 73 | 7.58 |
|  | 8-10.99 | 2571 | 58.66 | 541 | 56.18 |
|  | 11+ | 1537 | 35.07 | 347 | 36.03 |
|  | Mean (SD) | 10.3 | (2.27) | 10.3 | (1.63) |

CI, confidence interval; IRS, indoor residual spraying of insecticide; IQR, inter-quartile range; SD, standard deviation
