# Supplementary material for: A Non-Inferiority, Individually Randomized Trial of Intermittent Screening and Treatment versus Intermittent Preventive Treatment in the Control of Malaria in Pregnancy
Source: PLoS One. 2015 Aug 10;10(8):e0132247. doi: 10.1371/journal.pone.0132247 (PMC4530893; doi:10.1371/journal.pone.0132247)
Supplement: S5 Table — (DOCX) [file pone.0132247.s013.docx]

## S5 Table

Point estimates and confidence intervals for the primary outcomes upon which the graphics presented in Fig. 2 are based.

| **Low birth weight** | **N**  **(IPTp-SP)** | **N**  **(ISTp-AL)** | **Odds ratio**  **(95% CI)** | **90% CI** | **99% CI** | **p-value^$^** | **Risk difference**  **(95% CI)** | **90% CI** | **99% CI** | **p-value^$^** |
| --- | --- | --- | --- | --- | --- | --- | --- | --- | --- | --- |
| According to protocol | 2183 | 2208 | 1.03 (0.88, 1.22) | 0.90, 1.19 | 0.83, 1.28 | 0.008 | 0.43 % (-1.70%, 2.56%) | -1.36%, 2.22% | -2.37%, 3.23% | 0.0047 |
| ATP, adjusted* | 2098 | 2089 | 1.03 (0.87, 1.22) | 0.89, 1.19 | 0.82, 1.29 | 0.010 | 0.43 % (-1.72%, 2.58%) | -1.38%, 2.23% | -2.40%, 3.25% | 0.0051 |
| Intention to treat | 2376 | 2362 | 1.05 (0.90, 1.23) | 0.93, 1.20 | 0.86, 1.29 | 0.011 | 0.73 % (-1.37%, 2.83%) | -1.04%, 2.49% | -2.03%, 3.49% | 0.0093 |
|  |  |  |  |  |  |  |  |  |  |  |
| **Placental malaria** |  |  | **Odds ratio**  **(95% CI)** | **90% CI** | **99% CI** | **p-value^$^** | **Risk difference**  **(95% CI)** | **90% CI** | **99% CI** | **p-value^$^** |
| According to protocol | 1672 | 1690 | 0.95 (0.81, 1.12) | 0.83, 1.10 | 0.77, 1.18 | <0.001 | -0.79% (-3.57%, 1.99%) | -3.12%, 1.54% | -4.44%, 2.86% | <0.0001 |
| ATP, adjusted* | 1606 | 1601 | 0.93 (0.78, 1.10) | 0.81, 1.07 | 0.74, 1.16 | <0.0001 | -1.22% (-4.07%, 1.63%) | -3.61%, 1.17% | -4.96%, 2.52% | <0.0001 |
| Intention to treat | 1798 | 1773 | 0.94 (0.80, 1.10) | 0.82, 1.08 | 0.76, 1.16 | <0.0001 | -1.02% (-3.72%, 1.69%) | -3.29%, 1.26% | -4.57%, 2.54% | <0.0001 |
|  |  |  |  |  |  |  |  |  |  |  |
| **Pre-delivery hemoglobin** |  |  | **Mean difference (95% CI)** | **90% CI** | **99% CI** | **p-value^$^** |  |  |  |  |
| According to protocol | 1534 | 1600 | -0.03 (-0.13, 0.06) | -0.11, 0.04 | -0.16, 0.09 | <0.001 |  |  |  |  |
| ATP, adjusted* | 1469 | 1509 | -0.01 (-0.11, 0.08) | -0.09, 0.07 | -0.14, 0.11 | <0.0001 |  |  |  |  |
| Intention to treat | 1610 | 1636 | -0.02 (-0.11, 0.07) | -0.10, 0.06 | -0.14, 0.10 | <0.0001 |  |  |  |  |
|  |  |  |  |  |  |  |  |  |  |  |
| **Mean birth weight** |  |  | **Mean difference (95% CI)** | **90% CI** | **99% CI** | **p-value^$^** |  |  |  |  |
| According to protocol | 2183 | 2208 | -26.6 (-51.8, -1.48) | -47.7, -5.52 | -59.7, 6.43 | 0.034 |  |  |  |  |
| ATP, adjusted* | 2098 | 2089 | -25.9 (-51.1, -0.85) | -47.1, -4.89 | -59.0, 7.06 | 0.031 |  |  |  |  |
| Intention to treat | 2376 | 2362 | -27.7 (-52.8, -2.62) | -48.8, -6.66 | -60.7, 5.27 | 0.041 |  |  |  |  |

ATP, according to protocol; CI, confidence interval * adjusted: ATP population adjusted for site, gravidity, age group, gestational age, ITN use and socio-economic status. Risk differences are estimated using the modified least-squares method of Cheung et al.^1^ Risk differences should be interpreted with caution since the pooled estimate of the risk difference may give unequal weight to sites with higher prevalence (since the risk can be further from unity but still within the non-inferiority margin where prevalence is higher). ^$^ P-values indicate support for the null hypothesis that ISTp-AL is worse than IPTp-SP by the pre-specified margin. Small p-values therefore indicate evidence against the null hypothesis (that ISTp-AL is worse by an important amount), i.e. that ISTp-AL is non-inferior to IPTp-SP.

**References**

1. Cheung YB. A modified least-squares regression approach to the estimation of risk difference. Am J Epidemiol 2007; **166**: 1337-44.
