# Supplementary material for: A Non-Inferiority, Individually Randomized Trial of Intermittent Screening and Treatment versus Intermittent Preventive Treatment in the Control of Malaria in Pregnancy
Source: PLoS One. 2015 Aug 10;10(8):e0132247. doi: 10.1371/journal.pone.0132247 (PMC4530893; doi:10.1371/journal.pone.0132247)
Supplement: S6 Table — (DOCX) [file pone.0132247.s014.docx]

## S6 Table

Risk of low birth weight by intervention group, gravidity and country.

|  | **Burkina** | | **Gambia** | | **Ghana** | | **Mali** | | **Overall** | |
| --- | --- | --- | --- | --- | --- | --- | --- | --- | --- | --- |
|  | **IPTp-SP** | **ISTp-AL** | **IPTp-SP** | **ISTp-AL** | **IPTp-SP** | **ISTp-AL** | **IPTp-SP** | **ISTp-AL** | **IPTp-SP** | **ISTp-AL** |
| **All women**, N | 599 | 613 | 484 | 461 | 524 | 552 | 564 | 573 | 2183 | 2208 |
| No. Low birth weight | 108 | 108 | 72 | 60 | 86 | 97 | 64 | 79 | 330 | 344 |
| % low birth weight | 17.9 | 17.5 | 14.9 | 13.0 | 16.4 | 17.4 | 11.4 | 13.8 | 15.1 | 15.6 |
| Odds Ratio (95% CI) | 0.98 (0.73, 1.32) | | 0.86 (0.59, 1.24) | | 1.08 (0.79, 1.49) | | 1.25 (0.88, 1.78) | | 1.03 (0.88, 1.22) | |
| **Primigravidae**, N | 262 | 297 | 286 | 266 | 293 | 308 | 332 | 351 | 1173 | 1222 |
| No. Low birth weight | 74 | 78 | 54 | 42 | 53 | 63 | 46 | 58 | 227 | 241 |
| % low birth weight | 28.2 | 26.3 | 18.9 | 15.8 | 18.1 | 20.5 | 13.9 | 16.5 | 19.4 | 19.7 |
| Odds Ratio (95% CI) | 0.90 (0.62, 1.31) | | 0.81 (0.52, 1.25) | | 1.16 (0.78, 1.75) | | 1.23 (0.81, 1.87) | | 1.01 (0.83, 1.24) | |
| **Secundigravidae**, N | 337 | 316 | 198 | 195 | 231 | 244 | 232 | 222 | 998 | 977 |
| No. Low birth weight | 33 | 29 | 18 | 18 | 33 | 33 | 18 | 21 | 102 | 101 |
| % low birth weight | 9.79 | 9.18 | 9.09 | 9.23 | 14.3 | 13.5 | 7.76 | 9.46 | 10.2 | 10.3 |
| Odds Ratio (95% CI) | 0.93 (0.55, 1.57) | | 1.02 (0.51, 2.02) | | 0.94 (0.56, 1.58) | | 1.24 (0.64, 2.40) | | 1.00 (0.75, 1.34) | |

CI, confidence interval. Numbers shown are for the according to protocol (ATP) population. The odds ratio is given as the measure of effect because the non-inferiority margin for the OR (1.263 for low birth weight) can be defined independently of the underlying prevalence. The pooled estimate of the OR is also not affected by changes in prevalence in the different sites as the risk difference or risk ratio would be.
