# Supplementary material for: A Non-Inferiority, Individually Randomized Trial of Intermittent Screening and Treatment versus Intermittent Preventive Treatment in the Control of Malaria in Pregnancy
Source: PLoS One. 2015 Aug 10;10(8):e0132247. doi: 10.1371/journal.pone.0132247 (PMC4530893; doi:10.1371/journal.pone.0132247)
Supplement: S7 Table — (DOCX) [file pone.0132247.s015.docx]

## S7 Table

Characteristics of women with or without a placental histology sample.

|  |  | **No Placental**  **Sample** | | **Placental Sample Obtained** | | **Total** | |
| --- | --- | --- | --- | --- | --- | --- | --- |
| **Age** | mean (SD) | 20.5 | (3.44) | 20.4 | (3.27) |  |  |
|  | Median (IQR) | 20 | (18, 22) | 20 | (18, 22) |  |  |
|  |  |  |  |  |  |  |  |
|  |  | No. | % | No. | % | No. |  |
| **Gravidity** | Primi- | 951 | 53.6 | 1975 | 55.6 | 2926 | 54.9 |
|  | Secundi- | 824 | 46.4 | 1579 | 44.4 | 2403 | 45.1 |
|  |  |  |  |  |  |  |  |
| **Socio-economic status** | least poor | 320 | 18.7 | 711 | 20.7 | 1031 | 20.0 |
|  | less poor | 374 | 21.9 | 656 | 19.1 | 1030 | 20.0 |
|  | middle | 350 | 20.5 | 683 | 19.8 | 1033 | 20.1 |
|  | more poor | 327 | 19.1 | 706 | 20.5 | 1033 | 20.1 |
|  | most poor | 338 | 19.8 | 686 | 19.9 | 1024 | 19.9 |
|  |  |  |  |  |  |  |  |
| **Education** | None | 770 | 43.7 | 1653 | 46.6 | 2423 | 45.6 |
|  | Basic | 786 | 44.6 | 1383 | 39.0 | 2169 | 40.8 |
|  | Secondary | 184 | 10.4 | 457 | 12.9 | 641 | 12.1 |
|  | Tertiary | 22 | 1.2 | 56 | 1.6 | 78 | 1.5 |
|  |  |  |  |  |  |  |  |
| **Religion** | Christian | 457 | 25.9 | 1072 | 30.1 | 1529 | 28.7 |
|  | Islam | 1255 | 71.2 | 2417 | 68.0 | 3672 | 69.0 |
|  | Traditional | 37 | 2.1 | 35 | 1.0 | 72 | 1.4 |
|  | none/other | 13 | 0.7 | 33 | 0.9 | 46 | 0.9 |
|  |  |  |  |  |  |  |  |
| **Marital status** | married | 1628 | 92.4 | 3213 | 90.5 | 4841 | 91.2 |
|  | not married | 133 | 7.5 | 337 | 9.5 | 470 | 8.8 |
|  |  |  |  |  |  |  |  |
| **Slept under treated** | yes | 1115 | 64.0 | 1984 | 56.2 | 3099 | 58.8 |
| **net last night** | no | 628 | 36.0 | 1546 | 43.8 | 2174 | 41.2 |
|  |  |  |  |  |  |  |  |
| **IRS in sleeping room** | yes | 111 | 6.7 | 147 | 4.4 | 258 | 5.2 |
| **in last 6 months** | no | 1536 | 93.3 | 3201 | 95.6 | 4737 | 94.8 |
|  |  |  |  |  |  |  |  |
| **Malaria parasitemia** |  |  |  |  |  |  |  |
| Positive by microscopy | | 469 | 27.2 | 1146 | 32.7 | 1615 | 30.8 |
| Geometric mean density | |  | 1318.8 |  | 1322.7 |  | 1321.6 |
|  |  | (1156.7, 1503.8) | | (1222.1, 1431.6) | | (1235.0, 1414.2) | |
|  |  |  |  |  |  |  |  |
| **Hemoglobin** | <5 | 2 | 0.1 | 7 | 0.2 | 9 | 0.2 |
| **at first visit** | 5-7.99 | 115 | 6.5 | 226 | 6.3 | 341 | 6.4 |
|  | 8-10.99 | 1048 | 58.8 | 2064 | 57.9 | 3112 | 58.2 |
|  | 11+ | 618 | 34.7 | 1266 | 35.5 | 1884 | 35.2 |
|  |  |  |  |  |  |  |  |
|  | Mean (SD) | 10.3 | (1.55) | 10.3 | (1.52) | 10.3 | (1.53) |

IRS, indoor residual spraying of insecticide; IQR, inter-quartile range; SD, standard deviation
