# Supplementary material for: A Non-Inferiority, Individually Randomized Trial of Intermittent Screening and Treatment versus Intermittent Preventive Treatment in the Control of Malaria in Pregnancy
Source: PLoS One. 2015 Aug 10;10(8):e0132247. doi: 10.1371/journal.pone.0132247 (PMC4530893; doi:10.1371/journal.pone.0132247)
Supplement: S9 Table — (DOCX) [file pone.0132247.s017.docx]

## S9 Table

Placental malaria findings by intervention group and gravidity.

|  | **Primigravidae** | | | | **Secundigravidae** | | | |
| --- | --- | --- | --- | --- | --- | --- | --- | --- |
|  | IPTp-SP |  | ISTp-AL |  | IPTp-SP |  | ISTp-AL |  |
|  | N | % | N | % | N | % | N | % |
| **Placental histology** | N=908 |  | N=933 |  | N=755 |  | N=750 |  |
| Active infection (acute or chronic)^$^ | 207 | 22.8 | 234 | 25.1 | 201 | 26.6 | 173 | 23.1 |
|  |  |  |  |  |  |  |  |  |
| **Infection type** | N=908 |  | N=932 |  | N=755 |  | N=750 |  |
| Acute infections | 76 | 8.4 | 76 | 8.2 | 130 | 17.2 | 99 | 13.2 |
| Chronic infections | 131 | 14.4 | 157 | 16.8 | 71 | 9.4 | 74 | 9.9 |
|  |  |  |  |  |  |  |  |  |
| **Intervillous inflammation** | N=913 |  | N=937 |  | N=763 |  | N=760 |  |
| <5 per high powered field | 771 | 84.4 | 757 | 80.8 | 671 | 87.9 | 668 | 87.9 |
| 5-10 | 108 | 11.8 | 134 | 14.3 | 76 | 10 | 80 | 10.5 |
| 10-25 | 26 | 2.8 | 29 | 3.1 | 12 | 1.6 | 9 | 1.2 |
| >25 | 8 | 0.9 | 17 | 1.8 | 4 | 0.5 | 3 | 0.4 |
|  |  |  |  |  |  |  |  |  |
| **Among those without**  **active infection** |  |  |  |  |  |  |  |  |
|  |  |  |  |  |  |  |  |  |
| **Pigment in fibrin or macrophages*** | N=914 |  | N=936 |  | N=764 |  | N=758 |  |
| None | 258 | 28.2 | 214 | 22.9 | 238 | 31.2 | 229 | 30.2 |
| Mild | 222 | 24.3 | 230 | 24.6 | 302 | 39.5 | 278 | 36.7 |
| Moderate | 419 | 45.8 | 469 | 50.1 | 223 | 29.2 | 246 | 32.5 |
| Abundant | 15 | 1.6 | 23 | 2.5 | 1 | 0.1 | 5 | 0.7 |
|  |  |  |  |  |  |  |  |  |

Numbers shown for the according to protocol (ATP) population. ^$^ Co-primary outcome for study. Acute infection: Infected maternal erythrocytes and no or minimal pigment. Chronic infection: Infected maternal erythrocytes and moderate or abundant pigment. * Data for presence of pigment are shown only for children without active malaria infection. Numbers available for each analysis are shown, as complete information on malaria infection, inflammation and pigment in different locations was not available for all samples.
