# Supplementary material for: A Non-Inferiority, Individually Randomized Trial of Intermittent Screening and Treatment versus Intermittent Preventive Treatment in the Control of Malaria in Pregnancy
Source: PLoS One. 2015 Aug 10;10(8):e0132247. doi: 10.1371/journal.pone.0132247 (PMC4530893; doi:10.1371/journal.pone.0132247)
Supplement: S10 Table — (DOCX) [file pone.0132247.s018.docx]

| **Mutation** | **Burkina** | **Gambia** | **Ghana** | **Mali (Kita)** | **Mali (San)** |
| --- | --- | --- | --- | --- | --- |
| ***Dhfr*** |  |  |  |  |  |
| N51**I** | 49.1  (47.9 – 50.2) | 94.7  (94.3 – 95.2) | 65.1  (63.6 – 66.6) | 28  (27.4 – 28.7) | 38.5  (37.7 – 39.3) |
| C59**R** | 50.4  (49.3 – 51.6) | 93.2  (92.7 – 93.7) | 74.2  (72.8 – 75.5) | 29.1  (28.4 – 29.7) | 22.3  (21.7 – 23) |
| S108**N** | 57.8  (56.6 – 58.9) | 97.9  (97.6 – 98.2) | 69.8  (68.4 – 71.2) | 24.3  (23.8 – 24.9) | 38.4  (37.7 – 39.2) |
| I164**L** | 0 | 0 | 0 | 0 | 0 |
| ***Dhps*** |  |  |  |  |  |
| S436**A** | 77.7  (76.9 – 78.5) | 69.9  (69 – 70.8) | 71.7  (70.6 – 72.7) | 70.4  (69.8 – 71) | 37  (36.3 – 37.7) |
| S436**Y** | 2.1  (1.8 – 2.4) | 0 | 0.9  (0.6 – 1.1) | 0 | 6.3  (5.9 – 6.7) |
| S436**F** | 1.4  (1.2 – 1.7) | 0 | 1.2  (1 – 1.5) | 6.4  (6.1 – 6.7) | 1.2  (1.1 – 1.4) |
| A437**G** | 75.3  (74.5 – 76.2) | 9.1  (8.5 – 9.7) | 77.4  (76.4 – 78.4) | 15.2  (14.8 – 15.7) | 27.5  (26.8 – 28.2) |
| K540**E** | 0 | 0 | 0 | 0 | 0.73  (0.6 – 0.9) |
| A581**G** | 0 | 0 | 0 | 0 |  |
| A613**S** | 24  (23 – 25) | 0 | 6.1  (5.6 – 6.6) | 13.2  (12.3 – 14.1) | 13.2  (12.3 – 14.1) |

## S10 Table

Relative frequencies of molecular markers of SP resistance – by study centre.

*dhfr,* dihydrofolate reductase*; dhps* dihydropteroate synthase Numbers shown are frequencies of the indicated small nucleotide polymorphism (SNP) among all sequencing reads with coverage at that locus, expressed as a percentage; numbers in parentheses are 95% confidence intervals. Mutant amino acids are underlined and bolded for each locus.
