# Supplementary material for: A Non-Inferiority, Individually Randomized Trial of Intermittent Screening and Treatment versus Intermittent Preventive Treatment in the Control of Malaria in Pregnancy
Source: PLoS One. 2015 Aug 10;10(8):e0132247. doi: 10.1371/journal.pone.0132247 (PMC4530893; doi:10.1371/journal.pone.0132247)
Supplement: S11 Table — (DOCX) [file pone.0132247.s019.docx]

## S11 Table

Details of congenital abnormalities by study group.

| **Details** | **IPTp-SP group** | **ISTp-AL group** |
| --- | --- | --- |
| Cleft palate | 1 | 0 |
| Single nostril, hyperchromic spots on face | 0 | 1 |
|  |  |  |
| Extra finger (hanging 6th finger) | 8 | 5 |
| Extra finger other type | 1 | 1 |
| Extra finger and other hand abnormality | 1 | 0 |
| Other hand abnormality | 1 | 0 |
|  |  |  |
| Extra toe | 2 | 0 |
| Club toe | 1 | 0 |
| Missing toe | 0 | 1 |
|  |  |  |
| Leg abnormality | 0 | 2 |
| Limb reduction, other limb abnormality | 1 | 0 |
| Limb reduction, Missing finger | 0 | 1 |
|  |  |  |
| Spina bifida | 0 | 3 |
| Gastroschisis | 0 | 1 |
| Genito-urinary abnormality | 0 | 2 |
| Details not recorded | 0 | 1 |
| **Total** | **15** | **18** |
