# Supplementary material for: A Non-Inferiority, Individually Randomized Trial of Intermittent Screening and Treatment versus Intermittent Preventive Treatment in the Control of Malaria in Pregnancy
Source: PLoS One. 2015 Aug 10;10(8):e0132247. doi: 10.1371/journal.pone.0132247 (PMC4530893; doi:10.1371/journal.pone.0132247)
Supplement: S1 Text — (DOCX) [file pone.0132247.s020.docx]

# S1 Supplementary Methods

**Evaluation of blood films.**

Thick blood smears were stained with 2% Giemsa for 30 minutes or 10% Giemsa for 10 minutes and read by a trained microscopist at each study site. Parasite densities were calculated by counting the number of asexual parasites per 200 leukocytes (or per 500 leukocytes if the count was <10 asexual parasites/200 leukocytes), assuming a leukocyte count of 8,000/μl. A blood smear was considered negative when examination of 100 high power fields did not reveal asexual parasites. Each blood film was read by two microscopists. Slides discrepant in either positivity or parasite density were read by a third microscopist and a consensus result obtained using a standardised algorithm.^1^ In the case of a slide originally discrepant on positivity (i.e. one positive and one negative reading), the third reading was considered definitive. In the case of slides originally discrepant on parasite density (i.e. both positive, but parasite counts inconsistent), the geometric mean of the two closest readings (on a log scale) was used.

**Characterisation of resistance markers**

*P. falciparum* parasites obtained from study women were tested for resistance to SP using a pooled sequencing approach.^2^ Genomic DNA (gDNA) samples from microscopy-positive *P. falciparum* infections obtained from women who attended an antenatal clinic in Kita and San Mali (n=117 and n=130 respectively), Basse, the Gambia (n=50), Navrongo, Ghana (n=76), and Ziniaré, Burkina Faso (n=273) were pooled by clinic for analysis. Samples from the Mali and Burkina Faso sites were obtained from women enrolled in an antenatal SP efficacy study^3^ while those from Ghana and the Gambia were randomly-selected from women enrolled in the current study. After pooling gDNA from each site, the parasite *dhfr* and *dhps* genes were amplified separately in parallel nested PCR assays, PCR products were confirmed and cleaned, and these products were sequenced in multiplex fashion on a Roche 454 GS Junior second-generation sequencing platform. Sequencing reads were quality-filtered, partitioned by barcodes into study site, aligned to reference sequences, and scored manually for mutations in *dhfr* and *dhps* genes using Lasergene Genomics Core Suite with SeqMan NGen (v10.0, DNASTAR, Madison, WI, USA). The reported SNP frequencies are the proportion of reads containing the SNP among all reads with coverage at that locus; frequencies below 0.5% were considered potential sequencing errors and reported as zero.^2^

**Placental Histology**

A 2.5cm^3^ of the placental tissue was obtained from the maternal side of the placenta, midway between the umbilical insertion and the edge. The biopsy was fixed in 10% neutral, buffered formalin and kept at +4^o^C for a maximum period of four weeks prior to shipment to the Department of Pathology, Korle Bu hospital, Accra, Ghana for preparation of tissue blocks and slides. All histology slides were read at the Medical Research Council Unit, The Gambia by a single microscopist trained at the Department of Pathology, Centre for International Health Research (CRESIB), Hospital Clinic-Universitat de Barcelona. Slides were read according to a standard operating procedure (SOP) developed at the centre in Barcelona and results recorded on a standardised form.^4^ The presence of parasites, inflammation and pigment with fibrin or macrophages was recorded as described previously.

**Defining non-inferiority**

Defining a non-inferiority margin *a priori* for LBW and placental malaria (PM) was complicated by the fact the exact risk of these outcomes by centre was not known prior to the start of the trial; the odds ratio (OR) was used as a practical solution to this problem.^5^ For LBW, we specified an OR based on a consensus among the investigators that a 4% difference in the risk of LBW would be clinically acceptable if the risk was 20% in the IPTp-SP group i.e. the OR should be less than 1.263 (equating to a risk difference of 3.25% if the risk in the IPTp-SP group was 15%, and a 2.3% difference if the risk was 10%). The margin for placental malaria was specified as a 5% excess of active malaria infection in the ISTp-AL group, assuming a 25% prevalence of PM in the IPTp group (specified in terms of an OR less than 1.286). The non-inferiority margin for Hb concentration at the final follow-up visit before delivery was specified to exclude a reduction of 0.2 g/dL in Hb concentration in the ISTp-AL group relative to the IPTp group.

**References**

1. Swysen C, Vekemans J, Bruls M, et al. Development of standardized laboratory methods and quality processes for a phase III study of the RTS, S/AS01 candidate malaria vaccine. *Malar J* 2011; **10**: 223.
2. Taylor SM, Parobek CM, Aragam N, et al. Pooled deep sequencing of Plasmodium falciparum isolates: an efficient and scalable tool to quantify prevailing malaria drug-resistance genotypes. *J Infect Dis* 2013; **208**: 1998-2006.
3. Coulibaly SO, Kayentao K, Taylor S, et al. Parasite clearance following treatment with sulphadoxine-pyrimethamine for intermittent preventive treatment in Burkina-Faso and Mali: 42-day in vivo follow-up study. *Malar J* 2014; **13**: 41.
4. Ismail MR, Ordi J, Menendez C, et al. Placental pathology in malaria: a histological, immunohistochemical, and quantitative study. *Hum Pathol* 2000; **31**: 85-93.
5. Siqueira AL, Whitehead A, Todd S. Active-control trials with binary data: a comparison of methods for testing superiority or non-inferiority using the odds ratio. *Stat Med* 2008; **27**: 353-70.
