# Supplementary material for: A Non-Inferiority, Individually Randomized Trial of Intermittent Screening and Treatment versus Intermittent Preventive Treatment in the Control of Malaria in Pregnancy
Source: PLoS One. 2015 Aug 10;10(8):e0132247. doi: 10.1371/journal.pone.0132247 (PMC4530893; doi:10.1371/journal.pone.0132247)
Supplement: S2 Text — (DOCX) [file pone.0132247.s021.docx]

# S2 Text

# Trial protocol

**MA 5 PROTOCOL**

**A trial of intermittent preventive treatment with sulfadoxine-pyrimethamine versus intermittent screening and treatment of malaria in pregnancy**

| Principal Investigator | Prof. Brian Greenwood; Department of Infectious & Tropical Diseases, London School of Hygiene & Tropical Medicine, Keppel Street, London WC1E 7HT; +44 (0)20 7299 4707  [Brian.Greenwood@lshtm.ac.uk](mailto:udalessandro@itg.be) |
| --- | --- |
| Trial Coordinator: | Dr. Harry Tagbor; Department of Community Health, School of Medical Sciences, KNUST; +233 51 64236, +233 244 417 701; [Harry.Tagbor@lshtm.ac.uk](mailto:Harry.Tagbor@lshtm.ac.uk) |
| Protocol Number | MA05 |
| Study Protocol Acronym | IST – IPT |
| Project Title: | A trial of intermittent preventive treatment with sulfadoxine-pyrimethamine versus intermittent screening and treatment of malaria in pregnancy. |
| Project description: | A randomised, multi-centre, controlled trial of a standard SP - IPTp regimen (3 doses of SP in second and third trimester) and screening and treatment of malaria at scheduled antenatal clinic visits in the second and third trimester in pregnant women who sleep under an insecticide treated bed net undertaken in four West African countries. |
| **Study countries (sites):** | Burkina Faso (Ziniare), Ghana (Navrongo), Mali (San & Kita) and The Gambia (Basse) |
| Project start and end date: | January 2010 – December 2013 |
| **Date:** | 12/09/2009 |
| Sponsor: | London School of Hygiene & Tropical Medicine, Keppel Street, London WC1E 7HT  +44 (0)20 7299 4707 |
| **Safety monitor:** | Raouf A. OSSENI, PharmD, PhD Senior Consultant PharmaClin CRO  Email : [raouf.osseni@gmail.com](mailto:raouf.osseni@gmail.com)  Phone France: + 33 870 407 233 Benin Office : + 229 21 30 49 89 |

ABBREVIATIONS

| ACT | Artemisinin combination therapy |
| --- | --- |
| ANC | Antenatal clinic |
| BW | Birth weight |
| CI | Confidence interval |
| CRF | Case report form |
| DSMB | Data and Safety Monitoring Board |
| GCP | Good clinical practice |
| Hb | Haemoglobin |
| HRP2 | Histidine rich protein - 2 |
| IEC | Independent ethics committees |
| IPTp | Intermittent preventive treatment in pregnancy |
| IRB | Institutional review board |
| ITN | Insecticide treated net |
| LLIN | Long lasting insecticide treated net |
| MRC | Medical Research Council |
| PI | Principal investigator |
| RDT | Rapid diagnostic test(ing) |
| SOP | Standard operating procedure |
| SP | Sulfadoxine pyrimethamine |

| **INVESTIGATORS** | | |
| --- | --- | --- |
| **Mali** | | |
| Name | Dr Kassoum Kayentao | Principal Investigator |
| Contact | Medical Research and Training Centre  BP: 1805  Bamako, Mali | |
| Telephone | +(223) 222 81 09 or (223) 646 01 73 | |
| Fax | + 223 222 81 09 | |
| Email: | [kayentao@MRTCBKO.org](mailto:kayentao@MRTCBKO.org) | |
|  | | |
| **Burkina Faso** | | |
| Name | Dr Sheick Coulibaly | Principal Investigator |
| Contact | Université de Ouagadougou  01 BP 7021Ouagadougou 01  Ouagadougou, Burkina Faso | |
| Telephone | +226 50 30 70 64/65 | |
| Fax | +226 5033 30 72 42 | |
| Email: | [sheickoumar2@yahoo.fr](mailto:sheickoumar2@yahoo.fr) | |
|  | | |
| **The Gambia** | | |
| Name | Dr Kalifa Bojang | Principal Investigator |
| Contact | Medical Research Council Laboratories  Atlantic Boulevard, Fajara  P O Box 273  Faraja, The Gambia | |
| Telephone | +220 4495442/6  or 4494072/9 | |
| Fax | +220 4495919 | |
| Email: | [kbojang@mrc.gm](mailto:kbojang@mrc.gm) | |
|  | | |
| **Ghana** | | |
| Name | Dr. Abraham Hodgson | Principal Investigator |
| Contact | Ghana Health Research Centre, Navrongo  P.O.Box 114  Navrongo, Ghana | |
| Telephone | +233 742 22310 /22380 /22651 | |
| Fax | +233 742 22320 | |
| Email: | [abrahamhodgson@hotmail.com](mailto:abrahamhodgson@hotmail.com) | |

**COLLABORATORS AND AFFILIATIONS**

Dr. Paul Milligan London School of Hygiene & Tropical Medicine

Prof. Daniel Chandramohan London School of Hygiene & Tropical Medicine

Prof. Feiko Ter Kuile Liverpool School of Tropical Medicine

**SUMMARY**

**Rationale:** The incidence of malaria, including the incidence in pregnant women, is declining in many African countries. Thus, there is a need to re-examine the efficacy and cost effectiveness of giving intermittent preventive treatment with sulphadoxine-pyrimethamine in pregnancy (SP-IPTp) on several occasions during pregnancy, an intervention that is threatened by increasing resistance to SP. Possible alternatives to SP-IPTp need to be explored. This applies especially to areas with highly seasonal malaria transmission where women are at risk for only a short period of the year.

Overall goal: The goal of this project is to determine whether in pregnant women who sleep under a long lasting insecticide treated bed net, screening and treatment at each scheduled antenatal clinic visit is as effective in protecting them from anaemia, low birth weight and placental infection as SP-IPTp.

**Methods:** Primigravidae and secundigravidae who present at antenatal clinics in study sites in four West African countries (Burkina Faso, Ghana, Mali and The Gambia) will be randomised to one of two groups. All women will be given a long lasting insecticide treated bed net on first presentation at the antenatal clinic. Women in group 1 (reference group) will receive SP-IPTp according to the current WHO guidelines. Those in group 2 will be screened with a rapid diagnostic test at each scheduled antenatal clinic visit and treated if parasitaemic. Approximately 5000 women will be recruited, 2500 in each group. Women will be encouraged to deliver in hospital where maternal haemoglobin and birth weight will be recorded and a placental sample obtained. Those who deliver at home will be visited within a week of delivery and maternal haemoglobin and infant weight recorded. Mothers and infants will be seen again six weeks after delivery. The primary end points of the trial will be birth weight and anaemia at 38 weeks (+/-2 weeks) of gestation. The study is powered to show non-inferiority of group 2 compared to group 1. The costs and cost effectiveness of each intervention will be evaluated.

**Implications:** The study will provide information to national malaria control programmes on whether there are alternative, safe and effective methods to the SP IPTp regimen for reducing the burden of malaria in pregnancy.

BACKGROUND AND RATIONALE

There is convincing evidence from five trials that insecticide treated bed nets (ITNs) used during pregnancy are beneficial to both mother and newborn baby ^2^. Thus, strenuous efforts are being made to make ITNs accessible to pregnant women across Africa through the free distribution of ITNs at antenatal clinics and through the use of vouchers ^3^. Substantial success is now being achieved in this endeavour with coverage rates reaching 50% in some countries and many countries are making progress towards meeting the Abuja target of 60% coverage. ITNs have social benefits, providing protection against the nuisance of biting insects and provide protection against some other vector borne diseases; they have no significant side effects. Thus, in most parts of Africa, ITNs are likely to remain one of the primary means of protection against malaria in pregnancy (MiP). Increasing use is also being made of indoor residual spraying (IRS) as a means of malaria vector control but its efficacy in preventing MiP has not been well established.

There is also strong evidence that intermittent preventive treatment (IPTp) with SP provides protection against maternal anaemia and low birth weight (LBW) in primigravidae and secundigravidae when used as the only means of malaria prevention in pregnancy ^4^. However, there is little information on the added benefit provided by IPTp in women who sleep under an ITN, and it is not clear whether women who are protected by ITNs also need IPTp. In an initial study conducted in western Kenya, the combination of IPTp and ITNs was slightly more effective than ITNs alone (PE 56% vs. 42%) but only in primigravidae and not in secundigravidae ^5^. In the Gambia, where the prevalence of HIV infection and SP resistance are low, no beneficial effect from IPTp with SP on anaemia or birth weight was seen in multigravidae, including secundigravidae, with the exception of a small sub-group of women who did not use a bed net ^6^**.** More recently, a trial in Mozambique showed that a two-dose SP IPTp regimen was associated with a reduction in some indicators of malaria infection but that these were not translated into significant improvements in maternal or birth outcomes ^7^. Thus, the limited information available suggests that IPTp may only have a small additive effect, if any, when given to HIV negative pregnant women who sleep under an ITN.

The added benefit which IPTp with SP might provide to pregnant women sleeping under an ITN is likely to be especially marginal in communities with (a) substantial resistance to SP (b) highly seasonal transmission of malaria with nearly all new infections being acquired during a few months of the year, or (c) a declining incidence of malaria, a trend being recorded in an increasing number of countries in Africa where IPTp with SP is currently the recommended strategy. In these situations, a large number of pregnant women receive antimalarials unnecessarily and there is a need to find alternative approaches to the prevention of malaria in pregnancy other than year round administration of SP-IPTp.

One potential, alternative control strategy is screening for malaria at routine antenatal clinics and treatment of positive women (IST). The development of simple and affordable rapid diagnostic tests (RDTs) provides a potential way of doing this in the clinic; some of these tests are able to detect low levels of *Plasmodium falciparum* peripheral blood parasitaemia. A number of studies have investigated the use of RDTs for the diagnosis of malaria at delivery and/or in the detection of placental malaria ^8-12^ or in the diagnosis of malaria in pregnant women attending an antenatal clinic ^13-15^ but the findings of these studies have not been consistent. This may be due in part to differences in the sensitivity and specificity of the various RDTs that have been used. A recent study by WHO and FIND of 40 commercially available RDTs showed marked differences in the performance of different tests ^16^. Following discussions with WHO and FIND a comparative study has started in Burkina Faso, Mali and Ghana (Kumasi) of the sensitivity of FIND ‘highly recommended’ LDH and HRP2 based RDTs in the diagnosis of malaria in pregnancy using PCR as the gold standard. The result of this pilot study in 204 women at each site will be used to decide on the RDT used in the trial described in this proposal.

A pilot study pilot to test the feasibility of IST with an RDT has recently been completed in Ghana. The study was conducted in 3330 women of all gravidities at three general hospitals near to Kumasi. The study area is one of derived forest with perennial malaria transmission although with a pronounced seasonal peak. The entomological inoculation rate in the neighbouring area of Kintampo was recently estimated to be 267 infective bites per year. The study was designed to show that IST with treatment with SP or with an artesunate + amodiaquine, first line treatment for malaria in Ghana, was not inferior to SP-IPTp in preventing anaemia and low birth weight and to determine if IST would be feasible and acceptable in a busy, routine antenatal clinic. Preliminary results of the trial are as follows-

1. substantial transmission of malaria was taking place in the study area at the time of the study - the prevalences of parasitaemia at enrolment in primigravidae and in multigravidae were 29.6% and 10.2% respectively, prevalence rates comparable to those seen in other moderately high transmission areas,
2. IST was not inferior to SP-IPT for the primary trial end point - anaemia at 36-40 weeks of pregnancy. Mean haemoglobin concentrations and the prevalence of moderate/severe anaemia (Hb< 8g/dl) at this time point were almost identical in each study group (SP-IPTp - 48.4%, IST-SP - 46.3%, IST-AQ+AS - 47.8% respectively). This was also the case when primigravidae and secundigravidae were considered separately,
3. IST was not inferior to SP-IPTp in preventing low birth weight; the prevalence of low birth weight was almost identical in each treatment group, (SP-IPTp – 11.3%, IST-SP – 10.8%, IST-AQ+AS – 13.6% respectively) even among primigravidae and multigravidae,
4. the prevalence of parasitaemia at 36 – 40 weeks of pregnancy was similar in all three groups (SP-IPTp – 12.0%, IST-SP – 13.0%, IST-AQ+AS -12.3% respectively),
5. the incidence of clinical attacks of malaria was similar in all three groups,
6. the outcome of pregnancy was similar in all groups,
7. IST was well received by pregnant women and was manageable within busy antenatal clinics.

Because of funding constraints it was not possible to study the impact of the three interventions on the prevalence of placental malaria.

The results of this pilot study suggest that even in an area of moderately high malaria transmission, IST is a safe, effective and feasible strategy for the control of malaria in pregnancy. However, this needs to be confirmed in other sites with different patterns of malaria and more attention needs to be given to the impact of IST on malariometric measurements including the prevalence of placental malaria. Therefore, we propose to undertake a multicentre trial which will seek to confirm the results of the Kumasi study on the key clinical outcomes for studies of malaria control in pregnancy - anaemia and low birth weight - and which will also look at the impact of this intervention on malaria infection of the placenta.

METHODS

Overall study design

A two-arm, multi-centre, open, randomised, controlled, non-inferiority trial comparing two malaria control strategies in pregnancy is proposed. The study groups will be as follows: -

**Group 1**: SP-IPTp SP according to WHO recommendations; women will receive at least two doses of SP during their pregnancy, one at each of the recommended ante-natal visits during the 2nd and 3rd trimester.

**Group 2:** Scheduled intermittent screening by RDT and treatment of those who are RDT positive during ante-natal clinic visits in the 2nd and 3rd trimester.

Women in ***group 1*** will be the reference group.

All study women will be provided with an LLIN at their first attendance at the ANC and given instructions on how to use it. Random home visits will be made to check on net usage during the pregnancy. Standardised dosages of ferrous sulphate (200mg) and folic acid (5mg) will be given to study women in each study site as there are variations in national guidelines regarding these drugs.

Hypothesis

The hypothesis being addressed by this trial is that management of malaria in pregnancy in women who sleep under an LLIN by screening using a RDT and treating women who are parasitaemic at scheduled antenatal clinic visits is not inferior to SP-IPTp in protecting against low birth weight, anaemia and malaria infection of the placenta.

Objectives

Primary objective

To determine the optimum method of controlling malaria in pregnancy in women who sleep under an LLIN in areas of seasonal malaria transmission.

Specific Objectives

1. To determine if scheduled screening and treatment during antenatal clinic visits is as effective in protecting against low birth weight, anaemia and malaria infection of the placenta as a standard SP-IPTp in primigravidae and secundigravidae who sleep under a long lasting ITN.
2. To evaluate the cost-effectiveness of delivering the two strategies measured as the cost per cases of maternal anaemia and antenatal malaria averted.

Outcomes

Primary outcomes

There will be three co-primary outcomes – low birth weight, anaemia at 38 weeks (+/- 2 weeks) of gestation and placental malaria.

Secondary outcomes

Secondary outcomes will include -

- The prevalence of anaemia at the time of delivery or shortly afterwards.
- The prevalence of peripheral blood parasitaemia at 38 weeks (+/- 2 weeks) of gestation, at the time of delivery and six weeks later.
- Episodes of clinical malaria during the course of the pregnancy.
- Serious adverse events in the mother.
- Adverse outcome of pregnancy – abortions, still births and neonatal deaths.
- Occurrence of congenital abnormalities.
- Feasibility and costs of each approach to the control of malaria in pregnancy.
- Cost per cases of maternal anaemia (severe and non-severe) and peripheral malaria averted.
- Acceptability of each approach by pregnant women and antenatal clinic staff.

Sample size

The trial is powered to show that IST is not inferior to SP-IPTp in preventing low birth weight (LBW). Review of the literature indicates that the prevalence of LBW (birth weight <2,500 g) in primigravidae or secundigravidae who receive a full course of SP-IPTp (group 1) is likely to be about 10% with some variation between sites. To demonstrate with 90% power and at a 0.025% significance level that the prevalence of low birth weight among infants born to mothers in group 2 is not more than 3% above that of infants born to mothers in group 1, approximately 2,000 women are needed in each group. This sample size will give a study with 90% power at a 0.025% significance level to detect a 50g difference in mean birth weight between study groups, the minimum difference between groups that would be acceptable as the basis for a change in policy. A study of this size would have similar power to detect differences in Hb concentration between groups of 0.2g/dl or more. A study of this size will have around 90 % power to detect a difference in the prevalence of placental malaria between groups of 5% or more. However, we estimate that only about 50% of women will deliver in a hospital or health centre and provide a placental biopsy. With a reduced sample size of 2500, the study will have 80% power to detect differences in placental malaria between groups of 5% or more.

It is assumed that drop-out during the course of the study will be in the range of 10%-20% with some variation between sites. Thus, it is proposed to recruit 2,500 women per group or 5000 overall. Previous experience indicates that it will be possible to recruit the required number of women at each site (approximately 1,250) within a period of one year. Thus, the total study will last 3 years.

Study sites

This is a multi-centre study which will take place in four West African countries - Burkina Faso, Ghana, Mali and The Gambia. Malaria is endemic in these countries with marked seasonal variation and relatively low HIV prevalence. SP-IPTp is recommended policy within the region ^17^. The prevalence of malaria in pregnancy varies within the West African region. Background information on each study site is summarized in Table 1. Further information about each site is provided below.

Burkina Faso; health district of Ziniaré (Province of Oubritenga)

The Burkinabe component of the study will be conducted in the health district of Ziniaré which is situated in the province of Oubritenga north of Ouagadougou, in Burkina Faso. The total population of the district was 246,900 in 2004 and the adult female population was 67,900. Twelve thousand pregnancies are expected per year. Child mortality fluctuates between 23 and 28 per 1000 live births. The district has one district hospital (50 km from Ouagadougou) and 39 health centres/dispensaries. The climate of the area is characteristic of the Sudan savannah with a dry, cool season from November to February, a dry, hot season from March to May and a rainy season from June to October. The mean annual rainfall is about 650 mm and the average annual temperature ranges between 23^o^ and 33°C. Malaria is endemic and is the first cause of consultation and hospitalization. The EIR is in the range of 100-300 infectious bites per person per year. Malaria transmission is markedly seasonal. A trial of insecticide treated curtains (ITC) was carried out in the area in the 1990s. The malaria in pregnancy prevention policy in the area comprises ITNs and SP-IPTp. The current treatment policy of uncomplicated malaria in pregnancy is oral quinine (25mg/day for 5 days) but artesunate plus amodiaquine or Coartem may be used in the second and third trimesters of gestation. The treatment policy for the general population is amodiaquine-artesunate or Coartem. Routine HIV screening is carried out at fist booking after voluntary counselling unless the woman explicitly declines to be tested. Treatment is offered to those who are HIV positive. The trial will be conducted by the UFR / SDS (School of Medicine), University of Ouagadougou with some support from the Centre National de Recherche et de Formation sur le Paludisme (CNRFP). This site has expertise in conducting trials in malaria in pregnancy, malaria epidemiology and drug resistance. Recent studies include a treatment trial comparing SP and chloroquine and a study of intestinal parasite in pregnancy ^18^.

Ghana: Navrongo Health Research Centre, Navrongo.

The Ghanaian component of the study will be conducted in the Kassena-Nankana district of northern Ghana, which lies within the Sudan savannah belt of West Africa. Navrongo has an annual rainfall of 850-950mm, most of which falls between May and September. The district has a population of about 145,000 and the economy is dominated by subsistence farming. Educational attainment is generally low. The district has 4 health centres, 3 clinics and a 140-bed hospital that serves as a referral centre. All these facilities provide antenatal care and have facilities for delivery. These are complemented by services provided by community-based community health officers who offer ante-natal services. Trained midwives undertake deliveries as well. The district serves as a national HIV sentinel site. Routine HIV screening is carried out at fist booking after voluntary counselling unless a woman explicitly declines to be tested. Treatment is offered to those who are HIV positive. Malaria transmission in the area is highly seasonal, following the pattern of rainfall. *P. falciparum* accounts for nearly all clinical malaria infections. The EIR is approximately 200 infectious bites per year. Amodiaquine /artesunate is the currently recommended first line treatment for malaria outside pregnancy. The current treatment policy of uncomplicated malaria in pregnancy is oral quinine (25mg/day) in divided doses for 7 days. Artesunate plus amodiaquine or Coartem may be used in the second and third trimesters of gestation. The trial will be based at the Navrongo Health Research Centre, a well equipped centre which has extensive experience with large field trials, including trials of ITNs and meningococcal and rotavirus vaccines. A large trial of IPTp with amodiaquine and SP + amodiaquine has recently been completed which involved over 3600 pregnant women ^19-20^.

Mali: San and Kita field sites

The trial will based at the community health centres of two medium sized, semi-rural towns (San and Kita) in north-eastern Mali, with a total population of approximately 75,000 inhabitants. San is located 440 kilometres northeast of Bamako in the Segou region of Mali; Kita is 140 km North-west of Bamako. Residents of both towns are of relatively similar socio-economic status. These areas have a climate typical of the Sudan savannah with a long dry season and a shorter rainy season when most malaria transmission occurs, extending from June to August (San) or September (Kita). The predominant malaria parasite is *P. falciparum* and the EIR is about 20 infectious bites per year. SP retains very high efficacy in children (more than 93% cure at day 28 after the start of treatment) and ITN use is more than 60% at both sites. Staff of the Malaria Research and Training Centre, Bamako will be responsible for conduct of the trial. This well equipped centre has extensive experience of malaria research including the conduct of treatment and vaccine trials. MRTC is involved in all aspects of research on malaria and has an excellent track record for running complex field and intervention studies (Kayentao et al., 2005).

The Gambia: MRC Laboratories, Basse.

The trial will be conducted in Upper River Division. The study area comprises Basse town and the surrounding rural areas. A demographic surveillance system is in place covering a population of approximately 120,000. The climate of the area is typical of the Sudan savanna with an annual rainfall of around 700 mm a year which falls predominantly during the months of July – November, a little later that in the other sites in this trial. *P. falciparum* is the dominant malaria parasite. The EIR is in the range of 10-20 infectious bites per year and has probably fallen during the past few years. The study area is served by Basse health centre and by several dispensaries and health centres. Basse health centre has in-patient facilities for 100 patients. It has laboratory facilities for haematological, biochemical and parasitological investigations and X-ray. The current treatment policy of uncomplicated malaria in pregnancy is oral quinine (25mg/day for 5 days) but Coartem may be used in the second and third trimester of pregnancy. The treatment policy for the general population is Coartem. Routine HIV screening is carried out at fist booking after voluntary counselling and treatment is offered to those who are HIV positive. Basse health centre is supported by the well equipped MRC field station in Basse town. MRC is currently conducting surveillance studies on the causes of diarrhoea and acute respiratory infections in Upper River Division. Studies on the prevention of malaria in children using vaccination and intermittent preventive treatment have previously been conducted successfully in Upper River Division.

Study period

Preparatory activities commenced in August 2008 but recruitment into the study will probably not start until the end of 2009 and run for the next 24 months. Details are shown in table 2.

Study population

Primigravidae and secundigravidae will be considered for potential inclusion in the trial when they present for the first time at an antenatal clinic.

Inclusion criteria

1. Presence of a first or second pregnancy.
2. Gestation between 16 to 24 weeks inclusive at first booking as determined by symphysio-fundal measurements.
3. Provision of informed consent to join the trial.
4. Residence in the study area and intention to stay in the area for the duration of the pregnancy.

Exclusion criteria

1. Absence of informed consent.
2. An intention to leave the study area before delivery.
3. A history of sensitivity to sulphonamides.
4. Clinical AIDS or known HIV positivity.
5. Presence of any systemic illness likely to interfere with interpretation of the results of the trial.

Enrolment & follow-up procedures

A schematic profile of the study is shown in Figure 1. All study pregnant women will be recruited during antenatal clinic sessions held at the study sites over a period of one year. Teams of midwives and their assistants trained by principal investigators (PIs) at the various sites will conduct the recruitment during antenatal clinic hours. The recruitment processes will be supervised by the PIs.

Visit 1: - enrolment of study women

Activities conducted on this first attendance at the antenatal clinic will include –

1. Asking all pregnant women who report to a study antenatal clinic if they are interested in joining the trial.
2. Obtaining verbal consent for screening from women who express an interest in joining the trial.
3. Screening women who give verbal consent for this procedure and determining if they meet the inclusion criteria.
4. Obtaining written, informed consent from women who meet the inclusion criteria.
5. Randomisation of women who satisfy the inclusion criteria and who have given consent in blocks of 12 to one of two study groups using a computer generated randomization list: Group 1 standard SP-IPTp arm and Group 2 RDT screening and treatment with Coartem .
6. Assessment of all pregnant women clinically and obstetrically.
7. Obtaining blood for baseline investigations including measurement of haemoglobin concentration, preparing thin and thick blood films for malaria parasite counts (to be read subsequently by a project microscopist) and filter paper blood spots for molecular studies.
8. Provision of a long-lasting insecticide treated bed net (LLIN) to all study women with instructions on how to use this.
9. Provision of a monthly, pre-packed supply of prophylactic iron (ferrous sulphate 200mg containing 65mg iron) and folic acid (5mg) tablets to all women with instructions to take one tablet of each daily.
10. Conducting RDT screening on women in group 2 and treating with Coartem any who are positive.
11. Provision of SP-IPTp to all women in group 1.
12. Provision of information on the date for the next scheduled follow up visit.

Visits 2 & 3: Scheduled antenatal clinic visits

Women will be asked to return to the antenatal clinic at 24-26 and at 32- 34 weeks of gestation as specified in national guidelines. At these follow-up visits the following activities will be undertaken -

1. Provision of routine antenatal care to all study women.
2. Reminding all women about the importance of using their LLIN.
3. Provision of pre-packed supplies of prophylactic iron and folic acid tablets in the doses indicated above to last until the next scheduled visit.
4. Testing women in group 2 with an RDT and treating those who are positive with Coartem.
5. Giving women in group 1 a dose of SP up to a maximum of 3 doses during the pregnancy.
6. Provision of information on the date for next scheduled follow up visit.

All study women who present with a history of fever during an unscheduled visit will be tested for malaria with an RDT and, if positive treated with Coartem.

Visit 4: assessment visit

At this additional assessment visit the following activities will be undertaken –

1. Provision of routine antenatal care to all study women.
2. Reminding all women about the importance of using their LLINs.
3. Provision of all women with pre-packed supplies of prophylactic iron and folic acid tablets in the doses indicated above sufficient to last until the next scheduled visit.
4. Collection of a blood sample for determination of haemoglobin concentration, preparation of blood films and a filter paper blood spot.
5. Requesting woman to deliver in hospital.

Visit 5: Delivery

The following activities will be undertaken for women who deliver in hospital -

1. Collection of a blood sample for determination of haemoglobin concentration, preparation of peripheral blood films and a blood spot.
2. Obtaining a cord blood sample from all women.
3. Obtaining a smear from the placenta from all women.
4. Obtaining a placental biopsy from all women.
5. Recording birth weight on all newborns.
6. Determining the Ballard score on all newborns.
7. Checking all newborns for congenital abnormalities.

The following activities will be undertaken in the case of women who deliver in a health centre -

1. Collection of a blood sample for determination of haemoglobin concentration, preparation of peripheral blood films and a blood spot within seven days of birth from all study women.
2. Recording weight of all infants within seven days of birth.

Visit 6: - Post natal follow-up

At 6 weeks post partum all women will be visited and the following activities will be undertaken -

1. Assessment of the condition of all mothers and their babies including examination for any congenital abnormalities.
2. Collection of a blood sample for determination of haemoglobin concentration, preparation of peripheral blood films and a blood spot.

Assessment of adverse events

During the course of the study, any adverse events (AEs) which occur will be appropriately recorded, managed and reported. On the seventh day following administration of an antimalarial drug to study women, field workers will follow up women to ascertain and record any untoward medical occurrence, including occurrences which are not necessarily caused by or related to the antimalarial. In addition, midwives will record all occurrences of adverse pregnancy outcomes such as a miscarriage or spontaneous abortion (<28 weeks of gestation), stillbirth (>28weeks) or the occurrence of any congenital malformation. A clinician will confirm any record of a congenital deformity by thorough examination. A Standard Operating Procedure (SOP) which describes the processes and guidelines for recording, managing and reporting adverse events and serious adverse events for clinical trials in accordance with the guidelines set by the Malaria in Pregnancy (MIP) Consortium has been developed. The SOP also defines clearly what constitute an adverse event and the assessment of any event for causality and expectedness.

Laboratory methods

Standardised SOPs have been developed for Hb measurement, preparation of blood smears, conduct of RDTs and preparation of placental smears and biopsies.

**Haemoglobin measurement:** Haemoglobin concentrations will be measured from finger prick blood samples using Hb 301 Hemocue™ analysers (HemoCue, Anglom, Sweden). The measurements will be done four times for each woman according to the manufacturer’s instructions; first at recruitment, then at 32-34 weeks, at 38 weeks +/-2 weeks, at the time of delivery or shortly afterwards and at 6 weeks post-partum.

**Blood film: -** Thin and thick blood smears will be obtained from all women on presentation, at 38 weeks (+/- 2 weeks), at delivery and post-partum. In addition; blood films will be made at any time that a study woman presents at an unscheduled visit with suspected malaria. Thick blood smears will be stained with 2% Giemsa for 30 minutes and read by trained microscopists at each site. Parasite densities will be calculated by counting the number of asexual parasites per 200 leukocytes (or per 500 leukocytes if the count is <10 asexual parasites/200 leukocytes), assuming a leukocyte count of 8,000/μl. A blood smear will be considered negative when the examination of 100 high power fields does not reveal asexual parasites. For quality control, a 10% random sample of slides will be read by microscopists from a designated referral centre. These results will be used to monitor the slide reading of individual microscopists and to retrain those who need it.

**Rapid diagnostic tests (RDTs):**  The choice of RDT will be based on the results of an on-going pilot study. The same RDT will be used at all sites. Members of recruitment teams will receive hands-on instruction from local PIs in preparation and interpretation of the RDTs according to the manufacturer instructions.

**Preparation of placental smears and biopsies:** Immediately after all deliveries by study women which occur in a health facility, trained midwives will obtain and wash placental samples with sterile normal saline. A small piece of placental tissue (0.5 cm^3^) will be excised from the centre of the maternal side of the placenta to prepare impression smears. A larger placenta biopsy (1.5 by 1.5 by 1 cm) will be obtained and fixed in 50 ml of 10% neutral buffered formalin adjusting the pH at 7.0 and individually labelled to help identification. Pending histological evaluation, all biopsies will be kept at 4 °C. The placental biopsies will be processed and embedded in paraffin wax by standard techniques. Paraffin sections 4 μm thick will be stained with hematoxylin and eosin (H&E) and Giemsa’s stain for histopathological assessment locally if the expertise exists or sent to a central location for evaluation ^21-22^.

Data management

The same pre-printed case report forms (CRFs) will be used to capture enrolment and follow up data at all trial centres. All CRFs will be received and registered by the data management teams. All subsequent data processing activities (data entry, data checking and quality control) will be done using a Microsoft Access 2007 computerized database. The “Compare Programme” a Microsoft Access data checking programme for comparing double entered data will be used weekly to verify that records in both databases are the same. Double data entry and data checking will be done locally at the trial centres. All discrepancies and queries will be resolved manually by data management teams (clerks and data managers) at each site. A copy of the database will be stored on a removable mass storage tool as a safeguard against loss. The trial coordinator will collect double-entered, checked and verified data from each site for onward transmission to the trial statistician who will be responsible for maintaining the combined database for the trial and for ensuring its security.

STATA (version 10) software will be used to clean and validate entered data and for performing the main analysis.

Standardised SOPs and quality control

Trial specific, written standard operating procedures (SOPs) are being developed for investigating teams to be used, with local modifications in all the centres to ensure consistency. A meeting of all local PIs and data managers will be held to discuss and agree on the set of drafted standard operating procedures (attached). This will provide a good opportunity for study investigators to reach a consensus on all issues to ensure that data quality is high.

ANALYSIS

The principal analyses for primary and secondary outcomes will be ‘’per protocol’’ but an "intention-to-treat" analysis will also be done. For inclusion in the ‘’per protocol’’ analysis Information on the primary end-point must have been obtained. In addition women in the SP-IPTp group must have received 2 or more doses of SP-IPTp whilst women in the IST group must have received two or more scheduled RDT tests. For a woman to be considered in the ‘’intention to treat’’ analysis she must have been randomised and provided data on the primary end-point.

The 95% confidence interval for the difference in the effects of interventions will be calculated and used to draw conclusions from the trial. Non-inferiority will be demonstrated if the calculated CI lies entirely above the pre-defined noninferiority margin. If the lower margin or the entire range of the calculated CI lies below the pre-defined non-inferiority margin, the trial will not have demonstrated noninferiority. A p-value associated with the null hypothesis will be calculated to aid assessment of the strength of evidence in favour of noninferiority. A comprehensive statistical analysis plan will be developed prior to data analysis and submitted for approval to the Data and Safety Monitoring Board (DSMB).

ETHICS

The trial protocol has been submitted to accredited institutional review boards (IRB)/independent ethics committees (IEC) in participating countries and to the London School of Hygiene and Tropical Medicine ethics committee for approval. It has been approved by the London School of Hygiene and Tropical Medicine ethics committee and by the appropriate ethics committees in Burkina Faso and Mali. Review in Ghana and The Gambia is pending.

Local principal investigators will hold meetings with community leaders and women’s groups to describe the purpose of the study and the procedures involved and give them the opportunity to seek clarifications. Eligible women will be assured that their choice to participate or not to participate will not affect their right to access the best available care at the antenatal clinic and the hospital. Women who agree to participate will be thoroughly educated on malaria in pregnancy and its control, and then be required to give informed consent to participate by either signing or thumb printing a consent form translated into the local language before they are enrolled. All expenditure involved will be paid for by the project for each woman.

A data and safety monitoring board (DSMB) has been constituted. The board will be responsible for

- Regularly monitoring the progress of the data and safety issues concerned with the study.
- Reviewing the PI’s reports on serious adverse events and making recommendations on further progress of the study.

Members of the board are -

1. Prof. Geoff Target Chairman
2. Dr Gloria Quansah Member
3. Professor Ousman Nyan Member
4. Dr. Charlemagne Ouedraogo Member
5. Dr Sow Member

REFERENCES

Clerk, C. A., Bruce, J., Affipunguh, P. K., Mensah, N., Hodgson, A., Greenwood, B. and Chandramohan, D. (2008). "A randomized, controlled trial of intermittent preventive treatment with sulfadoxine-pyrimethamine, amodiaquine, or the combination in pregnant women in Ghana." J Infect Dis **198**(8): 1202-11.

Clerk, C. A., Bruce, J., Greenwood, B. and Chandramohan, D. (2009). "The epidemiology of malaria among pregnant women attending antenatal clinics in an area with intense and highly seasonal malaria transmission in northern Ghana." Trop Med Int Health **14**(6): 688-95.

Coulibaly, S. O., Nezien, D., Traore, S., Kone, B. and Magnussen, P. (2006). "Therapeutic efficacy of sulphadoxine-pyrimethamine and chloroquine for the treatment of uncomplicated malaria in pregnancy in Burkina Faso." Malar J **5**: 49.

Crawley, J., Hill, J., Yartey, J., Robalo, M., Serufilira, A., Ba-Nguz, A., Roman, E., Palmer, A., Asamoa, K. and Steketee, R. (2007). "From evidence to action? Challenges to policy change and programme delivery for malaria in pregnancy." Lancet Infect Dis **7**(2): 145-55.

Gamble, C., Ekwaru, J. P. and ter Kuile, F. O. (2006). "Insecticide-treated nets for preventing malaria in pregnancy." Cochrane Database Syst Rev(2): CD003755.

Kilian, A. H., Kabagambe, G., Byamukama, W., Langi, P., Weis, P. and von Sonnenburg, F. (1999). "Application of the ParaSight-F dipstick test for malaria diagnosis in a district control program." Acta Trop **72**(3): 281-93.

Leke, R. F., Djokam, R. R., Mbu, R., Leke, R. J., Fogako, J., Megnekou, R., Metenou, S., Sama, G., Zhou, Y., Cadigan, T., Parra, M. and Taylor, D. W. (1999). "Detection of the Plasmodium falciparum antigen histidine-rich protein 2 in blood of pregnant women: implications for diagnosing placental malaria." J Clin Microbiol **37**(9): 2992-6.

Mankhambo, L., Kanjala, M., Rudman, S., Lema, V. M. and Rogerson, S. J. (2002). "Evaluation of the OptiMAL rapid antigen test and species-specific PCR to detect placental Plasmodium falciparum infection at delivery." J Clin Microbiol **40**(1): 155-8.

Mbaye, A., Richardson, K., Balajo, B., Dunyo, S., Shulman, C., Milligan, P., Greenwood, B. and Walraven, G. (2006). "A randomized, placebo-controlled trial of intermittent preventive treatment with sulphadoxine-pyrimethamine in Gambian multigravidae." Trop Med Int Health **11**(7): 992-1002.

Menendez, C., Romagosa, C., Ismail, M. R., Carrilho, C., Saute, F., Osman, N., Machungo, F., Bardaji, A., Quinto, L. and Mayor, A. (2008). "An autopsy study of maternal mortality in Mozambique: the contribution of infectious diseases." PLoS Med **5**: e44.

Mockenhaupt, F. P., Ulmen, U., von Gaertner, C., Bedu-Addo, G. and Bienzle, U. (2002). "Diagnosis of placental malaria." J Clin Microbiol **40**(1): 306-8.

Newman, R. D., Moran, A. C., Kayentao, K., Benga-De, E., Yameogo, M., Gaye, O., Faye, O., Lo, Y., Moreira, P. M., Duombo, O., Parise, M. E. and Steketee, R. W. (2006). "Prevention of malaria during pregnancy in West Africa: policy change and the power of subregional action." Trop Med Int Health **11**(4): 462 - 469.

Njagi, J. K., Magnussen, P., Estambale, B., Ouma, J. and Mugo, B. (2003). "Prevention of anaemia in pregnancy using insecticide-treated bednets and sulfadoxine-pyrimethamine in a highly malarious area of Kenya: a randomized controlled trial." Trans R Soc Trop Med Hyg **97**(3): 277-82.

Rogerson, S. J., Mkundika, P. and Kanjala, M. K. (2003). "Diagnosis of Plasmodium falciparum malaria at delivery: comparison of blood film preparation methods and of blood films with histology." J Clin Microbiol **41**: 1370 - 1374.

Romagosa, C., Menendez, C., Ismail, M. R., Quinto, L., Ferrer, B., Alonso, P. L. and Ordi, J. (2004). "Polarisation microscopy increases the sensitivity of hemozoin and Plasmodium detection in the histological assessment of placental malaria." Acta Trop **90**(3): 277-84.

Singer, L. M., Newman, R. D., Diarra, A., Moran, A. C., Huber, C. S., Stennies, G., Sirima, S. B., Konate, A., Yameogo, M., Sawadogo, R., Barnwell, J. W. and Parise, M. E. (2004). "Evaluation of a malaria rapid diagnostic test for assessing the burden of malaria during pregnancy." Am J Trop Med Hyg **70**(5): 481-5.

Singh, N., Saxena, A., Awadhia, S. B., Shrivastava, R. and Singh, M. P. (2005). "Evaluation of a rapid diagnostic test for assessing the burden of malaria at delivery in India." Am J Trop Med Hyg **73**(5): 855-8.

Tagbor, H., Bruce, J., Greenwood, B. and Chandramohan, D. (2008). "Performance of the OptiMAL® dipstick in the diagnosis of malaria infection in pregnancy." Therapeutics and Clinical Risk Management **4**(3): 631 - 636.

ter Kuile, F. O., van Eijk, A. M. and Filler, S. J. (2007). "Effect of sulfadoxine-pyrimethamine resistance on the efficacy of intermittent preventive therapy for malaria control during pregnancy: a systematic review." JAMA **297**(23): 2603-16.

VanderJagt, T. A., Ikeh, E. I., Ujah, I. O., Belmonte, J., Glew, R. H. and VanderJagt, D. J. (2005). "Comparison of the OptiMAL rapid test and microscopy for detection of malaria in pregnant women in Nigeria." Trop Med Int Health **10**(1): 39-41.

WHO (2008). Malaria rapid diagnostic test performance: results of WHO product testing of malaria RDTs: Round 1. Geneva, WHO.

Figure 1: - Flow chart of proposed study design

**Visit 5: Delivery / pregnancy outcome assessment**

Record all BW and foetal outcomes including abortions and stillbirths

**Visit 6: Post natal follow up**

Follow-up mother and child at 6 to 8 weeks

Check blood slide of the mother for MP.

**Randomise eligible pregnant women into study arms at ANC sessions.**

**Visits: 2, 3 & 4**

Passive follow up at 3 scheduled visits and repeat SP-IPTp at ANC.

**Visit 1: Enrolment**

Provide standard SP-IPTp. Provide LLINs and daily doses of haematinics.

**Visit 1: Enrolment**

RDT screening and treatment of RDT positive cases only. Provide LLINs and daily doses of haematinics.

**Visits: 2, 3 & 4**

Passive follow up at 3 scheduled visits. Screen and treat if parasitaemic at ANC.

**Visit 5: Delivery / pregnancy outcome assessment**

Record all BW and foetal outcomes including abortions and stillbirths

**Visit 6: Post natal follow up**

Follow-up mother and child at 6 to 8 weeks

Check blood slide of the mother for MP.

APPENDIX

## Participant information sheet and consent form

**Title:** Evaluation of methods for the prevention of malaria in pregnancy in the context of high coverage with insecticide treated nets and declining, seasonal malaria transmission.

**Investigators:** Local PIs

The following will translated into appropriate local languages.

Malaria in pregnancy is life threatening to both the mother and the baby she is carrying. It can result in the destruction of the mother’s blood and in babies born weighing less than normal, making them less healthy in their first years of life. Over the years, effective measures have been taken to control the harmful effects of malaria in pregnancy and this has resulted in less pregnant women suffering from malaria than previously in our country, as in many parts of Africa. Scientists now think it is time to find out whether one of these measures called intermittent preventive treatment (IPTp), which requires that pregnant women are given a malaria drug called SP on several occasions during pregnancy, is still useful particularly in areas like ours where pregnant women are at risk for only a short period of the year.

We in Burkina Faso, Ghana, Mali, and the Gambia wish to test this idea in two groups of pregnant women. One group will have a blood test for malaria and treatment of anyone who is positive at scheduled antenatal clinic visits; the second group of pregnant women will be given the drugs two or more times as is currently done. When women in the two groups deliver we will compare the strength of their blood and the weight of their babies and take a sample of the placenta to see if this has been infected with malaria. This will help us to know which strategy is more effective in protecting pregnant women from having low birth weight babies and anaemia due to malaria infection. We would like you to help us do this by participating in the study. If you agree to participate in this study, you will be one of 5000 women in Ghana, Burkina Faso, The Gambia and Mali also participating in the study. The findings will help the Ministry of Health to better control malaria in pregnancy.

If you agree to participate in this study, this is what this will involve.

1. We will ask you some questions about yourself including your general health, your education level and living conditions.
2. We will take a little blood by a prick of your fingertip to test for malaria and thinning of blood to help us know your health status at the beginning of the study. The blood samples will not be used for any other purpose without your permission.
3. You will be allocated to one of the two options by lottery. You will receive a long-lasting insecticide treated bed net and will be expected to use it throughout your pregnancy. We will visit your home at random to find out whether you are using it.
4. If we find malaria parasites in your blood you will be given treatment. You will be required to return to the clinic on dates we give you.
5. If you are included in the group that requires us to test you for malaria and treat if this is found on the blood test, we will do this on the dates we give you. If you are allocated to the group to receive treatment as is currently practiced you will be given the next dose of SP.
6. We will ask you to deliver at hospital so that we can examine and weigh your baby, check the thinning of your blood and obtain a sample of your placenta. However, if you are unable to deliver at hospital please notify us through a member of your community health committee so that we can visit you at home and do the necessary tests on you and your baby as soon as possible after it is born.
7. When your baby is 40 days old we will visit you to find out how you and the baby are doing and take blood by finger prick to test for malaria parasite and thinning of blood. This visit marks the completion of your participation in this study.

From today until the time you deliver you will not be required to pay for any treatment you receive as part of this study. This includes other visits you may make for other illnesses on days other than those we gave you. Every medicine including those for treating malaria and strengthening your blood will be free. There will be someone at the clinic to attend to you anytime you come in between scheduled visits.

Any information we have about you will not be disclosed to anyone by us. Participation in this study is voluntary. If you do not wish to participate in the study, it will not affect the regular attention you have been receiving from this hospital/health centre now or in the future. You may withdraw from the study at any time without this affecting the care that you receive at the antenatal clinic.

We are happy to answer any questions you may have that will help you decide whether or not you want to take part in the study, or to explain further what taking part in this study will mean for you and your baby.

**CONSENT:**

I have been adequately informed of (I have read and understood) the purpose, procedures, potential risks and benefits of this study. I have had the opportunity to ask questions about the study. Any questions that I have asked have been answered to my satisfaction. I know that I can refuse to participate in this study without any loss of benefit to which I would have otherwise been entitled. I understand that if I agree to participate, I can withdraw my consent at any time without losing any benefits or services to which I am entitled. I understand that any information collected will be treated confidentially. I understand that agreeing to participate and signing this form does not necessarily mean I will be able to participate in the study because I will be assessed for eligibility before enrolment.

I freely agree to participate in the study. After signing below, I will receive a copy each of the information sheet and the consent form.

**Name of participant**: ……………………………………………………………………………….

Signature or Right Thumb Print: …………………

Date: -----/-----/-----

**Name of witness:** …………………………………………………………………………………..

Signature or Right Thumb Print: …………………..

Date: -----/-----/-----

I have adequately informed the participant of the purpose, procedures, potential risks and benefits of this study. I have answered all questions to the best of my ability.

**Name of study personnel:** ……………………………………………..

Signature: ……………………………………

Date: -------/------/------

## Tables

Table 1: Background information on the study sites or nearby areas

| **Parameter** | **Burkina Faso** | **Ghana** | **Mali** | **The Gambia** |
| --- | --- | --- | --- | --- |
| 1. **EIR (bites/person/year)** | 100 – 300 | 200 | 19.23 – 21.1 during rainy season | 10 – 50 |
| 1. **Malaria prevalence** |  |  |  |  |
| **Children <5 years** | 53.5% | 41.5% | 39.5% | 20% |
| 1. **Antenatal** |  |  |  |  |
| All gravidae | 20 – 25% | 47% | 23.9% | 8% |
| Primigravidae | 25% | 58% | 62.1% | 28% |
| 1. **Placenta** |  |  |  |  |
| All gravidae | 22% | 32% | 17.1 – 42.3% | No recent data |
| Primigravidae |  |  | 8.8% |  |
| 1. **ANC attendance** | >90% | 98% | >90% | 98% |
| 1. **SP-IPTp2 or more coverage** | Not available | 35% | 4% | 32% |
| 1. **Antimalarial drug policy** |  |  |  |  |
| 1^st^ line | Quinine | Quinine | Quinine | Quinine |
| 2^nd^ line | AQ/AS; AL | AQ/AS; AL | AQ/AS; AL | AQ/AS; AL |
| 1. **Folic acid supplementation dose** | 0.25mg | 5mg | 5mg | 5mg |
| 1. **Iron supplementation dose** | 200mg | 200mg | 200mg | 200mg |
| 1. **HIV control policy** |  |  |  |  |
| HIV prevalence among pregnant women | 1.6% | 2.8% | Low | 2.8% |
| HIV screening at ANC | VCT | VCT | VCT | VCT |
| ART policy | Refer to ART centre for drugs | Refer to ART centre for drugs | Refer to ART centre for drugs | Refer to ART centre for drugs |
| 1. **ITN coverage / use among pregnant women** | Not available | 40 – 52.5% | 29% | 50% |
| 1. **SP resistance** | 10 – 15% | 8% | 10 – 15% | 15% |
| % triple dhfr mutations | Not available | 73% | 25% | 30% |
| % quadruple mutatons (triple dhfr plus 1 dhps ) |  | Not available | 17% | Not available |
| % quintuple mutations (triple dhfr plus double dhps ) |  |  | 0% |  |

le 2: - Time scale of planned activities

|  | | **ACTIVITY DESCRIPTION** | **ACTIVITY YEAR & MONTH** | | | | | | | | | | | | | | | | | | | | | |
| --- | --- | --- | --- | --- | --- | --- | --- | --- | --- | --- | --- | --- | --- | --- | --- | --- | --- | --- | --- | --- | --- | --- | --- | --- |
|  |  |  | 2008 | | 2009 | | | | 2010 | | | | 2011 | | | | 2012 | | | | 2013 | | | |
| **Goal** |  | **To reduce the burden of malaria in pregnant women** | Q3 | Q4 | Q1 | Q2 | Q3 | Q4 | Q1 | Q2 | Q3 | Q4 | Q1 | Q2 | Q3 | Q4 | Q1 | Q2 | Q3 | Q4 | Q1 | Q2 | Q3 | Q4 |
| **Objective 1:** |  | To determine whether in areas of seasonal malaria transmission, intermittent preventive treatment (IPTp) with sulphadoxine/pyrimethamine (SP) can be replaced safely and effectively with screening and treatment of pregnant women who are infected. |  |  |  |  |  |  |  |  |  |  |  |  |  |  |  |  |  |  |  |  |  |  |
| **Activity 1** |  | Coordination of a research program on the prevention of malaria in pregnancy at centres in four countries in West Africa. |  |  |  |  |  |  |  |  |  |  |  |  |  |  |  |  |  |  |  |  |  |  |
| **Milestones** | 1 | Recruitment of a trial coordinator |  |  |  |  |  |  |  |  |  |  |  |  |  |  |  |  |  |  |  |  |  |  |
|  | 2 | Start-up meeting of trial investigators |  |  |  |  |  |  |  |  |  |  |  |  |  |  |  |  |  |  |  |  |  |  |
|  | 3 | Appointment of a Data and Safety Management Board (DSMB) |  |  |  |  |  |  |  |  |  |  |  |  |  |  |  |  |  |  |  |  |  |  |
|  | 4 | Appointment of clinical/safety monitor |  |  |  |  |  |  |  |  |  |  |  |  |  |  |  |  |  |  |  |  |  |  |
|  | 5 | Organisation of annual PIs meetings |  |  |  |  |  |  |  |  |  |  |  |  |  |  |  |  |  |  |  |  |  |  |
|  | 6 | Preparation of annual progress reports |  |  |  |  |  |  |  |  |  |  |  |  |  |  |  |  |  |  |  |  |  |  |
|  | 7 | Preparation of final report |  |  |  |  |  |  |  |  |  |  |  |  |  |  |  |  |  |  |  |  |  |  |
|  | 8 | Submission of publications |  |  |  |  |  |  |  |  |  |  |  |  |  |  |  |  |  |  |  |  |  |  |
| **Activity 2** |  | Conducting a trial of the ability of sulphadoxine/pyriemthamine (SP) to clear *Plasmodium falciparum* parasitemia in pregnant women in two countries in West Africa. | Q3 | Q4 | Q1 | Q2 | Q3 | Q4 | Q1 | Q2 | Q3 | Q4 | Q1 | Q2 | Q3 | Q4 | Q1 | Q2 | Q3 | Q4 | Q1 | Q2 | Q3 | Q4 |
| **Milestones** | 9 | Obtain ethical clearance for the study at all centres |  |  |  |  |  |  |  |  |  |  |  |  |  |  |  |  |  |  |  |  |  |  |
|  | 10 | Start the study |  |  |  |  |  |  |  |  |  |  |  |  |  |  |  |  |  |  |  |  |  |  |
|  | 11 | Complete the study |  |  |  |  |  |  |  |  |  |  |  |  |  |  |  |  |  |  |  |  |  |  |
|  | 12 | Complete laboratory analyses |  |  |  |  |  |  |  |  |  |  |  |  |  |  |  |  |  |  |  |  |  |  |
|  | 13 | Complete data analysis and preparation for publication |  |  |  |  |  |  |  |  |  |  |  |  |  |  |  |  |  |  |  |  |  |  |
| **Activity 3** |  | Conducting a randomised trial to compare intermittent preventive treatment for malaria in pregnancy (IPTp) with SP and screening and treatment of infected women as approaches to the control of malaria in pregnancy in four countries in West Africa. | Q3 | Q4 | Q1 | Q2 | Q3 | Q4 | Q1 | Q2 | Q3 | Q4 | Q1 | Q2 | Q3 | Q4 | Q1 | Q2 | Q3 | Q4 | Q1 | Q2 | Q3 | Q4 |
|  | 14 | Revision of protocol; preparation of CRFs and SOPS |  |  |  |  |  |  |  |  |  |  |  |  |  |  |  |  |  |  |  |  |  |  |
|  | 15 | Protocol revisions recommended by EX COMM |  |  |  |  |  |  |  |  |  |  |  |  |  |  |  |  |  |  |  |  |  |  |
| **Milestones** | 16 | Final approval from MIP consortium Executive Committee |  |  |  |  |  |  |  |  |  |  |  |  |  |  |  |  |  |  |  |  |  |  |
|  | 17 | Ethical and regulatory approvals obtained |  |  |  |  |  |  |  |  |  |  |  |  |  |  |  |  |  |  |  |  |  |  |
|  | 18 | Recruitment started |  |  |  |  |  |  |  |  |  |  |  |  |  |  |  |  |  |  |  |  |  |  |
|  | 19 | Recruitment completed |  |  |  |  |  |  |  |  |  |  |  |  |  |  |  |  |  |  |  |  |  |  |
|  | 20 | Follow-up completed |  |  |  |  |  |  |  |  |  |  |  |  |  |  |  |  |  |  |  |  |  |  |
|  | 21 | Laboratory analyses completed |  |  |  |  |  |  |  |  |  |  |  |  |  |  |  |  |  |  |  |  |  |  |
|  | 22 | Complete data analysis and preparations for publication |  |  |  |  |  |  |  |  |  |  |  |  |  |  |  |  |  |  |  |  |  |  |
