# Supplementary material for: A Non-Inferiority, Individually Randomized Trial of Intermittent Screening and Treatment versus Intermittent Preventive Treatment in the Control of Malaria in Pregnancy
Source: PLoS One. 2015 Aug 10;10(8):e0132247. doi: 10.1371/journal.pone.0132247 (PMC4530893; doi:10.1371/journal.pone.0132247)
Supplement: S3 Text — (DOCX) [file pone.0132247.s022.docx]

# S3 Text

# Amendments to Trial protocol

**Amendment 1**

**London School of Hygiene & Tropical Medicine E2**

| **Amendment Number** | **1** |
| --- | --- |

Keppel Street, London WC1E 7HT

**Ethics Committee**

**Application to amend or extend a study which has**

**received LSHTM Ethics Committee approval**

| Title of Project  Intermittent preventive treatment with sulphadoxine-pyrimethamine versus intermittent screening and treatment of malaria in pregnancy | |
| --- | --- |
| LSHTM Ethics Committee  Reference number | 5545 |
| Date approved |  |
| Name of Principal Investigator (PI)  at LSHTM | Prof. Brian Greenwood |
| Appointment Held (or Research student) |  |
| Unit/Department | DCVBU/ITD |
| Signature of Principal Investigator |  |
| *If Research student:*  Name, signature and approval of Supervisor |  |
| Does this study involve the taking of tissue and/or blood samples | YES |

| Received by Committee: |  |
| --- | --- |
| Approved by Chair: |  |
| Date: |  |

|  | Give an outline of the proposed amendment/extension.  Sufficient detail must be given to allow the Committee to make an informed decision. |
| --- | --- |
|  | The ethics committee in August, 2009, approved a proposed project entitled “intermittent preventive treatment with sulphadoxine-pyrimethamine versus intermittent screening and treatment of malaria in pregnancy”. The goal of this project is to determine whether in pregnant women who sleep under a long lasting insecticide treated bed net, screening and treatment at each scheduled antenatal clinic visit is as effective in protecting them from anaemia, low birth weight and placental infection as SP-IPTp.  Following a review by the Malaria in Pregnancy Executive Committee and Trial Monitor and, following discussions during a meeting of investigating teams and the coordinators held in Ouagadougou, Burkina Faso on 16^th^ and 17^th^ February 2010 and in Dar es Salaam on 18^th^ June 2010 a few modifications to the original protocol have been proposed proposed. The relevant modifications relate to the following:  ***Iron and folate policies*:** The earlier versions of the protocol had the dosage of folic acid as 5 mg but this is now changed to 0.4mg daily taking into account the variations in national guidelines. The dosage of ferrous sulphate remains 200 mg daily.  ***Management of symptomatic women between scheduled visits*:** Women who present with fever between screening visits and who are RDT or blood film positive will be treated with Coartem® instead of quinine, in line with national guidelines which allow the use of registered Artemisinin combination therapy (ACTs) in the second and third trimester of pregnancy.  ***Detecting infections between visits*:** We now will -   1. Obtain blood samples from all study women for the determination of baseline parasitaemia by microscopy and PCR. 2. Obtain additional blood samples from all study women for thin and thick blood films for malaria parasite counts and filter paper blood spots for molecular studies during scheduled visits. Figure 1 has been updated accordingly.   ***Change of data management system:*** We will use Teleform (Cardiff Software Inc., Vista CA); an optical character recognition-based data management system that scans paper case report forms (CRF) and exports data to a computer database to avoid the need for double entry, as used in the traditional data management system.  ***Determination of gestational age at birth:*** We will do Ballard scoring on all newborns to estimate their gestational ages. Figure 2 provides a graphic way of doing it.  ***Clinical malaria at enrolment:*** it is now decided that if an eligible woman has objective fever (axillary temperature of 37.5^o^C or more) and signs and symptoms of malaria at enrolment she will be randomised into any of the study arms as any other woman. She will be screened with RDT and if positive treated with Coartem®. If she belongs to the IST arm this constitute an intervention but if she belongs to the IPT arm she will take the appropriate intervention at the next scheduled visit. If the RDT is negative she will receive SP if she belongs to the IPT arm but no antimalarial if belongs to the IST arm.  ***Assessment of adverse events:*** instead of actively following on day 7 to record adverse events we will now encourage study women report any untoward medical occurrence they may experience following ingestion of an antimalarial drug including occurrences which are not necessarily caused by or related to the antimalarial.  ***Frequency of determination of Hb:*** Haemoglobin will now be determined three times for each woman; first at recruitment, then at 38 weeks (+/-2 weeks) and at the time of delivery or shortly afterwards. The schedule of procedures in Table 1 has been updated accordingly. In the original protocol haemoglobin was measured five times for each woman. |
|  | Does this amendment/extension involve the taking of blood samples and/or other tissue? |
|  | Yes – additional finger prick blood samples will be obtained also from women in the IPTp arm for parasitaemia by microscopy and PCR during scheduled visits. In the original protocol, these samples were to be obtained from women in the screening and treatment arm only, during scheduled visits. This amendment will enable the determination of rate of infection among the study women during follow up. |
| 2.1 | If YES – Does this change arrangements already notified in the original application? |
|  | See above and attached table. |
| 2.2 | If YES - List samples which will be taken |
|  | See above |
| 2.3 | Please confirm that you have undertaken the on-line training programme available at  <http://intra.lshtm.ac.uk/support/research/humantissueact.html> and that you will ensure that any staff involved in the procedures for taking consent will also have undertaken an agreed training programme. |
|  | YES |
| 2.4 | If samples are taken overseas, will the samples be brought back to the UK? |
|  | NO |

**Amendment 2**

**London School of Hygiene & Tropical Medicine E2**

Aug 2010

| **Amendment Number** | **2** |
| --- | --- |

Keppel Street, London WC1E 7HT

**Ethics Committee**

**Application to amend or extend a study which has**

**received LSHTM Ethics Committee approval**

| Title of Project  Intermittent preventive treatment with sulfadoxine-pyrimethamine versus intermittent screening and treatment of malaria in pregnancy. | |
| --- | --- |
| LSHTM Ethics Committee  Reference number | 5545 |
| Date approved | 22.7.09 |
| Name of Principal Investigator (PI)  at LSHTM | Prof. Brian Greenwood |
| Appointment Held (or Research student) | Professor of Tropical Medicine |
| Unit/Department | Disease Control/Infectious and Tropical Diseases |
| Signature of Principal Investigator | 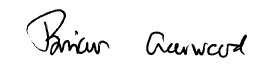 |
| *If Research student:*  Name, signature and approval of Supervisor |  |
| Does this study involve the taking of tissue and/or blood samples | YES |

| Received by Committee: |  |
| --- | --- |
| Approved by Chair: |  |
| Date: |  |

|  | Summary of the objectives, methods and other main features of the original study.  Please ensure that you do not exceed a maximum of 300 words. |
| --- | --- |
|  | The overall goal of this project is to determine whether screening pregnant women who sleep under a long lasting insecticide treated bed net for malaria, using a rapid diagnostic test (RDT) at each scheduled antenatal clinic visit, and treatment of those who are positive is as effective in protecting them from low birth weight and anaemia as intermittent preventive treatment (IPT) with sulphadoxine pyrimethamine (SP).  Primigravidae and secundigravidae who present at antenatal clinics in study sites in four West African countries (Burkina Faso, Ghana, Mali and The Gambia) are being randomised to one of two groups. All women are given a long lasting insecticide treated bed net on recruitment. Women in group 1 (reference group) receive SP according to the current WHO guidelines. Those in group 2 are screened with a rapid diagnostic test at each scheduled antenatal clinic visit and treated if parasitaemic. The primary end points of the trial are birth weight and anaemia at 36 - 40 weeks of gestation and at the time of delivery or shortly afterwards. The presence of placental malaria is also being determined. The study will recruit 5000 women, 2500 in each group and it is powered to show non-inferiority of group 2 compared to group 1. Women are encouraged to deliver in hospital where maternal haemoglobin and birth weight are recorded and a placental sample obtained for histology. Those who deliver at home are visited within a week of delivery and maternal haemoglobin and infant weight recorded. Mothers and infants are seen again six weeks after delivery. The costs and cost effectiveness of each intervention are being evaluated. |
| 2 | Summary of the specific amendment/extension requested.  Sufficient detail must be given to allow the Committee to make an informed decision |
|  | Following a review meeting of investigating teams and coordinators, held in Accra, Ghana on 29^th^ and 30^th^ November 2010, a decision was taken to amend the study protocol to allow the gestational age for inclusion in the study to be extended as it was considered that women who might benefit from inclusion in the study were being excluded unnecessarily. In addition, two other minor errors in the protocol were detected and corrected. Details are as follows:  ***Gestational age for inclusion:*** An inclusion criterion in the original protocol was that a woman had to be 16 to 24 weeks pregnant. Permission is requested to change this to 16 to 30 weeks. This has become necessary because experience has shown that many women in the study areas are reporting to antenatal clinics for the first time after 24 weeks of gestation and are therefore not eligible for inclusion in the study. Extending the eligible gestational age to 30 weeks will allow an additional group of women in the IPT arm the opportunity to receive at least two doses of SP and those in the IST arm the opportunity to attend two screening and treatment sessions and thus to contribute to determining which of these approaches to malaria control in pregnancy is most effective.  ***Corrections*:** Earlier reviews recommended omission of blood sampling for maternal Hb measurement at postnatal follow-up visits. Although this change was effected in the SOPs and the “schedule of procedures at follow up visits” (table 1) of the protocol, the relevant section of the text was not deleted from the protocol. This has now been corrected. Similarly, omission of sampling of cord blood was earlier recommended but the relevant text was not deleted. This has now been done. |
| 3 | Does this amendment/extension involve the taking of blood samples and/or other tissue? |
|  | The amendment does not involve the collection of any additional samples in addition to those approved in the original protocol. |
| 3.1 | If YES – Does this change arrangements already notified in the original application? |
|  | No. |
| 3.2 | If YES - List samples which will be taken |
|  | NA. |
| 3.3 | Please confirm that you have undertaken the on-line training programme available at  <http://intra.lshtm.ac.uk/support/research/humantissueact.html> and that you will ensure that any staff involved in the procedures for taking consent will also have undertaken an agreed training programme. |
|  | Yes. |
| 3.4 | If samples are taken overseas, will the samples be brought back to the UK? |
|  | No. |

**Amendment 3**

**London School of Hygiene & Tropical Medicine E2**

Aug 2010

| **Amendment Number** | **3** |
| --- | --- |

Keppel Street, London WC1E 7HT

**Ethics Committee**

**Application to amend or extend a study which has**

**received LSHTM Ethics Committee approval**

| Title of Project  Intermittent preventive treatment with sulfadoxine-pyrimethamine versus intermittent screening and treatment of malaria in pregnancy. | |
| --- | --- |
| LSHTM Ethics Committee  Reference number | 5545 |
| Date approved |  |
| Name of Principal Investigator (PI)  at LSHTM | Prof. Brian Greenwood |
| Appointment Held (or Research student) | Professor of tropical Medicine |
| Unit/Department | DCVBU/ITD |
| Signature of Principal Investigator | 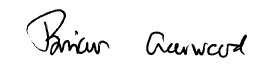 |
| *If Research student:*  Name, signature and approval of Supervisor |  |
| Does this study involve the taking of tissue and/or blood samples | YES |

| Received by Committee: |  |
| --- | --- |
| Approved by Chair: |  |
| Date: |  |

|  | Summary of the objectives, methods and other main features of the original study.  Please ensure that you do not exceed a maximum of 300 words. |
| --- | --- |
|  | The overall goal of this project is to determine whether in pregnant women who sleep under a long lasting insecticide treated bed net, screening and treatment at each scheduled antenatal clinic visit is as effective in protecting them from low birth weight and anaemia as WHO recommended intermittent preventive treatment with sulkphadoxine-pyrimethamine (SP) .  Primigravidae and secundigravidae who present at antenatal clinics in study sites in four West African countries (Burkina Faso, Ghana, Mali and The Gambia) are being randomised to one of two groups. All women are given a long lasting insecticide treated bed net. Women in group 1 (reference group) receive SP according to the current WHO guidelines. Those in group 2 are screened with a rapid diagnostic test at each scheduled antenatal clinic visit and treated if parasitaemic. The primary end points of the trial are birth weight, placenta malaria by histology and anaemia at 36 – 40 weeks of gestation and at the time of delivery or shortly afterwards.. Women are encouraged to deliver in hospital where maternal haemoglobin and birth weight are recorded. Those who deliver at home are visited within a week of delivery and maternal haemoglobin and infant weight recorded. Mothers and infants are seen again six weeks after delivery. The costs and cost effectiveness of each intervention are being evaluated.  Recruitment started in July 2010 and so far approximately 2090 women have been enrolled and 648 delivered. The target sample size is 5,000 with approximately 1,250 being recruited at each site. Enrolment will finish around July 2011 and follow-up at the end of the year. |
| 2 | Summary of the specific amendment/extension requested.  Sufficient detail must be given to allow the Committee to make an informed decision |
|  | Malaria diagnosis at delivery was originally proposed to be done by microscopy and PCR using both peripheral maternal blood and placental blood. However, following a recent suggestion by the trial coordinating team we would like determine the efficacy of a malaria rapid diagnostic test (RDT) in the diagnosis of placenta malaria as a possible alternative to much more demanding histological examination of the placenta which is being undertaken in this clinical trial but could not be done routinely. In this regard the following modification has been made in the current protocol (***MA05 Final Protocol: 22 June, 2010***) and we wish to inform the ethics committee of this small change.  ***Malaria diagnosis at delivery:*** This will be done using both placental and peripheral blood samples by microscopy, RDT, and PCR. During labour a finger prick sample of peripheral blood will be collected from pregnant women for thick and thin blood smears, filter paper blood spot and rapid diagnostic testing using RDTs. After delivery and following expulsion of placenta, the placenta will gently be cleaned with running water to remove any mucus and maternal blood from its surface, and then placed on a flat surface with the maternal side up. A small incision will be made into the maternal surface of a healthy paracentric area of the placenta to collect blood from the intervillous space with a syringe containing EDTA for examination by blood film and RDT. |
| 3 | Does this amendment/extension involve the taking of blood samples and/or other tissue? |
|  | No additional samples will be collected – provision for collection of a small peripheral blood and a placental sample at delivery was included in the original submission. |
| 3.1 | If YES – Does this change arrangements already notified in the original application? |
|  | Not applicable. |
| 3.2 | If YES - List samples which will be taken |
|  | No additional samples will be obtained. |
| 3.3 | Please confirm that you have undertaken the on-line training programme available at  <http://intra.lshtm.ac.uk/support/research/humantissueact.html> and that you will ensure that any staff involved in the procedures for taking consent will also have undertaken an agreed training programme. |
|  | Yes |
| 3.4 | If samples are taken overseas, will the samples be brought back to the UK? |
|  | No |

**Amendment 4**

**[NOTE: This amendment relates to a follow-up of infants in the Ghana site. The results of this follow-up which are not yet fully analysed and not referred to in the current paper. The will be reported in a separate publication in due course]**

**London School of Hygiene & Tropical Medicine E2**

Aug 2010

| **Amendment Number** | **4** |
| --- | --- |

Keppel Street, London WC1E 7HT

**Ethics Committee**

**Application to amend or extend a study which has**

**received LSHTM Ethics Committee approval**

| Title of Project  Intermittent preventive treatment with sulfadoxine-pyrimethamine versus intermittent screening and treatment of malaria in pregnancy. | |
| --- | --- |
| LSHTM Ethics Committee  Reference number | 5545 |
| Date approved |  |
| Name of Principal Investigator (PI)  at LSHTM | Prof. Brian Greenwood |
| Appointment Held (or Research student) | Professor of Tropical Medicine |
| Unit/Department | DCVBU/ITD |
| Signature of Principal Investigator | 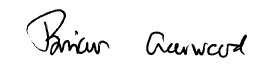 |
| *If Research student:*  Name, signature and approval of Supervisor |  |
| Does this study involve the taking of tissue and/or blood samples | YES |

| Received by Committee: |  |
| --- | --- |
| Approved by Chair: |  |
| Date: |  |

|  | Summary of the objectives, methods and other main features of the original study.  Please ensure that you do not exceed a maximum of 300 words. |
| --- | --- |
|  | The overall goal of this project is to determine whether in pregnant women who sleep under a long lasting insecticide treated bed net, screening for malaria and treatment of women who are positive at each scheduled antenatal clinic visit (IST) is as effective in protecting them from low birth weight and anaemia as the WHO recommended policy of intermittent preventive treatment with sulphadoxine-pyrimethamine (SP-IPTp).  Primigravidae and secundigravidae who have presented to antenatal clinics in study sites in four West African countries (Burkina Faso, Ghana, Mali and The Gambia) have been being randomised to one of two groups. All women have been given a long lasting insecticide treated bed net. Women in group 1 (reference group) receive SP according to the current WHO guidelines. Those in group 2 are screened with a rapid diagnostic test at each scheduled antenatal clinic visit and treated if parasitaemic. The primary end points of the trial are birth weight, placenta malaria by histology and anaemia at 36 – 40 weeks of gestation and at the time of delivery or shortly afterwards. Women are encouraged to deliver in hospital where maternal haemoglobin and birth weight are recorded. Those who deliver at home are visited within a week of delivery and maternal haemoglobin and infant weight recorded. Mothers and infants are seen again six weeks after delivery. The costs and cost effectiveness of each intervention are being evaluated.  Recruitment started in July 2010 and was completed in October 2011 when over 5,000 women, the target sample size, had been recruited. Follow up in the main trial will continue up to June 2012. |
| 2 | Summary of the specific amendment/extension requested.  Sufficient detail must be given to allow the Committee to make an informed decision |
|  | Recent evidence suggests that malaria infection during pregnancy, particularly in the last trimester, negatively affects an infant’s health. Studies have reported that an infant’s chance of developing malaria in the first year of life may be doubled by clinical malaria episodes during pregnancy and quadrupled by placental malaria infection. Antimalarial interventions which influence the likelihood of malaria infection during pregnancy may, therefore, not only have an effect on the risk of low birth and child survival immediately after delivery but may also influence the risk of an infant suffering from a clinical attack of malaria throughout the first year of life. Thus, before a recommendation could be made to switch from IPTp to IST, health authorities would need to be convinced that this posed no extra risk to an infant as well as to his/her mother. The hypothesis underlying this ancillary study is, therefore, that IST will not be inferior to IPTp in protecting an infant from malaria during his/her first year of life.  Infants in the MA05 trial are currently being followed up to the age of six weeks. We now propose to follow these infants throughout the whole of their first 12-18 months of life, paying particular attention to their susceptibility to malaria. This additional study will be done in Navrongo, northern Ghana and will be the subject of a PhD thesis by Dr Oyakirome, a Malaria Capacity Development Consortium (MCDC) student based at the University of Science and Technology (KNUST), Kumasi, Ghana. This amendment has already received approval from the relevant ethical committees in Ghana.  Approximately 800 infants born to mothers who had received IST or IPTp during their pregnancy will be recruited to the study. The babies of women recruited at the beginning of the trial are now over 6 months old and thus ineligible for this ancillary study. All babies who have not yet reached the age of 6 months at the time that permission is obtained for the trial to start will be considered for inclusion in the study. It is estimated that this number will be about 800. Recruitment of 800 infants will provide a study which has 80% power to show that IST is not more than 20% inferior to IPTp with SP in protecting infants from clinical episodes of malaria.  After informed consent has been obtained each child will be followed for a period of one year. Information on the incidence of both symptomatic and asymptomatic malaria infections in these infants will be obtained in three ways.   1. Project staff will be based at each of eight major health centres in the area and will investigate any study child brought to one of these health centres. If the child has fever or other features suggestive of malaria, a rapid diagnostic test will be done and a blood film obtained. Children with malaria will be treated according to national guidelines with amodiaquine/artesunate. 2. Each child will be seen when aged 6 and 12 months old and a finger prick blood sample collected for preparation of a blood film, measurement of haemoglobin and preparation of a blood spot for subsequent serological studies which will be done at LSHTM. 3. Each child will be visited once on a random basis during the year of follow-up and a blood film obtained for preparation of a malaria blood film regardless of whether or not the child has any signs of illness.   The passive surveillance described in (a) will provide information on the risk of symptomatic malaria whilst the active surveillance described in (b) and (c) will provide information on the risk of malaria infection overall.  At present, placental samples are being collected from approximately 85% of study women. The babies of women for whom a placenta is not obtained will not be excluded from the study, as there will be other information obtained from blood films about their exposure to malaria in pregnancy. However, a sub-analysis will be undertaken in just the infants whose mothers have provided a placental sample.  Because of its nature this has not been a double blind trial. Thus, the principal investigator for the ancillary study will have information on the study group to which an infant belongs. However, field staff undertaking measurements in the field and laboratory staff reading blood films will be blind to the group to which the infant who provided the sample belonged. |
| 3 | Does this amendment/extension involve the taking of blood samples and/or other tissue? |
|  | This extension will involve collection of three finger prick blood samples from study infants during the course of the one year follow-up period. |
| 3.1 | If YES – Does this change arrangements already notified in the original application? |
|  | Yes |
| 3.2 | If YES - List samples which will be taken |
|  | Three finger prick blood samples will be obtained as described above. |
| 3.3 | Please confirm that you have undertaken the on-line training programme available at  <http://intra.lshtm.ac.uk/support/research/humantissueact.html> and that you will ensure that any staff involved in the procedures for taking consent will also have undertaken an agreed training programme. |
|  | Yes |
| 3.4 | If samples are taken overseas, will the samples be brought back to the UK? |
|  | Finger-prick blood spots will be brought to LSHTM for serological assays. |

Amendment 5

London School of Hygiene & Tropical Medicine E2

Keppel Street, London WC1E 7HT Oct 2011

Observational/Interventions Research Ethics Committee

Application to amend or extend a study which has

received LSHTM Ethics Committee approval

*Please ensure you download and complete the latest version of this form from the intranet:* [***http://intra.lshtm.ac.uk/management/committees/ethics/***](http://intra.lshtm.ac.uk/management/committees/ethics/)

*This form should be completed and emailed along with all relevant attachments to* [*ethics@lshtm.ac.uk*](mailto:ethics@lshtm.ac.uk)

| For use of Ethics Committee only | Amendment No. 5 | Date received |
| --- | --- | --- |
|  | Response deadline | Date approval notification |

| Title of Project  Intermittent preventive treatment with sulfadoxine-pyrimethamine versus intermittent screening and treatment of malaria in pregnancy | |
| --- | --- |
| LSHTM Ethics Committee  Reference number | 5545 |
| Date approved |  |
| Name of Principal Investigator (PI)  at LSHTM | Prof. Brian Greenwood |
| Appointment Held (or Research student) | Professor |
| Department/Faculty | DCVBU/ITD |
| *If Research student:*  Name, electronic signature and approval of Supervisor (or attach email approval) |  |
| Does this study involve the taking of human tissue and/or blood samples | YES |

Approval is required for any substantial amendment to a study. Amendments are changes made to a research study after a favourable ethical opinion or approval by a regulatory body has been given. They can be made to a protocol, other essential documentation or other aspects of a study’s arrangements. All research protocols should have a clear version number and date in order to maintain accurate records and audit trails. Any amendment to a research protocol should have a concordant amendment to the date and version number.

Further detail can be found in LSHTM/SOP/023 Amendments (UK) - <http://intra.lshtm.ac.uk/trials/sops/sopsinpdf/sop_023_amendments.pdf> - although written for Clinical Trials the general principles apply across observational and interventions studies.

|  | Summary of the objectives, methods and other main features of the original study.  Please ensure that you do not exceed a maximum of 300 words. |
| --- | --- |
|  | The overall goal of this project is to determine whether in pregnant women who sleep under a long lasting insecticide treated bed net, screening and treatment at each scheduled antenatal clinic visit is as effective in protecting them their babies from low birth weight and anaemia as WHO recommended SP-IPT.  Primigravidae and secundigravidae who presented at antenatal clinics in study sites in four West African countries (Burkina Faso, Ghana, Mali and The Gambia) were randomised to one of two groups. All women were given a long lasting insecticide treated bed net. Women in group 1 (reference group) received SP according to the current WHO guidelines (SP-IPT). Those in group 2 were screened with a rapid diagnostic test at each scheduled antenatal clinic visit and treated if parasitaemic (IST). The primary end points of the trial are birth weight, placenta malaria by histology and anaemia at 36 – 40 weeks of gestation and at the time of delivery or shortly afterwards. The study has recruited just over 5000 women, 2500 in each group, the target sample size and is powered to show non-inferiority of group 2 compared to group 1. Women have been encouraged to deliver in hospital where maternal haemoglobin and birth weight can be recorded and about three quarters have done so. Those who delivered at home have been visited within a week of delivery and maternal haemoglobin and infant weight recorded. Mothers and infants are seen again six weeks after delivery. Follow-up of women and their babies will be completed in about three months’ time. The costs and cost effectiveness of each intervention is being evaluated. |
| 2 | Summary of the specific amendment/extension requested.  Sufficient detail must be given to allow the Committee to make an informed decision  Please list the pages on which changes to the main protocol have occurred due to the proposed amendment  *Please also ensure, where relevant, details on changes to taking/storage of human tissue are provided* |
|  | We are requesting approval for an amendment to the study protocol that would allow us to express the non-inferiority criteria in terms of odds ratios instead of relative risk. The study size of 2500 women in each group was estimated to have 90% power at a 0.025% significance level to demonstrate that the prevalence of low birth weight among infants born to mothers in the IST group is not more than 3% above that of infants born to mothers in the SP-IPTp group. This was based on a review of the literature indicated that the prevalence of LBW (birth weight <2,500 g) in primigravidae or secundigravidae who received a full course of SP-IPTp was likely to be about 10% with some variation between sites. However, preliminary data indicates that the prevalence of low birth weight is variable and is likely to be higher than 10%, probably in the range 18% to 20%. Since the non-inferiority margin varies according to the prevalence of the outcome, expressing the non-inferiority margin in terms of the odds ratio is a practical way to maintain constant power for the study in this context. The non-inferiority margin is now amended as an odds ratio of 1.263. An OR of 1.263 equates to a risk difference of 3.71% at a prevalence of 18%, and a risk difference of 2.3% at the original prevalence of 10%. Thus, this approach is more conservative than the analysis planned originally.  A study with 2,500 women in each group would have similar power to show that the difference in Hb concentration between groups is less than 0.2g/dl. For placental malaria, a study of this size will have around 90 % power to show that the difference in the prevalence of placental malaria between groups is less than 5% assuming a prevalence of placental malaria in both groups of 25%. As suggested for low birth weight this can be re-expressed in terms of an odds ratio of 1.286 ([30/70] / [25/75]). This is equivalent to a risk difference of 5.53% at a prevalence of 30%, and 6.15% at a prevalence of 40%. We originally estimated that around 50% of women would deliver in a hospital or health centre and provide a placental biopsy. With a reduced sample size of 2500, the study would have had 80% power to show that the difference in placental malaria between groups is less than the non-inferiority margin defined above. However, in practice slightly more women have provided a placental sample, so the power should be greater than 80%.  The relevant sections of "MA05 Final Protocol: 22 June, 2010" (attached) amended are **modifications to the protocol (page 3 and 5) and sample size (page 27).** |
| 3 | Please attach the amended study documents to this amendment application, giving documentation name, version number and date in the table below. |
|  | \| Document Name \| Version \| Date \| \| --- \| --- \| --- \| \| MA05 Protocol \| Final \| 05/03/2012 \| |
| 4 | Please give details of other ethical/regulatory approval obtained or required to approve the proposed amendment. Please electronically append copies of local approval letter(s) where this has already been obtained.  Approval from the LSHTM Committee is dependent on these approvals having been received. |
|  |  |
