# Supplementary material for: A Non-Inferiority, Individually Randomized Trial of Intermittent Screening and Treatment versus Intermittent Preventive Treatment in the Control of Malaria in Pregnancy
Source: PLoS One. 2015 Aug 10;10(8):e0132247. doi: 10.1371/journal.pone.0132247 (PMC4530893; doi:10.1371/journal.pone.0132247)
Supplement: S4 Text — (DOCX) [file pone.0132247.s023.docx]

# S4 Text.Analysis Plan-Clinical Findings

**MA05: A trial of intermittent preventive treatment with sulfadoxine-pyrimethamine versus intermittent screening and treatment of malaria in pregnancy.**

**Statistical Analysis Plan**

**Sections**

Database

Timings

Endpoints

Adverse Events

ATP, ITT

Exclusions

Loss to follow-up, missing data

Trial profile

Non-inferiority margins

Pooled analysis of all sites

Covariates

Subgroups & interactions

Statistical methods

Tables and figures

**Database**

Prior to completion of the study the data base will be cleaned and merged ready for analysis.

Consistency and range checks will be undertaken blind to treatment allocation before merging in the intervention group. A final version of the database will be burned to CD and a copy sent to the DSMB.

**Timings**

Recruitment in the last of the four sites was completed in October 2011.

Clinical follow-up and placental histological examination will be completed by June 2012.

**Endpoints**

The trial protocol specified three co-primary endpoints: low birth weight, maternal haemoglobin / anaemia and prevalence of placental malaria. The sample size was based primarily on having 90% power to demonstrate non-inferiority with respect to prevalence of low birth weight (defined as a birth weight less than 2500g). Mean birth weight will also be formally compared as a secondary outcome. Non-inferiority with respect to anaemia will be based on comparison of mean haemoglobin (Hb) concentration; anaemia defined using a range of Hb thresholds will be considered as secondary outcomes. Prevalence of placental malaria will be based on placental histology in the sub-group of women who deliver in a health facility. The trial is powered to establish non-inferiority for each of the primary outcomes separately.

**Primary outcomes:**

Prevalence of low birth weight, defined as birth weight less than 2500 g.

Mean maternal Hb measured at 38 weeks ± 2 weeks.

Prevalence of placental malaria infection (past, chronic or acute phase) based on placental histology.

**Secondary Outcomes**

1. Mean birth weight will be formally compared using a non-inferiority margin (detailed below in section on NI margins), to aid interpretation of the primary outcome of prevalence of low birth weight.

The following secondary outcomes will be tabulated, but will not be used to formally investigate non-inferiority of IST with respect to IPTp:

1. Risk at 38 weeks (± 2 weeks) of

- anaemia defined as Hb<11 g/dL ;
- moderately severe anaemia defined as Hb <8 g/dL;
- severe anaemia defined as Hb < 5 g/dl.

1. Risk at the time of delivery of

- anaemia defined as Hb<11 g/dL ;
- moderately severe anaemia defined as Hb <8 g/dL;
- severe anaemia defined as Hb < 5 g/dl.

1. Prevalence of placental malaria using various diagnostic criteria (see section below).
2. The prevalence of peripheral blood parasitaemia

- at 38 weeks (+/- 2 weeks) of gestation
- at the time of delivery
- at the post-partum follow-up visit.

1. Incidence of clinical malaria during the course of the pregnancy.
2. Risk of serious adverse events in the mother.
3. Risk of occurrence of congenital abnormalities.
4. Risk of babies small for gestational age at delivery (as defined in Landis et al. ^23^)
5. Risk of pre-term births
6. Risk of perinatal mortality (still birth and early neonatal)
7. Risk of maternal mortality

**Composite endpoints:**

1. Risk of low birth weight, small for gestational age or pre-term birth
2. Risk of adverse outcome of pregnancy – abortions (<28 weeks gestation), still births (≥28 weeks) or neonatal death.
3. Risk of any adverse birth outcomes, including: low birth weight, pre-term delivery (defined as <37 weeks), small for gestational age, abortions, still births, early neonatal deaths (within 7 days), congenital abnormalities or any other SAE of the baby.

**Placental malaria detected by other methods**

The primary analysis will be based on positive placental histology. The categories of placental malaria - past, acute, chronic, or combined will be tabulated. Prevalence according to histology and other methods for detection of placental malaria will be determined:

Positive placental blood film independent of histology

Positive RDT of placental blood, independent of histology

- Positive placental blood film or RDT of placental blood independent of histology.

The sensitivity and specificity of placental blood films and RDTs in diagnosing placental malaria will be calculated.

**Adverse Events**

Adverse events were detected passively when reported by women enrolled in the study. We will compare the frequency of specific adverse events between intervention groups. The relationship of any SAEs in women in the IPTp to the administration of SP will be defined. The following will be tabulated:

- Serious AEs
- Unsolicited AEs
- Abortions
- Pre-specified key adverse events (Rash, Vomiting)

Incidence of adverse events will be analysed separately in each site, pooled across all sites and, as an exploratory analysis, pooled across the three sites using the same approach for IPTp (i.e. Burkina Faso, Gambia and Mali), discussed in detail below.

**ATP, ITT**

The primary aim of this study is to investigate non-inferiority of IST compared to IPTp with respect to low birth weight, maternal haemoglobin / anaemia and placental malaria. Because the aim is to establish non-inferiority, analysis according to protocol (ATP) is the primary analysis strategy. ATP may reduce the risk of falsely claiming non-inferiority when a new treatment is actually inferior (type I error), a potential problem with an intention to treat analysis ^24^. Modified ITT analysis (including all women with outcome data who received at least one treatment, analysed as randomized) will also be done and if there is disagreement between the two analyses reasons for the disagreement will be explored.

If the entire confidence interval for a particular primary outcome excludes unity (i.e. the odds ratio for low birth weight or placental malaria excludes 1, or the mean difference for Hb excludes 0) and is thus compatible with superiority of IST over SP-IPT, analysis of superiority will be done using 2-sided p-values, with the ITT analysis as primary.

The primary according to protocol population will be defined as women who:

1. received IPTp or were tested with an RDT on at least **two** occasions AND
2. have information recorded on the *specific primary endpoint under investigation*.

Depending on the outcome under investigation, this will be women who:

- provided a blood sample for anaemia at 38 weeks gestation ± 2 weeks
- had the birth weight of their child measured within 7 days of delivery
- provided a sample for placental histology (only available for women who delivered in study clinics).

Pre-delivery Hb concentration will be analysed separately as a secondary outcome.

For the ATP analysis, women will be analysed as treated rather than as randomised (if there are any errors in allocation, these will be included as treated; women who received a mixture of both interventions i.e. IPTp and IST will be excluded).

Women with documented receipt of SP as an unscheduled treatment from the clinic will be considered as a protocol violation and will be excluded, since this does not follow national guidelines. This applies to either the IPTp or IST arm. Use of additional antimalarials (e.g. for self-treatment) outside the context of the study will be documented and tabulated.

It is known that in three of the four study sites (Burkina, Gambia and Mali) a third course of IPTp was not given due to concerns about the safety of IPTp after 36 weeks of gestation. However, women were still screened on up to three occasions with an RDT and if found positive were treated. We will perform secondary according to protocol analyses separately for the three sites giving only two courses of IPTp, and Ghana, the site which gave three IPTp courses.

Finally we will perform a modified intention to treat analysis, including all women enrolled in the study, analysed as randomised. All women who were randomised, received at least one course of IPTp or were screened by RDT at least once, and have data on the primary outcome under consideration will be included. In practice, all women were treated with SP or screened at the first contact immediately after being randomised, so there should be only very minor differences between this analysis and analysis strictly as randomised.

It will give greater confidence in our conclusions if the results are consistent between the ATP and ITT analysis populations ^24^. If they differ, we will explore reasons for this difference.

**Exclusions**

The number of women screened, eligible and enrolled will be tabulated for each site. Reasons that women were not eligible will be tabulated. The number of women eligible but not enrolled in the study, with reasons, will also be tabulated.

**Loss to follow-up, missing data**

Numbers and timing of losses to follow-up, with reasons for exit from the study (where known) will be tabulated.

**Trial profile**

Numbers screened, enrolled, seen at Visits 1, 2, 3, at delivery and at post-partum follow-up will be tabulated. Reasons for exit from the study will be indicated. This information will also be displayed by trial site.

**Non-inferiority margins**

Inferiority margins have been considered for both the mean birth weight and for the percentage of births that were of low birth weight (<2.5kg). It was considered that if IST reduced mean birth weight by less than 50g compared to SP-IPT, it would be concluded that IST does not adversely affect birth weight. A reduction of 100g or more would be a matter of concern. Data from eight trials of IPTp or chemoprophylaxis in primigravidae were collated; in treated groups the mean birth weight ranged from 2709g to 3077g with an SD of 318g to 533g, and the percentage low birth weight ranged from 4% to 24%. In these trials, the percentage of low birth weights was generally lower than predicted from the mean and SD assuming a normal distribution. The proportion of births of low birth weight, rather than the mean birth weight was, therefore, specified as the primary endpoint for this trial. Sample size was determined in order to have 90% power to exclude a difference in prevalence greater than 3% between the two groups if the prevalence of low birth weight in the SP-IPT group is 10%. It is desirable to adapt the margin depending on the prevalence of the outcome in the trial and it has been recommended that this should be done in such a way that trial power is approximately constant ^25^. The use of the odds ratio is a practical solution to this problem ^1^. The use of the odds ratio does not preserve constant power exactly, but in this respect, the odds ratio is better than the relative risk.

Odds ratios will be used for the primary analysis of low birth weight. Originally the study was powered to exclude a difference greater than 3% between the groups in the prevalence of LBW, assuming prevalence of 10% in the comparator group. However, preliminary data indicates that the prevalence of low birth weight is variable and is likely to be higher than 10%, probably in the range 18% to 20%. Recalculation of the non-inferiority margin to preserve 90% power suggests that a margin of 3.7% should be used at a prevalence of 18% and a margin of 4% if prevalence is 20%. Because there is uncertainty in the prevalence of low birth weight, and because this may vary from site to site, the non-inferiority margin will be expressed in terms of the odds ratio. A difference of 4% if prevalence is 20%, a relative difference of 20%, can be expressed as an odds ratio of ([24/76] / [20/80]) = 1.263. This odds ratio can be used to express the non-inferiority margin independent of the prevalence in the comparator group. An OR of 1.263 equates to a risk difference of 3.71% at prevalence of 18%, and a risk difference of 2.3% at the original prevalence of 10%. In other words, this new criterion is more stringent than the one originally specified assuming a prevalence of 10%.

An additional and more stringent comparison will be undertaken using the continuous outcome of mean birth weight in the two groups. There is consensus that any difference in mean birth weight between the groups less than 50g would be acceptable, and this will therefore be calculated in addition to the primary outcome. The continuous outcome also has the advantage that it can be readily interpreted in the separate sites, regardless of any differences in the underlying prevalence of low birth weight.

The protocol specified the non-inferiority margin for Hb as 0.2 g / dL. This will be used for the primary analysis of maternal haemoglobin. Prevalence of anaemia using different thresholds will be analysed as a secondary endpoint.

Odds ratios will be used for the primary analysis of placental malaria. The non-inferiority margin for risk of placental malaria was specified as 5%, assuming a prevalence of placental malaria in both groups of 25%. As for LBW, this can be re-expressed in terms of the odds ratio of 1.286 ([30/70] / [25/75]). This is equivalent to a risk difference of 5.53% at prevalence of 30%, and 6.15% at a prevalence of 40%.

**Pooled analysis of all sites**

The primary analysis will be pooled across trial sites, irrespective of heterogeneity in the effect in different sites. As an exploratory analysis, interactions between intervention group and trial site will be explored, and reasons for the heterogeneity (such as possible differences in transmission intensity and SP resistance) will be investigated.

As discussed above, for some secondary analyses, it may be of interest to look at individual sites in detail and where appropriate, results will be tabulated by study site as well as pooled across centres.

**Covariates**

As an individually randomised clinical trial, confounders should be balanced between groups within sites. Distribution of confounding factors may be different between sites. Trial site will therefore be included as a covariate in the model.

Other covariates we will adjust for *a priori* (even if balanced by randomisation) are parity and, gestational age at enrolment (assessed by fundal height).

Data on socio-economic status and ITN use last night were obtained at enrolment. Exploratory analyses will be undertaken to consider the impact of adjusting for these covariates.

**Subgroups & interactions**

Exploratory analyses will be undertaken to investigate the following:

- Site specific analyses
- Odds ratios for LBW, mean differences for maternal haemoglobin, and odds ratios for PM by parity: primigravidae, secundigravidae.
- Differences in LBW restricted to those women who delivered between 36 and 40 weeks of gestation.
- LBW, maternal Hb, PM by broad age group: as increasing age and parity may independently provide women with greater immunity against malaria
- Interaction between age and parity, comparison of older secundigravid vs. young primigravid.
- Prevalence of pre-term births (based on Ballard score)
- Comparisons between the three primary endpoints will be performed stratified by the season in which IPTp / IST was received.
- Comparison of the effect of SP stratified by placental malaria status, to investigate the possible effect of SP on birth weight via its antibiotic effect.
- Sub-group analysis of women who were enrolled at the time of year that meant they were eligible to receive all their interventions during the transmission season (approximately June-November for most sites). Women who received any intervention outside the transmission season, or who would have been eligible to receive interventions at these times will be excluded.

**Statistical methods**

The odds ratio will be used for analysis of the prevalence of LBW and placental malaria. For binary outcomes, the 95% confidence interval for the OR will be calculated and compared to the pre-specified non-inferiority margin for each endpoint. 90%, 95% and 99% confidence intervals will be calculated. If the upper limit of the 95% confidence interval (IST:SP-IPT) for the odds ratio for LBW is less than 1.263, we will conclude non-inferiority (equivalent to a 1-sided test at the 2.5% level). If the upper limit of the 90% interval is less than the margin this will provide somewhat weaker evidence of non-inferiority. If the 99% intervals is less than this, this will give correspondingly greater confidence in the conclusion of non-inferiority.

The crude and covariate adjusted mean difference in maternal Hb will be calculated using normal (Gaussian) regression, again using a two-sided 95% CI (equivalent to a one-sided 97.5% CI).

For secondary outcomes for which a non-inferiority margin is not specified, crude mean differences, rate differences or risk differences (as appropriate to the outcome under consideration) will be calculated using standard methods. Covariate adjusted risk differences will be calculated using the least squares method described in Cheung et al ^26^.

**Assessment of effectiveness and cost-effectiveness of the interventions**

As a non-inferiority trial, it is important to establish that the comparator intervention (SP-IPTp) was effective. Outcomes in relation to number of courses of SP-IPTp will be compared with the aim of establishing that the intervention was effective. Molecular analyses of SP resistance markers from samples collected from study women, will also aid interpretation. A separate analytical plan for the molecular work on SP resistance will be developed.

An analysis plan to evaluate cost-effectiveness will also be developed separately.

**Tables and figures**

1. Classical consort chart with reasons for drop outs (Fig).
2. Comparison of base-line characteristics of women in the two groups (Table)
3. Contribution of each centre to the pooled analysis (Table).
4. Distribution of birth weights by group – figure, using kernel estimates of the density.
5. Numbers of LBW by group (Table)
6. Distribution of Hb concentration by group – figure, using kernel estimates of the density.
7. Numbers of women with anaemia and moderate anaemia by group (Table)
8. Prevalence of parasitaemia by group at different time points (Table)
9. Numbers of clinical episodes of malaria by group (Table)
10. Placental findings by group broken down by histological category (Table)
11. Secondary outcomes by group (Table)
12. Birth outcomes by group (Table)
13. Adverse events by group (Table)

**Example figure to explain interpretation of the trial:**


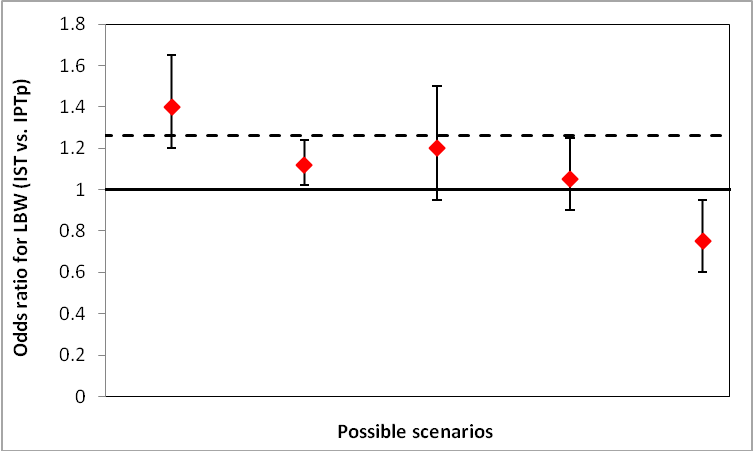


Figure shows Odds ratio for non-inferiority of IST versus IPTp with respect to low birth weight. Possible scenarios (from left to right), inferior, non-inferior (according to pre-specified margin), inconclusive, non-inferior, superior. Dotted line is the margin for non-inferiority of 1.263, equivalent to a risk difference of 4% if prevalence is 20%.

**References**

1. Landis SH, Ananth CV, Lokomba V, Hartmann KE, Thorp JM, Jr., Horton A, et al. Ultrasound-derived fetal size nomogram for a sub-Saharan African population: a longitudinal study. Ultrasound Obstet Gynecol. 2009 Oct;34(4):379-86.

2. Piaggio G, Elbourne DR, Altman DG, Pocock SJ, Evans SJ. Reporting of noninferiority and equivalence randomized trials: an extension of the CONSORT statement. Jama. 2006 Mar 8;295(10):1152-60.

3. Rohmel J. Therapeutic equivalence investigations: statistical considerations. Stat Med. 1998 Aug 15-30;17(15-16):1703-14.

4. Siqueira AL, Whitehead A, Todd S. Active-control trials with binary data: a comparison of methods for testing superiority or non-inferiority using the odds ratio. Stat Med. 2008 Feb 10;27(3):353-70.

5. Cheung YB. A modified least-squares regression approach to the estimation of risk difference. Am J Epidemiol. 2007 Dec 1;166(11):1337-44.
