# Supplementary material for: A Non-Inferiority, Individually Randomized Trial of Intermittent Screening and Treatment versus Intermittent Preventive Treatment in the Control of Malaria in Pregnancy
Source: PLoS One. 2015 Aug 10;10(8):e0132247. doi: 10.1371/journal.pone.0132247 (PMC4530893; doi:10.1371/journal.pone.0132247)
Supplement: S5 Text — (DOCX) [file pone.0132247.s024.docx]

# S5 Text. Analysis Plan-Laboratory Findings

**A trial of intermittent preventive treatment with sulfadoxine-pyrimethamine (IPTp-SP) versus intermittent screening and treatment (IST) for the control of malaria in pregnancy.**

**A supplement to the analysis plan**

**Background**

In July 2013, the MA05 investigators provided the DSMB with a locked database related to the clinical components of this trial and asked for permission to analyse these data prior to completion of all the laboratory analyses which, for a variety of reasons, had fallen behind schedule. This request was made so that the important conclusions from this trial could be presented at the Multilateral Initiative on Malaria (MIM) meeting held in Durban in October 2013, the premier malaria meeting held only every 2-3 years. These analyses showed that in terms of the primary trial end points of low birth weight and maternal anaemia, IST is non-inferior to IPTp-SP, even though *Plasmodium falciparum* parasites in each of the four trial areas remain sensitive to SP. A summary of these preliminary findings is presented in the appendix to this document. Laboratory analyses have now been completed and a final database is being compiled which contains data on (a) peripheral blood smears and (b) placental histology, both of which were outstanding when the clinical data base was locked on 30/07/2013.

**Ensuring that blinding was maintained after the code for the clinical database had been broken**

All investigators involved in laboratory analyses, including the scientist who read the placental slides, have remained blind to the study code of the women whose samples they were examining. To ensure further the independence of the blood film readings, an independent data manager not linked to the trial in any way linked the dates on the blood films to the clinical contact dates held in the main (locked) data base. Once pairs of blood films matching a clinical contact date had been identified by this process, an independent statistician not associated with the trial ran a pre-written Stata program to identify blood films that were discrepant according to the criteria being used for the RTS,S malaria vaccine trial, as described by Swysen et al ^27^. The list of discrepant films was returned to the sites and a third reading done. The third reading has been used to create an authoritative result (and density, for positive slides) for each study contact at which a blood film was taken, following the algorithm described by Swysen et al ^27^. In brief, in the case of slides originally discrepant on positivity (i.e. one positive, one negative), the third reading is considered definitive. In the case of slides originally discrepant on parasite density (i.e. both positive, but parasite counts inconsistent), then the geometric mean of the two closest readings (on the log scale) is taken.

**Analysis of blood film results**

Blood film results will be analysed as described in the original analysis plan (as a secondary outcome):

1. *The prevalence of peripheral blood parasitaemia*

- *at the final antenatal follow-up visit (visit 3)*
- *at the time of delivery*
- *at the post-partum follow-up visit*

1. *Incidence of clinical malaria^1^^[[1]](#footnote-1)^*

**Analysis of placental histological findings**

The original analysis plan specified that data on placental malaria would be used for analysis of primary and secondary outcomes as follows:

***Primary outcome 3).*** *Prevalence of placental malaria infection (past, chronic or acute phase) based on placental histology, among women for whom a sample is obtained.*

*Odds ratios will be used for the primary analysis of placental malaria. The non-inferiority margin for risk of placental malaria was specified as 5%, assuming a prevalence of placental malaria in both groups of 25%. As used in the analysis of low birth weight (LBW), this can be re-expressed in terms of the odds ratio of 1.286 ( [30/70] / [25/75] ). This is equivalent to a risk difference of 5.53% at prevalence of 30%, and 6.15% at a prevalence of 40%.*

***Secondary Outcome 4)*** *Placental malaria detected by other methods:*

*The initial analysis plan specified that the prevalence of placental malaria is each treatment group would also be determined using methods other than histology including*

*A positive placental blood film independent of histology*

*A positive RDT of placental blood, independent of histology*

- *A positive placental blood film or RDT of placental blood independent of histology.*

*The sensitivity and specificity of placental blood films and RDTs in diagnosing placental malaria will be calculated.*

**Amendments to definition of placental malaria by histology**

The initial intention was to define placental malaria status following the method of Ismail et al. ^28^ which is a minor modification of the widely used classification of Bulmer et al . [3] The Ismail classification defines the status of the placenta as follows:

- ***not infected*** *(no evidence of parasites or pigment)*
- ***acute infection*** *(parasites present, with absent or minimal pigment deposition within fibrin or cells within fibrin)*
- ***chronic infection*** *(presence of parasites and a significant amount of pigment deposition in fibrin or cells within fibrin)*
- ***past infection*** *(presence of pigment with absence of parasites).*

*An* ***active infection*** *is defined as either an acute or chronic infection, i.e. any sample where parasites are observed.*

A restricted version of the placental histology data base, (which contained no information on treatment group, randomisation or any other outcomes, and from which the ID number was removed) has been reviewed. This preliminary screen showed that, overall, the prevalence of pigment is very high, particularly in the samples from Ghana and Burkina Faso. Differentiation of malaria pigment from formalin pigment that precipitates from the formalin solution in which placental samples are preserved is not possible without the use of birefrigent microscopy. In addition, other precipitates may be mistaken as malaria pigment. Thus, it is likely that some of the examples of ‘malaria pigment’, reported in this, as in many previous studies is artefactual. Misclassification occurring in this way is likely to be non-differential and thus to mask any true differences in the prevalence of malaria infection of the placenta between the two treatment groups. For this reason, we propose the presence of active placental malaria infection (presence of infected maternal erythrocytes of any density), regardless of the level of malaria pigment present, as the primary measure of a malaria infected placenta as this is likely to be the most specific definition. However, we will present also results for the presence of pigment in fibrin or macrophages as shown in the dummy table below. The presence of inflammatory changes will also be tabulated.

Dummy table for placental malaria histology

|  | All | IPTp group (N) | IST group (N) | Odds ratio OR Risk difference^ (95% CI) | p-value |
| --- | --- | --- | --- | --- | --- |
| **Active infections** | % (N) | % (N) | % (N) |  |  |
| Acute infections | % (N) | % (N) | % (N) |  |  |
| Chronic infections | % (N) | % (N) | % (N) |  |  |
|  |  |  |  |  |  |
| **Pigment in fibrin^$^** |  |  |  |  |  |
| None | % (N) | % (N) | % (N) |  |  |
| Mild | % (N) | % (N) | % (N) |  |  |
| Moderate | % (N) | % (N) | % (N) |  |  |
| Abundant | % (N) | % (N) | % (N) |  |  |
|  |  |  |  |  |  |
| **Pigment in macrophages^$^** |  |  |  |  |  |
| None | % (N) | % (N) | % (N) |  |  |
| Mild | % (N) | % (N) | % (N) |  |  |
| Moderate | % (N) | % (N) | % (N) |  |  |
| Abundant | % (N) | % (N) | % (N) |  |  |
|  |  |  |  |  |  |
| **Pigment in fibrin and macrophages^$^** |  |  |  |  |  |
| None | % (N) | % (N) | % (N) |  |  |
| Mild | % (N) | % (N) | % (N) |  |  |
| Moderate | % (N) | % (N) | % (N) |  |  |
| Abundant | % (N) | % (N) | % (N) |  |  |

Acute infection: Infected maternal erythrocytes and no or minimal pigment. Chronic infection: Infected maternal erythrocytes and moderate or abundant pigment. ^$^ Percentages / Numbers shown for children without active infection. ^ Risk differences will be presented if overall prevalence is close to 25%, otherwise the odds ratio will be used.

**References:**

1. Siqueira AL, Whitehead A, Todd S. Active-control trials with binary data: a comparison of methods for testing superiority or non-inferiority using the odds ratio. Stat Med. 2008 Feb 10;27(3):353-70.

2. Gamble C, Ekwaru JP, ter Kuile FO. Insecticide-treated nets for preventing malaria in pregnancy. Cochrane Database Syst Rev. 2006(2):CD003755.

3. Crawley J, Hill J, Yartey J, Robalo M, Serufilira A, Ba-Nguz A, et al. From evidence to action? Challenges to policy change and programme delivery for malaria in pregnancy. Lancet Infect Dis. 2007 Feb;7(2):145-55.

4. ter Kuile FO, van Eijk AM, Filler SJ. Effect of sulfadoxine-pyrimethamine resistance on the efficacy of intermittent preventive therapy for malaria control during pregnancy: a systematic review. JAMA. 2007 Jun 20;297(23):2603-16.

5. Njagi JK, Magnussen P, Estambale B, Ouma J, Mugo B. Prevention of anaemia in pregnancy using insecticide-treated bednets and sulfadoxine-pyrimethamine in a highly malarious area of Kenya: a randomized controlled trial. Trans R Soc Trop Med Hyg. 2003 May-Jun;97(3):277-82.

6. Mbaye A, Richardson K, Balajo B, Dunyo S, Shulman C, Milligan P, et al. A randomized, placebo-controlled trial of intermittent preventive treatment with sulphadoxine-pyrimethamine in Gambian multigravidae. Trop Med Int Health. 2006 Jul;11(7):992-1002.

7. Menendez C, Romagosa C, Ismail MR, Carrilho C, Saute F, Osman N, et al. An autopsy study of maternal mortality in Mozambique: the contribution of infectious diseases. PLoS Med. 2008;5:e44.

8. Singh N, Saxena A, Awadhia SB, Shrivastava R, Singh MP. Evaluation of a rapid diagnostic test for assessing the burden of malaria at delivery in India. Am J Trop Med Hyg. 2005 Nov;73(5):855-8.

9. Leke RF, Djokam RR, Mbu R, Leke RJ, Fogako J, Megnekou R, et al. Detection of the Plasmodium falciparum antigen histidine-rich protein 2 in blood of pregnant women: implications for diagnosing placental malaria. J Clin Microbiol. 1999 Sep;37(9):2992-6.

10. Mankhambo L, Kanjala M, Rudman S, Lema VM, Rogerson SJ. Evaluation of the OptiMAL rapid antigen test and species-specific PCR to detect placental Plasmodium falciparum infection at delivery. J Clin Microbiol. 2002 Jan;40(1):155-8.

11. Mockenhaupt FP, Ulmen U, von Gaertner C, Bedu-Addo G, Bienzle U. Diagnosis of placental malaria. J Clin Microbiol. 2002 Jan;40(1):306-8.

12. Singer LM, Newman RD, Diarra A, Moran AC, Huber CS, Stennies G, et al. Evaluation of a malaria rapid diagnostic test for assessing the burden of malaria during pregnancy. Am J Trop Med Hyg. 2004 May;70(5):481-5.

13. Kilian AH, Kabagambe G, Byamukama W, Langi P, Weis P, von Sonnenburg F. Application of the ParaSight-F dipstick test for malaria diagnosis in a district control program. Acta Trop. 1999 Apr 30;72(3):281-93.

14. VanderJagt TA, Ikeh EI, Ujah IO, Belmonte J, Glew RH, VanderJagt DJ. Comparison of the OptiMAL rapid test and microscopy for detection of malaria in pregnant women in Nigeria. Trop Med Int Health. 2005 Jan;10(1):39-41.

15. Tagbor H, Bruce J, Greenwood B, Chandramohan D. Performance of the OptiMAL® dipstick in the diagnosis of malaria infection in pregnancy. Therapeutics and Clinical Risk Management. 2008;4(3):631 - 6.

16. WHO. Malaria rapid diagnostic test performance: results of WHO product testing of malaria RDTs: Round 1. Geneva: WHO; 2008.

17. Newman RD, Moran AC, Kayentao K, Benga-De E, Yameogo M, Gaye O, et al. Prevention of malaria during pregnancy in West Africa: policy change and the power of subregional action. Trop Med Int Health. 2006;11(4):462 - 9.

18. Coulibaly SO, Nezien D, Traore S, Kone B, Magnussen P. Therapeutic efficacy of sulphadoxine-pyrimethamine and chloroquine for the treatment of uncomplicated malaria in pregnancy in Burkina Faso. Malar J. 2006;5:49.

19. Clerk CA, Bruce J, Affipunguh PK, Mensah N, Hodgson A, Greenwood B, et al. A randomized, controlled trial of intermittent preventive treatment with sulfadoxine-pyrimethamine, amodiaquine, or the combination in pregnant women in Ghana. J Infect Dis. 2008 Oct 15;198(8):1202-11.

20. Clerk CA, Bruce J, Greenwood B, Chandramohan D. The epidemiology of malaria among pregnant women attending antenatal clinics in an area with intense and highly seasonal malaria transmission in northern Ghana. Trop Med Int Health. 2009 Jun;14(6):688-95.

21. Rogerson SJ, Mkundika P, Kanjala MK. Diagnosis of Plasmodium falciparum malaria at delivery: comparison of blood film preparation methods and of blood films with histology. J Clin Microbiol. 2003;41:1370 - 4.

22. Romagosa C, Menendez C, Ismail MR, Quinto L, Ferrer B, Alonso PL, et al. Polarisation microscopy increases the sensitivity of hemozoin and Plasmodium detection in the histological assessment of placental malaria. Acta Trop. 2004 May;90(3):277-84.

23. Landis SH, Ananth CV, Lokomba V, Hartmann KE, Thorp JM, Jr., Horton A, et al. Ultrasound-derived fetal size nomogram for a sub-Saharan African population: a longitudinal study. Ultrasound Obstet Gynecol. 2009 Oct;34(4):379-86. 24

24. Piaggio G, Elbourne DR, Altman DG, Pocock SJ, Evans SJ. Reporting of noninferiority and equivalence randomized trials: an extension of the CONSORT statement. Jama. 2006 Mar 8;295(10):1152-60.

25. Rohmel J. Therapeutic equivalence investigations: statistical considerations. Stat Med. 1998 Aug 15-30;17(15-16):1703-14.

26. Cheung YB. A modified least-squares regression approach to the estimation of risk difference. Am J Epidemiol. 2007 Dec 1;166(11):1337-44.

27. Swysen C, Vekemans J, Bruls M, Oyakhirome S, Drakeley C, Kremsner P, et al. Development of standardized laboratory methods and quality processes for a phase III study of the RTS, S/AS01 candidate malaria vaccine. Malar J. 2011;10:223.28

28. Ismail MR, Ordi J, Menendez C, Ventura PJ, Aponte JJ, Kahigwa E, et al. Placental pathology in malaria: a histological, immunohistochemical, and quantitative study. Hum Pathol. 2000 Jan;31(1):85-93.

1. Defined as an illness accompanied by malaria parasitaemia occurring outside a routine antenatal clinic. [↑](#footnote-ref-1)
